# Noonindoles A–F: Rare Indole Diterpene Amino Acid Conjugates from a Marine-Derived Fungus, *Aspergillus noonimiae* CMB-M0339

**DOI:** 10.3390/md20110698

**Published:** 2022-11-07

**Authors:** Sarani Kankanamge, Zeinab G. Khalil, Paul V. Bernhardt, Robert J. Capon

**Affiliations:** 1 Institute for Molecular Bioscience, The University of Queensland, St Lucia, QLD 4072, Australia; 2 School of Chemistry and Molecular Bioscience, The University of Queensland, St Lucia, QLD 4072, Australia

**Keywords:** indole diterpene, noonindole, marine-derived fungus, *Aspergillus noonimiae*, antifungal, Australia, microbial biodiscovery, molecular network chemical profiling, MATRIX cultivation profiling

## Abstract

Analytical scale chemical/cultivation profiling prioritized the Australian marine-derived fungus *Aspergillus noonimiae* CMB-M0339. Subsequent investigation permitted isolation of noonindoles A–F (**5**–**10**) and detection of eight minor analogues (**i**–**viii**) as new examples of a rare class of indole diterpene (IDT) amino acid conjugate, indicative of an acyl amino acid transferase capable of incorporating a diverse range of amino acid residues. Structures for **5**–**10** were assigned by detailed spectroscopic and X-ray crystallographic analysis. The metabolites **5**–**14** exhibited no antibacterial properties against G-ve and G+ve bacteria or the fungus *Candida albicans*, with the exception of **5** which exhibited moderate antifungal activity.

## 1. Introduction

**Figure 1:**
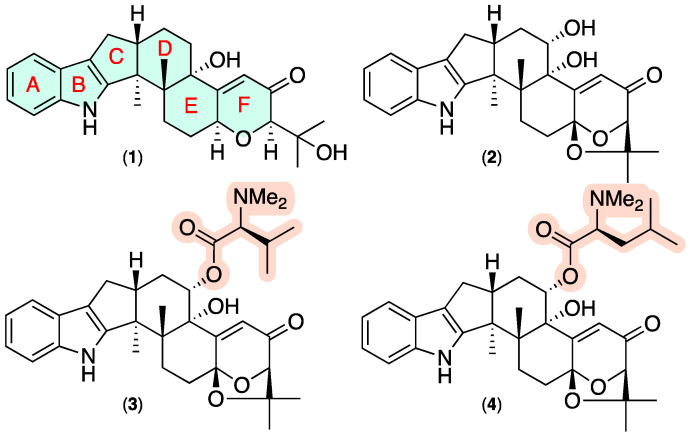
Known fungal IDTs highlighting the paxilline scaffold (blue) and rare amino acid acyl functionality (tan).

Since the indole diterpene (IDT) paxilline (**1**) (Figure 1) was first described in 1975 as a tremorgenic agent of *Penicillium paxilli* [1], a number of fungal natural products sharing this fused hexacyclic skeleton have been reported (i.e., paxillines, paspalines, paspalinines, paspalicine, paspalitrems, penijanthine A, penitrems, aflatrem, penerpenes, sulpinines, and terpendoles) from a wide range of genera, including from terrestrially sourced *Acremonium* [2], *Albophoma* [3,4,5], *Aspergillus* [6,7,8,9,10], *Chaunopycnis* [11], *Claviceps* [12], *Emericella* [13,14], *Eupenicillium* [15], *Neotyphodium* [16], *Penicillium* [2,17,18,19,20,21], *Phomopsis* [22], and marine-derived *Penicillium* [23,24,25,26,27]. In addition to tremorgenic properties [1,2,6,7,13,14,17,22,28], members of this IDT family have been reported to exhibit anticancer [23,29], anti-insectan [8,9,15] and anti-H1N1 [24] activity, with Merck researchers reporting potent and selective potassium ion channel antagonist properties [28] prompting patent protection for the treatment of neurodegenerative diseases (i.e., Alzheimer’s disease) [30] and glaucoma [31]. Paxilline (**1**) has been noted as one of the most potent and selective nonpeptidergic inhibitors of large-conductance, voltage, and Ca^2+^dependent BK-type potassium channels [32], stimulating interest in chemical synthesis [33,34,35] and biosynthesis [36,37,38,39,40,41,42] across this structure class. For example, a 2022 report [43] described two genes encoding monomodular nonribosomal peptide synthetase (NRPS)-*like* enzymes that catalyse the acylation of 14-hydroxypaspalinine (**2**) (Figure 1) to the only two known natural product examples of amino acid conjugated IDTs: 14-(*N*,*N*-dimethyl-L-valyloxy)paspalinine (**3**) (Figure 1) reported [9] in 1993 from *Aspergillus nominus* NRRL 13,137 and patented in 1993 as an anti-insectan for controlling Coleopteran and Lepidopteran insects [44], and in 2003 for the treatment of glaucoma [31]; and 14-(*N*,*N*-dimethyl-l-leucyloxy)paspalinine (**4**) patented in 2003 for the treatment of glaucoma [31] and subsequently optimised for production from *Aspergillus alliaceus* [10].

This current report describes our discovery of new IDT amino acid conjugates produced by the Australian marine-derived fungus *Aspergillus noonimiae* CMB-M0339. Using an integrated strategy of chemical and cultivation profiling to both prioritize and facilitate production, we successfully detected, isolated, characterised, and identified five new IDT amino acid conjugates and a key hydroxy precursor, noonindoles A–F (**5**–**10**), together with the four known analogues paspaline (**11**) [12], paspaline B (**12**) [18], the carboxylic acid (**13**) [24], and emindole SB (**14**) [13]. Structures for **5**–**14** were assigned by detailed spectroscopic and X-ray analysis as summarised below, along with a plausible biogenetic relationship. We also made use of biosynthetic considerations and diagnostic MS/MS fragmentations to tentatively assign structures to a series of eight minor IDT amino acid co-metabolites (**i**–**viii**).

## 2. Results

**Figure 2:**
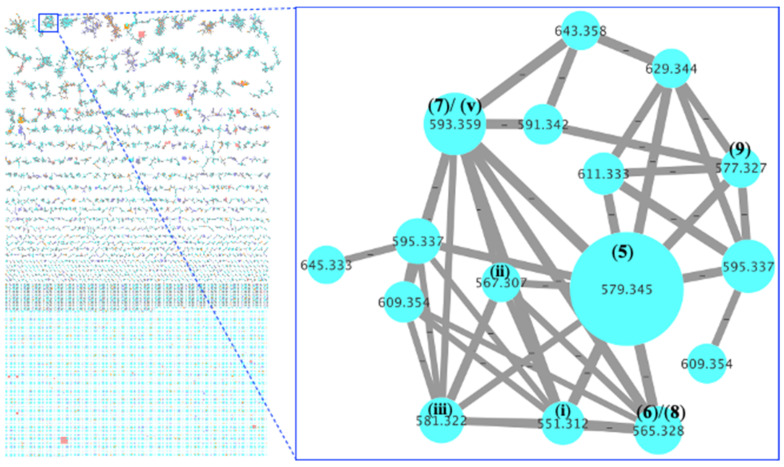
GNPS molecular network of an inhouse fungal extract library, revealing a unique cluster of metabolites (expansion) associated exclusively with strain CMB-M0339.

**Figure 3:**
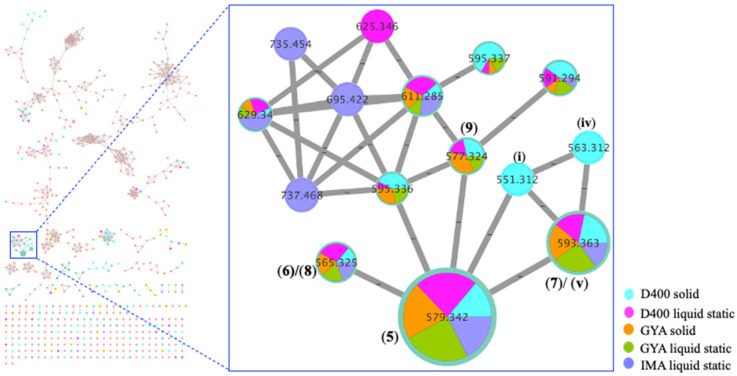
GNPS molecular network of a set of MATRIX extracts of CMB-M0339 showing production of noonindoles A–E (**5**–**9**) and related minor metabolites under selected culture conditions.

**Figure 4:**
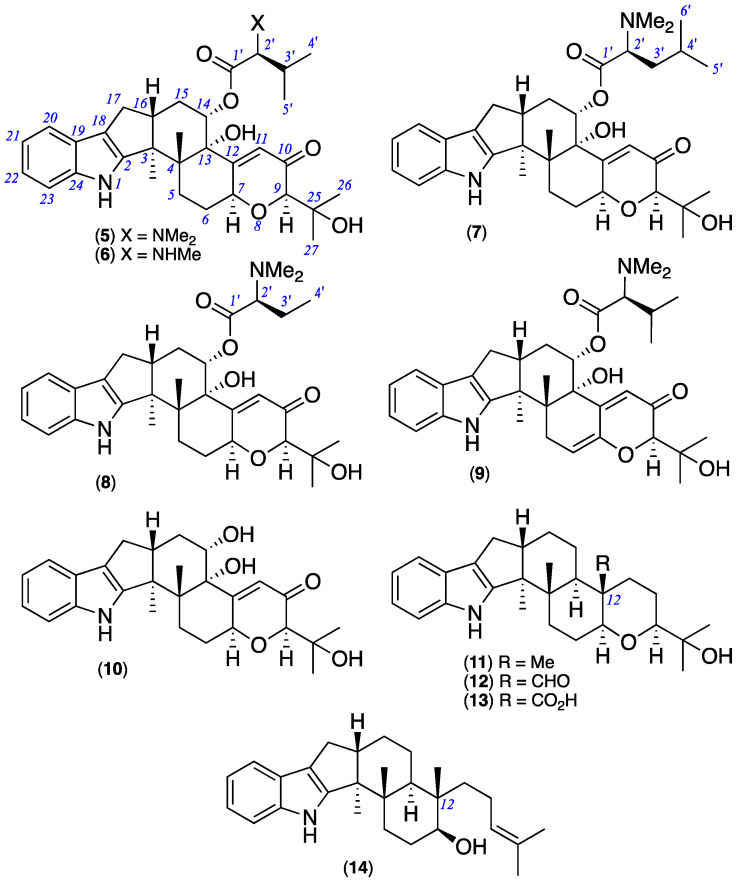
New noonindoles A–F (**5**–**10**) and known **11**–**14**.

An EtOAc extract was prepared from a 3.3% saline M1 agar plate cultivation of the marine-derived fungus *Aspergillus noonimiae* CMB-M0339. UPLC-DAD (Figure S6) was subjected to a global natural product social (GNPS) [45] molecular network (Figure 2) analysis to reveal peaks/nodes for three prominent and structurally related metabolites: **5** (*m*/*z* 579 (M+H), C_34_H_46_N_2_O_6_); **6** (*m*/*z* 565 (M+H), C_33_H_44_N_2_O_6_); and **7** (*m*/*z* 593 (M+H), C_35_H_48_N_2_O_6_). Online database searching suggested these metabolites were unprecedented in the natural products literature. Subsequent cultivation profiling using a miniaturized 24-well plate microbioreactor methodology (MATRIX) [46] employing ×11 different media under solid agar (2 mL), as well as static and shaken broth (1.5 mL) conditions (Figure S7) supported by UPLC-DAD and GNPS chemical profiling (Figure 3 and Figure S8) confirmed D400 solid phase agar as the optimal culture condition. The EtOAc extract of a ×300 plate D400 solid phase 12 day cultivation was subjected to solvent trituration and reversed-phase HPLC (Figure S9) to yield **5**–**14** (Figure 4). Detailed spectroscopic analysis successfully identified the known natural products paspaline (**11**) [12], paspaline B (**12**) [18], the carboxylic acid **13** [24], and emindole SB (**14**) [13] (Tables S8–S11, Figures S50–S68). Further spectroscopic analysis identified the new noonindoles A–F (**5**–**10**) as summarised below.

**Figure 5:**
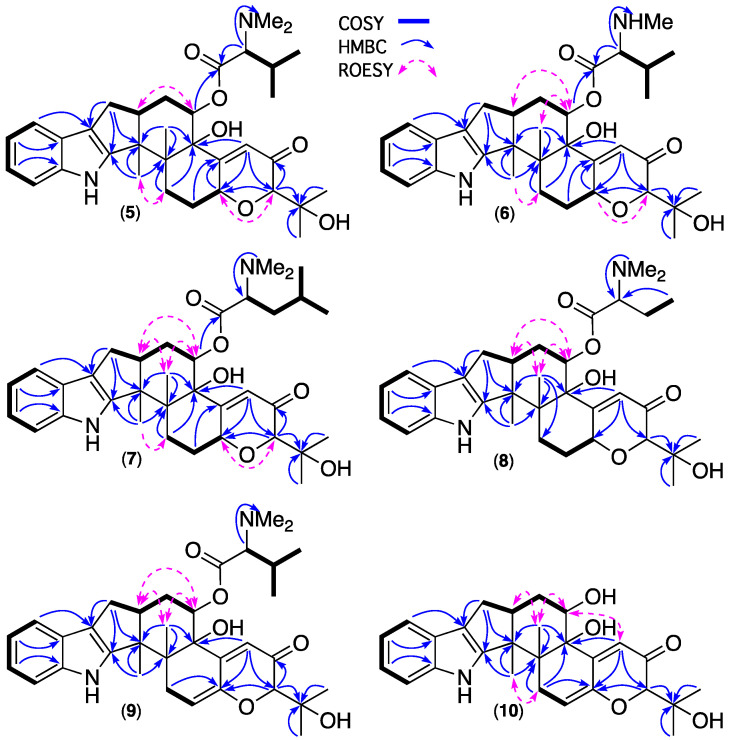
2D NMR (methanol-*d*_4_) correlations for **5**–**10**.

**Figure 6:**
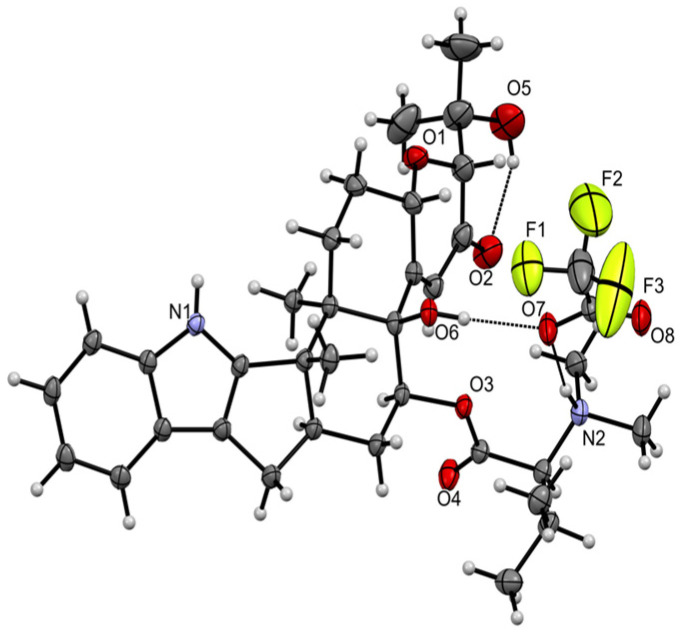
X-ray crystal structure of noonindole A (**5**).

HRESIMS analysis of **5** revealed a molecular formula (C_34_H_46_N_2_O_6_, Δmmu +2.7) requiring thirteen double bond equivalents (DBE). The NMR (methanol-*d*_4_) data for **5** (Table 1 and Table S2, and Figures S10–S15) disclosed resonances for ten sp^2^ olefinic carbons and two sp^2^ ester/lactone carbonyls, accounting for seven DBE and requiring that **5** be hexacyclic, while diagnostic 2D NMR correlations (Figure 5) established a carbon/hetero skeleton in common with **1** further annotated by an *N*,*N*-dimethyl-valinyloxy ester pendant to C-14. The structure and absolute configuration for noonindole A (**5**) were confirmed by a single crystal X-ray analysis (Figure 6 and Table S13).

HRESIMS analysis of **6**–**8** revealed molecular formulae (C_33_H_44_N_2_O_6_, Δmmu +2.0; C_35_H_48_N_2_O_6_, Δmmu +2.6; C_33_H_44_N_2_O_6_, Δmmu +1.5) consistent with lower/higher homologues of **5**. Comparison of the NMR (methanol-*d*_4_) data for **6** (Table 1 and Table S3, Figures S17–S22) with **5** allowed the principle differences to be attributed to replacement of the *N*,*N*-dimethyl-valinyloxy side chain in **5** (δ_H_ 2.90, s; δ_C_ 43.1) with an *N*-methyl-valinyloxy side chain in **6** (δ_H_ 2.72, s, NH(CH_3_); δ_C_ 33.8, NH(CH_3_)). Likewise, comparison of the NMR (methanol-*d*_4_) data for **7** (Table 1 and Table S4, and Figures S24–S29) and **8** (Table 2 and Table S5, and Figures S31–S35) with **5** allowed the principle differences to be attributed to replacement of the *N*,*N*-dimethyl-valinyloxy moiety in **5** with an *N*,*N*-dimethyl-leucinyloxy moiety in **7** and an *N*,*N*-dimethyl-homoalaninyloxy in **8**. These conclusions were reinforced by diagnostic 2D NMR correlations (Figure 5) which, together with biogenetic considerations, allowed assignment of structures to noonindoles B–D (**6**–**8**).

HRESIMS analysis of **9** revealed a molecular formula (C_34_H_44_N_2_O_6_, Δmmu +1.2) consistent with an oxidised analogue of **5**. Comparison of the NMR (methanol-*d*_4_) data for **9** (Table 2 and Table S6, and Figures S37–S41) with **5** allowed the principle differences to be attributed to replacement of the sp^3^ H-7/C- 7 methine in **5** (δ_H_ 4.91, H-7; δ_C_ 74.6, C-7) with an sp^2^ quaternary C-7 and sp^2^ H-6/C-6 methine in **9** (δ_H_ 5.74, m, H-6; δ_C_ 113.5, C-6; δ_C_ 146.4, C-7), consistent with incorporation of a Δ^6,7^. These conclusions were reinforced by 2D NMR correlations (Figure 5) which, together with biogenetic considerations, allowed assignment of the structure for noonindole E (**9**).

HRESIMS analysis of **10** revealed a molecular formula (C_27_H_33_NO_5_, Δmmu +1.1) consistent with a hydrolysed analogue of **5** lacking the *N*,*N*-dimethyl-valinyloxy ester side chain. This hypothesis was confirmed on comparison of the NMR (methanol-*d*_4_) data for **10** (Table 2 and Table S7, and Figures S43–S48) with **5** which revealed the absence of resonances for the *N*,*N*-dimethyl-valinyloxy moiety and a significant shielding of the resonance for H-14 in **10** (δ_H_ 4.16) compared with **5** (δ_H_ 5.53). These conclusions were reinforced by 2D NMR correlations (Figure 5) which, together with biogenetic considerations, allowed assignment of a structure for noonindole F (**10**).

Co-isolation of noonindoles A–F (**5**–**10**) and the known IDTs **11**–**14** together with an X-ray crystal analysis of **5** supported a common absolute configuration across the hexacyclic indole terpene core, and established the configuration of the *N*,*N*-dimethyl-l-valinyloxy moiety in **5**. Although low yields combined with *N*-alkylation precluded hydrolysis and independent assignment of the amino acid residue absolute configuration in **6**–**9** (i.e., Marfey’s analysis), an amino acid l configuration across **6**–**9** was proposed based on the likelihood of a common NRPS-*like* aminoacyl modifying enzyme in the noonindole biosynthetic gene cluster (BGC) (see below).

The metabolites **5**–**14** did not inhibit the growth of human colon (SW620) or lung (NCI-H460) carcinoma cells (IC_50_ > 30 μM) (Figure S81), or the fungus *Candida albicans* ATCC10231, the Gram-negative bacterium *Escherichia coli* ATCC11775, or the Gram-positive bacteria *Staphylococcus aureus* ATCC25923 or *Bacillus subtilis* ATCC6633 (IC_50_ > 30 μM), with the exception of noonindole A (**5**) which displayed modest antifungal activity (IC_50_~5 μM) (Figure S80). This lack of cellular toxicity bodes well for future (ongoing) evaluation of noonindole ion channel inhibitory pharmacology.

**Figure 7:**
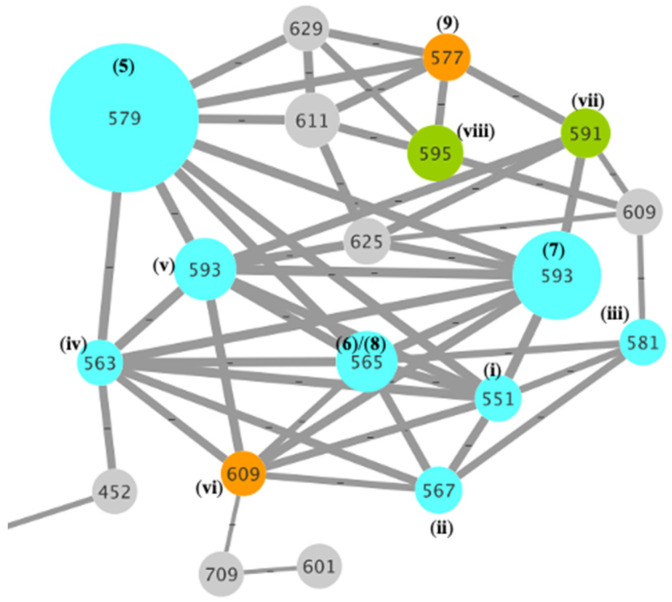
GNPS cluster of the EtOAc extract of a D400 (MATRIX) culture of CMB-M0339 revealing **5**–**9** along with closely associated nodes for the minor analogues **i**–**viii**.

**Figure 8:**
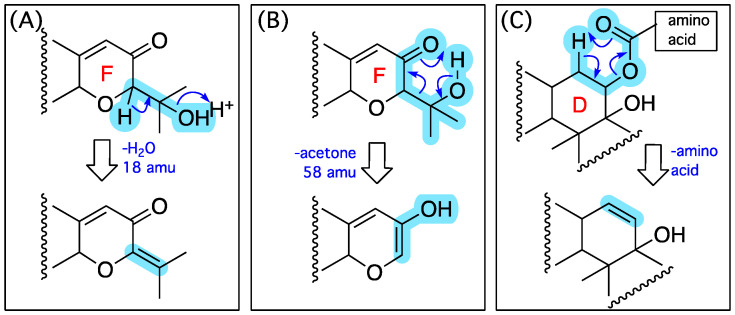
MS/MS fragmentations common to **1**–**5** and minor co-metabolites **i**–**viii**; (**A**) loss of water, (**B**) retro-Aldol loss of acetone and, (**C**) loss of the amino acid residue.

GNPS analysis of the EtOAc extract of the analytical scale D400 (MATRIX) culture of CMB-M0339 detected **5**–**9** along with associated nodes for a selection of putative minor analogues (Figure 7). The MS/MS spectra for **5**–**9** (Figures S72–S75) revealed three common fragmentations attributed to loss of water (Figure 8A), retro-Aldol loss of acetone (Figure 8B), and loss of the amino acid residue (Figure 8C). While low yields precluded isolation of the minor analogues **i**–**viii**, diagnostic MS/MS fragmentations and high-resolution mass measurements (i.e., molecular formulae) permitted tentative assignments for **i**–**v** (Figures S74, S76–S79, Table S12) and on the basis of GNPS co-clustering and biosynthetic considerations to **vi**–**viii**, albeit with some allowance isomeric alternatives (Table 3 and Table S12). The diversity of IDT amino acid conjugates produced by CMB-M0339 is in stark contrast to existing knowledge, which is limited to **3** from *Aspergillus nomius* [9] and **4** from *A. alliaceus* [10]. Unlike these earlier published accounts, it appears CMB-M0339 employs an NRPS-*like* aminoacylation enzyme with an adenylation domain tolerant of different amino acid substrates (i.e., Val, Leu, Ile, Pro, Ser, Thr, and homo-Ala).

**Figure 9:**
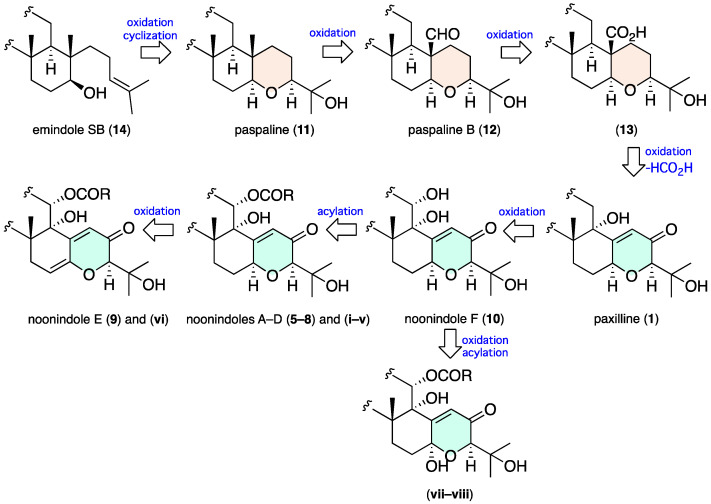
Plausible biogenetic relationship linking **5**–**14** (and **1**) and inclusive of the minor co-metabolites **i**–**viii**.

A preliminary assessment of the noonindole biosynthetic gene cluster (BGC) suggests a biogenetic relationship linking **5**–**14** and inclusive of the minor co-metabolites **i**–**viii** starting with emindole SB (**14**) undergoing stereospecific epoxidation and ring closure to paspaline (**11**) followed by sequential oxidation to paspaline B (**12**) and the carboxylic acid **13**, followed by decarboxylation and oxidation to paxilline (**1**) (Figure 9). Oxidation of **1** could then yield noonindole F (**10**) with further oxidation and/or amino acid acylation returning noonindoles A–E (**5**–**9**) and co-clustering minor analogues (**i**–**viii**). Consistent with this hypothesis, close examination of the CMB-M0339 D400 extract GNPS and UPLC-DAD-MS data using single ion extraction (SIE) detected an ion with a molecular formula attributable to **1** (Figure S71).

Our investigation into the marine-derived *Aspergillus noonimiae* CMB-M0339 led to the discovery of noonindoles A–F (**5**–**10**) and related minor analogues (**i**–**viii**) as new examples of a rare class of fungal indole diterpene amino acid conjugate. This discovery highlights the continued capacity of fungi to provide access to new chemical space and validates molecular networking (GNPS) as an effective platform to detect, dereplicate, and prioritize new over known chemistry, and cultivation profiling (MATRIX) as a means to optimise the production. Our discovery of the noonindoles suggests the CMB-M0339 features an NRPS-*like* aminoacyl modifying enzyme in the noonindole biosynthetic gene cluster (BGC) capable of accommodating and incorporating multiple lipophilic amino acids. Further studies into the structure, biosynthesis, and biology of these and other CMB-M0339 indole diterpenes are ongoing, and will be reported elsewhere.

## 3. Materials and Methods

### 3.1. General Experimental Procedures

Chemicals were purchased from Sigma-Aldrich or Merck unless otherwise specified. Solvent extractions were performed using analytical-grade solvents, while HPLC, UPLC, and HPLC-MS analyses employed HPLC-grade solvents supplied by Labscan or Sigma-Aldrich and filtered/degassed through 0.45 μm polytetrafluoroethylene (PTFE) membrane prior to use. Deuterated solvents were purchased from Cambridge Isotopes (Tewksbury, MA, USA). Microorganisms were manipulated under sterile conditions in a Laftech class II biological safety cabinet and incubated in either an MMM Friocell incubator (Lomb Scientific, NSW, Australia) or an Innova 42R incubator shaker (John Morris, NSW, Australia) at 26.5 °C. Semi-preparative and preparative HPLCs were performed using Agilent 1100 series HPLC instruments with corresponding detectors, fraction collectors, and software. Analytical UPLC chromatograms were obtained on an Agilent 1290 infinity UPLC instrument equipped with a diode array multiple wavelength detector (Zorbax C_8_ RRHD 1.8 μm, 50 × 2.1 mm column, gradient elution at 0.417 mL/min over 2.50 min from 90% H_2_O/MeCN to 100% MeCN with isocratic 0.01% TFA/MeCN modifier). UPLC-QTOF analyses were performed on an Agilent 6545 Q-TOF instrument incorporating an Agilent 1290 Infinity II UHPLC (Zorbax C_8_ RRHD 1.8 μm, 50 × 2.1 mm column, gradient elution at 0.417 mL/min over 2.5 min from 90% H_2_O/MeCN to 100% MeCN with isocratic 0.1% formic acid/MeCN modifier). Chiroptical measurements ([α]_D_) were obtained on a JASCO P-1010 polarimeter in a 100 × 2 mm cell at specified temperatures. Nuclear magnetic resonance (NMR) spectra were acquired on a Bruker Avance 600 MHz spectrometer with either a 5 mm PASEL 1H/D-13C Z-Gradient probe or 5 mm CPTCI 1H/19F-13C/15N/DZ-Gradient cryoprobe, controlled by TopSpin 2.1 software, at 25 °C in either methanol-*d*_4_, CDCl_3_, or DMSO-*d*_6_, with referencing to residual ^1^H or ^13^C solvent resonances (methanol-*d*_4_: δ_H_ 3.31 and δ_C_ 49.15; CDCl_3_: δ_H_ 7.24 and δ_C_ 77.23; DMSO-*d*_6_: δ_H_ 2.50 and δ_C_ 39.50). High-resolution ESIMS spectra were obtained on a Bruker micrOTOF mass spectrometer by direct injection in MeOH at 3 μL/min using sodium formate clusters as an internal calibrant. Structural assignments were made with additional information from gCOSY, gHSQC, and gHMBC experiments.

### 3.2. Fungal Isolation and DNA Taxonomic Analysis

A marine sediment collected in 2008 from a location off Perth, Western Australia, was used to inoculate an M1 agar plate (inclusive of 3.3% artificial sea salt) which was incubated at 27 °C for 10–14 days, after which colony selection yielded an array of isolates including fungus CMB-M0339. Genomic DNA was extracted from the mycelia of CMB-M0339 using the DNeasy Plant Mini Kit (Qiagen) as per the manufacturers protocol, and the 18s rRNA genes were amplified by PCR using the universal primers ITS-1 (5′-TCCGTAGGTGAACCTGCGG-3′) and ITS-4 (5′-TCCTCCGCTTATTGATATGC-3′) purchased from Sigma-Aldrich. The PCR mixture (50 μL) containing 1 μL of genomic DNA (20–40 ng), 200 μM of each deoxynucleoside triphosphate (dNTP), 1.5 mM MgCl_2_, 0.3 μM of each primer, 1 U of *Taq* DNA polymerase (Fisher Biotec), and 5 μL of PCR buffer was amplified using the following conditions: initial denaturation at 95 °C for 3 min, 30 cycles in series of 94 °C for 30 s (denaturation), 55 °C for 60 s (annealing), and 72 °C for 60 s (extension), followed by one cycle at 72 °C for 6 min. PCR products were purified with PCR purification kit (Qiagen, Victoria, Australia) and examined by agarose gel electrophoresis, with DNA sequencing performed by the Australian Genome Research Facility (AGRF) at The University of Queensland. A BLAST analysis (NCBI database) on the resulting CMB-M0339 ITS gene sequence (Figures S1–S3, GenBank accession no. OP132523) revealed 92.5% identity with the fungal strain *Aspergillus noonimiae.*

### 3.3. Global Natural Product Social (GNPS) Molecular Networking

Aliquots (1 μL) of CMB-M0339 cultivation extract (100 μg/mL in MeOH) were analysed on an Agilent 6545 Q-TOF LC/MS equipped with an Agilent 1290 Infinity II UPLC system (Zorbax C_8_, 0.21 μm, 1.8 × 50 mm column, gradient elution at 0.417 mL/min over 2.5 min from 90% H_2_O/MeCN to MeCN with an isocratic 0.1% formic acid/MeCN modifier). UPLC-QTOF-(+) MS/MS data acquired for all samples at a collision energy of 35 eV were converted from Agilent MassHunter data files (d) to mzXML file format using MSConvert software, and transferred to the GNPS server (gnps.ucsd.edu). Molecular networking was performed using the GNPS data analysis workflow [45] employing the spectral clustering algorithm with a cosine score of 0.5 and a minimum of 6 matched peaks. The resulting spectral network was imported into Cytoscape version 3.7.1 [47] and visualized using a ball-and-stick layout where nodes represent parent mass and cosine score was reflected by edge thickness. Moreover, group abundances were set as pie charts, which reflected the intensity of MS signals. MS/MS fragmentation analysis was performed on the same machine for ion detected in the full scan range at an intensity above 1000 counts at ten scans/s, with an isolation width of 4~*m*/*z* using fixed collision energy and a maximum of 3 selected precursors per cycle. General instrument parameters including gas temperature at 325 °C, drying gas 10 L/min, nebulizer 20 psig, sheath gas temperature 400 °C, fragmentation Volta 180eV, and skimmer 45 eV.

### 3.4. MATRIX Cultivation Profiling

The fungus CMB-M0339 was cultured in a 24-well plate microbioreactor under ×11 different media for 10–14 days in solid phase (27 °C), as well as in static (30 °C) and shaken broths (30 °C, 190 rpm) [46], with regular monitoring of growth (Figure S7). At this point, wells were individually extracted with EtOAc (2 mL), and the organic phase was centrifuged (13,000 rpm, 3 min) and dried under N_2_ at 40 °C to yield ×33 extracts. Individual extracts were redissolved in MeOH (30 μL) containing calibrant (2,4-dinitrophenoldecane ether, 50 μg/mL), and aliquots (1 μL) were subjected to: (i) UPLC-DAD analysis (Zorbax C_8_ 1.8 μm, 2.1 × 50 mm column, gradient elution at 0.417 mL/min over 2.52 min from 90% H_2_O/MeCN to 100% MeCN followed by 0.83 min isocratic elution with MeCN, inclusive of an isocratic 0.01% TFA/MeCN modifier) (Figure S8); and (ii) GNPS analysis (Figure 3). This process identified solid phase D400 as the optimal culture conditions for producing targeted CMB-M0339 natural products.

### 3.5. Scale Up Cultivation and Fractionation

The fungus CMB-M0339 was cultivated on D400 agar (×300 plates) at 27 °C for 10–14 days after which the agar and fungal mycelia were harvested and extracted with EtOAc (2 × 5 L), and the combined organic phase was filtered and concentrated in vacuo at 40 °C to yield an extract (2.9 g). This extract was sequentially triturated with *n*-hexane (20 mL), CH_2_Cl_2_ (20 mL), MeOH (20 mL), and concentrated in vacuo to afford *n*-hexane (961.8 mg), CH_2_Cl_2_ (1783.8 mg), and MeOH (80.3 mg) soluble fractions. A portion of the CH_2_Cl_2_ soluble fraction (1363 mg) was subjected to preparative reversed-phase HPLC (Phenomenex Luna-C_8_ 10 μm, 21.2 × 250 mm column, with gradient elution at 20 mL/min over 20 min from 90% H_2_O/MeCN to 100% MeCN with constant 0.1% TFA/MeCN modifier) to yield noonindole A (**5**) (R_f_ 15.7 min, 83.5 mg, 4.3%). The remaining mixed fractions were subjected to semi-preparative reversed-phase HPLC to yield noonindole B (**6**) (R_f_ 18.3 min, 2.0 mg, 0.1%) (semi-preparative HPLC (Zorbax C_8_ 5mm column, 9.4 × 250 mm, 3 mL/min isocratic elution of 37% MeCN/H_2_O over 20 min with constant 0.1% TFA modifier)); noonindole F (**10**) (R_f_ 19.9 min, 0.7 mg, 0.03%) (semi-preparative HPLC (Agilent C_8_-Ep 5mm column, 9.4 × 250 mm, 3 mL/min isocratic elution of 50% MeCN/H_2_O over 25 min with constant 0.1% TFA modifier)); noonindole D (**8**) (R_f_ 28.6 min, 0.4 mg, 0.02%) (semi-preparative HPLC (Zorbax C_18_ 5mm column, 9.4 × 250 mm, 3 mL/min isocratic elution of 40% MeCN/H_2_O over 30 min with constant 0.1% TFA modifier)); noonindole E (**9**) (R_f_ 20.9 min, 0.8 mg, 0.04%) (semi-preparative HPLC (Zorbax C_18_ 5mm column, 9.4 × 250 mm, 3 mL/min isocratic elution of 40% MeCN/H_2_O over 30 min with constant 0.1% TFA modifier)); 12-demethylpaspaline-12-carboxylic acid (**13**) (R_f_ 9.8 min, 1.6 mg, 0.08%) (semi-preparative HPLC (Zorbax C_18_ 5mm column, 9.4 × 250 mm, 3 mL/min isocratic elution of 85% MeCN/H_2_O over 15 min with constant 0.1% TFA modifier)); paspaline B (**12**) (R_f_ 26.1 min, 0.3 mg, 0.01%) (semi-preparative HPLC (Agilent C_8_-Ep 5mm column, 9.4 × 250 mm, 3 mL/min isocratic elution of 60% MeCN/H_2_O over 25 min with constant 0.1% TFA modifier)); paspaline (**11**) (R_f_ 17.8 min, 1.2 mg, 0.06%) (Semi-preparative HPLC (Agilent CN 5mm column, 9.4 × 250 mm, 3 mL/min isocratic elution of 60% MeCN/H_2_O over 20 min with constant 0.1% TFA modifier)); emindole SB (**14**) (R_f_ 19.7 min, 1.2 mg, 0.06%) (semi-preparative HPLC (Agilent CN 5mm column, 9.4 × 250 mm, 3 mL/min isocratic elution of 60% MeCN/H_2_O over 20 min with constant 0.1% TFA modifier)); and solid-phase extraction (Sep-Pak (Agilent Bond Elut C_18_ cartridge, 5 g) gradient elution from 90% H_2_O/MeCN to 100% MeCN) and semi-preparative reversed-phase HPLC (Zorbax C_8_ 5mm column, 9.4 × 250 mm, 3 mL/min isocratic elution of 40% MeCN/H_2_O over 20 min with constant 0.1% TFA modifier) to yield noonindole C (**7**) (R_f_ 21.9 min, 2.0 mg, 0.1%) (Figure S9). (Note: All % yields are weight to weight estimates based on unfractionated EtOAc extract).

### 3.6. Characterization of Metabolites ***5**–**14***

*noonindole A* (**5**); pale yellow solid; [α]D^21^–18 (*c* 0.02, MeOH); NMR (600 MHz, methanol-*d*_4_), see Table S2 and Figures S10–S15; HRMS (ESI) *m*/*z*: [M+H]^+^ calcd for C_34_H_47_N_2_O_6_ 579.3429; found 579.3456.
*noonindole B* (**6**); white solid; [α]D^21^–13 (*c* 0.08, MeOH); NMR (600 MHz, methanol-*d*_4_), see Table S3 and Figures S17–S22; HRMS (ESI) *m*/*z*: [M+H]^+^ calcd for C_33_H_45_N_2_O_6_, 565.3272; found 565.3292.
*noonindole C* (**7**); white solid; [α]D^21^–9 (*c* 0.06, MeOH); NMR (600 MHz, methanol-*d*_4_), see Table S4 and Figures S24–S29; HRMS (ESI) *m*/*z* [M+H]^+^ calcd for C_35_H_49_N_2_O_6_, 593.3585; found 593.3611.
*noonindole D* (**8**); white solid; [α]D^23^–30 (*c* 0.03, MeOH); NMR (600 MHz, methanol-*d*_4_), see Table S5 and Figures S31–S35; HRMS (ESI) *m*/*z* [M+H]^+^ calcd for C_33_H_45_N_2_O_6_, 565.3272; found 565.3287.
*noonindole E* (**9**); white solid; [α]D^23^–13 (*c* 0.06, MeOH); NMR (600 MHz, methanol-*d*_4_), see Table S6 and Figures S37–S41; HRMS (ESI) *m*/*z* [M+H]^+^ calcd for C_34_H_45_N_2_O_6_, 577.3272; found 577.3284.
*noonindole F* (**10**); white solid; [α]D^21^–18 (*c* 0.02, MeOH); NMR (600 MHz, methanol-*d*_4_), see Table S7 and Figures S43–S48; HRMS (ESI) *m*/*z* [M+Na]^+^ calcd for C_27_H_33_NO_5_Na, 474.2251; found 474.2262.
*paspaline* (**11**); white solid; [α]D^22^–21 (*c* 0.09, CHCl_3_); [24] NMR (600 MHz, DMSO-*d*_6_), see Table S8 and Figures S50–S53; [24] HRMS (ESI) *m*/*z* [M+H]^+^ calcd for C_28_H_40_NO_2_, 422.3054; found 422.3071.
*paspaline B* (**12**) white solid; [α]D^22^–24 (*c* 0.02, CHCl_3_); [18] NMR (600 MHz, CDCl_3_), see Table S9 and Figures S55–S58; [18] HRMS (ESI) *m*/*z* [M+Na]^+^ calcd for C_28_H_37_NO_3_Na, 458.2666; found 458.2680.
12-demethylpaspaline-12-carboxylic acid (**13**); white solid; [α]D^22^ + 37 (*c* 0.01, CHCl_3_); [24] NMR (600 MHz, DMSO-*d*_6_), see Table S10 and Figures S60–S63; [24] HRMS (ESI) *m*/*z* [M+H]^+^ calcd for C_28_H_38_NO_4_,452.2795; found 452.2807.
*emindole SB* (**14**); white solid; [α]D^21^–18 (c 0.05, CHCl_3_); [24] NMR (600 MHz, DMSO-*d*_6_), see Table S11 and Figures S65–S68; [24] HRMS (ESI) *m*/*z* [M+H]^+^ calcd for C_28_H_40_NO, 406.3104; found 406.3126.

### 3.7. Phylogenetic Comparison of CMB-M0339 with Fungi Reported to Produce Biosynthetically Related Indole Terpenes

Phylogenetic tree obtained by PhyML Maximum Likelihood analysis was constructed using the top similar 18S rRNA sequences displayed after BLAST on Refseq RNA NCBI database using CMB-M0339 18S rRNA as queries (Figure S4). The JC69 model was used to infer phylogeny sequences [48]. Sequence alignments were produced with the MUSCLE program [49]. Phylogenetic tree was constructed using the UGENE program using the aforementioned models and visualized using Ugene’s tree view [50].

### 3.8. UPLC-QTOF-SIE Detection of ***5**–**14*** in CMB-M0339 Extract

The EtOAc extract of a CMB-M0339 D400 agar culture was dissolved in MeOH and subjected to UPLC-QTOF analysis with single ion extraction (SIE) analysis (Figure S70).

### 3.9. X-ray Crystallography

Crystals of **5** were obtained by slow evaporation from 50% DCM/Hexane in the cold room (−4 °C). Crystallographic data (Cu Kα, 2θ_max_ = 125°) for **5** were collected on an Oxford Diffraction Gemini S Ultra CCD diffractometer with the crystal cooled to 190 K with an Oxford Cryosystems Desktop Cooler. Data reduction and empirical absorption corrections were carried out with the CrysAlisPro program. The structure was solved with SHELXT and refined with SHELXL [51]. The thermal ellipsoid diagrams were generated with Mercury [52]. All calculations were carried out within the WinGX graphical user interface [53]. The disordered water molecules in the structure were modelled with SQUEEZE implemented in PLATON [54]. The crystal data for **5** in CIF format were deposited in the CCDC database (2206901) (Table S13).

### 3.10. Antifungal Assay

The fungus *Candida albicans* ATCC 10231 was streaked onto a LB (Luria–Bertani) agar plate and was incubated at 37 °C for 48 h, after which a colony was transferred to fresh LB broth (15 mL) and the cell density was adjusted to 10^4^–10^5^ CFU/mL. Test compounds were dissolved in DMSO and diluted with H_2_O to prepare 600 µM stock solutions (20% DMSO), which were serially diluted with 20% DMSO to provide concentrations from 600 µM to 0.2 µM in 20% DMSO. An aliquot (10 µL) of each dilution was transferred to a 96-well microtiter plate and freshly prepared fungal broth (190 µL) was added to prepare final concentrations of 30–0.01 µM in 1% DMSO. The plates were incubated at 27 °C for 48 h and the optical density of each well was measured spectrophotometrically at 600 nm using POLARstar Omega plate (BMG LABTECH, Offenburg, Germany). Amphotericin B was used as the positive control (40 µg/mL in 10% DMSO). The IC_50_ value was calculated as the concentration of the compound or antibiotic required for 50% inhibition of the bacterial cells using Prism 7.0 (GraphPad Software Inc., La Jolla, CA, USA). See Figure S80.

### 3.11. Antibacterial Assay

The bacterium to be tested was streaked onto an LB agar plate and was incubated at 37 °C for 24 h, after which a colony was transferred to fresh LB broth (15 mL) and the cell density was adjusted to 10^4–^10^5^ CFU/mL. Test compounds were dissolved in DMSO and diluted with H_2_O to give 600 µM stock solutions (20% DMSO), which were serially diluted with 20% DMSO to prepare concentrations from 600 µM to 0.2 µM in 20% DMSO. An aliquot (10 µL) of each dilution was transferred to a 96-well microtiter plate and freshly prepared microbial broth (190 µL) was added to provide final concentrations of 30–0.01 µM in 1% DMSO. The plates were incubated at 37 °C for 24 h and the optical density of each well was measured spectrophotometrically at 600 nm using POLARstar Omega plate (BMG LABTECH, Offenburg, Germany). Each test compound was screened against the Gram-negative bacterium *Escherichia coli* ATCC 11775 and the Gram-positive bacteria *Staphylococcus aureus* ATCC 25923 and *Bacillus subtilis* ATCC 6633. Rifampicin was used as the positive control (40 µg/mL in 10% DMSO) for Gram-positive bacteria and a mixture of rifampicin and ampicillin was used as the positive control for Gram-negative bacteria. The IC_50_ value was calculated as the concentration of the compound or antibiotic required for 50% inhibition of the bacterial cells using Prism 7.0 (GraphPad Software Inc., La Jolla, CA, USA). See Figure S80.

### 3.12. Cytotoxicity Assays

Human colorectal (SW620) and lung carcinoma (NCI-H460) cells were seeded evenly in a 96-well micro-plate (2000 cells/well in 180 μL of RPMI 1640 medium (Roswell Park Memorial Institute medium) supplemented with 10% FBS (Fetal Bovine Serum)) and the plate was incubated for 18 h (37 °C; 5% CO_2_) to allow cells to attach. Test compounds were dissolved in 5% DMSO (*v*/*v*) and dilutions were generated from 300 μM to 300 nM. Aliquots (20 μL) of each dilution (or 5% aqueous DMSO for negative control and 5% aqueous SDS for positive control) were added to the plate in duplicate. After 68 h of incubation (37 °C; 5% CO_2_), a solution of 3-(4,5-dimethylthiazol-2-yl)-2,5-diphenyltetrazolium bromide (MTT; Sigma, USA) in PBS (Phosphate Buffered Saline) was added to each well to a final concentration of 0.4 mg/mL and plates were incubated for a further 4 h (37 °C; 5% CO_2_) after which the medium was carefully aspirated and precipitated formazan crystals were dissolved in DMSO (100 μL/well). The absorbance of each well at 580 nm was measured with a PowerWave XS Microplate Reader from Bio-Tek Instruments Inc. (Vinooski, VT) and IC_50_ values were calculated as the concentration of the compound required for 50% inhibition of the cancer cells using Prism 5.0 from GraphPad Software Inc. (La Jolla, CA, USA). See Figure S81.

## Figures and Tables

**Table 1:** 1D NMR (methanol-*d*_4_) data for noonindoles A–C (**5**–**7**) ^#^.

Pos.	(5) *δ*_H,_ mult. (*J* in Hz)	*δ*_C_	(6) *δ*_H,_ mult. (*J* in Hz)	*δ*_C_	(7) *δ*_H,_ mult. (*J* in Hz)	*δ*_C_
2	-	152.4	-	152.3	-	152.2
3	-	52.0	-	51.9	-	51.8
4	-	46.4	-	46.2	-	46.2
5	*a* 2.87, ddd (*13.6*, *13.6*, *5.2*) *b* 1.81, br dd (*13.6*, *5.2*)	28.1	*a* 2.88, ddd (*13.5*, *13.5*, *5.1*) *b* 1.83, br dd (*13.5*, *5.1*)	28.0	*a* 2.87, ddd (*13.8*, *13.8*, *5.4*) *b* 1.84, br dd (*13.8*, *5.4*)	27.9
6	*a* 2.38, m *b* 1.91, m	30.6	*a* 2.39, m *b* 1.93, m	30.5	*a* 2.39, m *b* 1.93, m	30.5
7	4.91, m	74.6	4.90, m	74.8	4.92, br t (*8.5*)	74.8
9	3.68, d (*1.9*)	84.0	3.72, d (*1.5*)	83.9	3.71, br s	84.0
10	-	198.5	-	198.1	-	197.9
11	5.62, d (*1.9*)	122.4	5.57, d (*1.5*)	122.0	5.57, d (*1.9*)	121.8
12	-	165.9	-	165.8	-	165.0
13	-	80.0	-	80.0	-	80.1
14	5.53, dd (*10.2*, *5.3*)	77.9	5.50, dd (*10.4*, *5.0*)	77.3	5.51, dd (*10.4*, *5.1*)	77.5
15	*a* 2.24, dd (*11.1*, *10.2*) *b* 2.21, m	28.5	*a* 2.25, dd (*13.0*, *10.4*) *b* 2.17, m	28.3	*a* 2.25, dd (*13.2*, *10.4*) *b* 2.20, m	28.1
16	2.92, m	47.4	2.98, m	47.5	2.97, m	47.5
17	*a* 2.73, dd (*13.0*, *6.2*) *b* 2.47 ^a^, m	27.6	*a* 2.74, dd (*13.1*, *6.3*) *b* 2.47, dd (*13.1*, *11.1*)	27.6	*a* 2.74, dd (*13.0*, *6.3*) *b* 2.47, dd (*13.0*, *10.8*)	27.6
18	-	117.4	-	117.3	-	117.2
19	-	126.2	-	126.0	-	126.1
20	7.31, d (*7.5*)	119.0	7.30, d (*7.9*)	118.8	7.30, d (*7.8*)	118.9
21	6.94, ddd (*7.5*, *7.5*, *1.1*)	120.1	6.93, br dd (*7.9*, *7.7*)	119.9	6.93, ddd (*7.8*, *7.8*, *1.1*)	119.9
22	6.98, ddd (*7.5*, *7.5*, *1.1*)	121.1	6.97, ddd (*7.9*, *7.9*, *0.9*)	121.1	6.98, ddd (*7.8*, *7.8*, *1.1*)	121.1
23	7.27, d (*7.5*)	112.7	7.28, d (*7.9*)	112.7	7.28, d (*7.8*)	112.7
24	-	141.8	-	141.9	-	141.9
25	-	73.3	-	73.0	-	73.0
26	1.28, s	25.4	1.28, s	25.3	1.30, s	25.5
27	1.26, s	26.2	1.26, s	26.4	1.27, s	25.8
1′	-	167.4	-	168.9	-	168.9
2′	3.86, d (*5.5*)	74.8	3.90, br s	68.7	4.02, m	67.5
3′	2.47 ^a^, m -	28.4 -	2.31, m -	30.7 -	*a* 1.90, m *b* 1.69, m	37.4 37.4
4′	1.05 ^b^	19.4	1.09, br d (*5.3*)	18.7	1.56, m	26.2
5′	1.03, d (*7.1*)	17.0	0.99, d (*7.0*)	17.7	0.97, d (*6.4*)	23.4
6′	-	-	-	-	0.91, d (*6.4*)	21.4
3-Me	1.37, s	16.7	1.37, s	16.6	1.38, s	16.6
4-Me	1.04 ^b^	19.4	1.10, s	19.5	1.09, br s	19.4
NHMe	-	-	2.72, s	33.8	-	-
NMe_2_	2.90, s	43.0	-	-	2.93, s	42.1

^#^ Data acquired on the TFA salts. ^a,b^ Resonances with the same superscript overlap and assignments can be interchanged.

**Table 2:** 1D NMR (methanol-*d*_4_) data for noonindoles D–F (**8**–**10**).

Pos.	(8) *δ*_H,_ mult. (*J* in Hz)	*δ*_C_	(9) *δ*_H,_ mult. (*J* in Hz)	*δ*_C_	(10) *δ*_H,_ mult. (*J* in Hz)	*δ*_C_
2	-	151.1	-	152.5	-	152.9
3	-	50.5	-	51.9	-	51.8
4	-	44.6	-	46.2	-	44.7
5	*a* 2.87 ^a^, m *b* 1.80, m	28.0	*a* 3.28 * b 2.36, d (*6.3*)	33.0	*a* 2.80, ddd (*13.5*, *13.5*, *5.1*) *b* 1.79, dd (*13.5*, *5.1*)	28.1
6	*a* 2.37, m *b* 1.91, m	30.4	5.74, m	113.5	*a* 2.33, m *b* 1.90, m	30.2
7	4.90, m	74.6	-	146.4	4.88, m	74.7
9	3.71, d (*1.9*)	83.7	4.09, s	87.6	3.78, d (*1.5*)	84.0
10	-	nd	-	196.8	-	200.0
11	5.58, d (*1.9*)	121.7	5.71, br s	119.5	6.01, br s	122.9
12	-	nd	-	nd	-	167.5
13	-	78.9	-	77.8	-	80.8
14	5.37, dd (*10.6*, *5.1*)	74.4	5.41, dd (*9.8*, *5.8*)	75.5	4.16, dd (*10.5*, *4.9*)	70.3
15	*a* 2.16, m *b* 2.04, ddd (*7.1*, *5.1*, *2.3*)	28.4	2.18, m	30.0	*a* 2.08, dd (*13.3*, *10.5*) *b* 1.93, m	31.7
16	2.95, m	47.0	2.90, m	47.5	2.90, m	48.0
17	*a* 2.72, dd (*13.1*, *6.4*) *b* 2.45, dd (*13.1*, *10.7*)	27.5	*a* 2.72, dd (*13.0*, *6.5*) *b* 2.44, dd (*13.0*, *10.8*)	28.0	*a* 2.68, dd (*13.0*, *6.1*) *b* 2.42, dd (*13.0*, *10.0*)	27.8
18	-	116.8	-	117.4	-	117.4
19	-	124.7	-	126.2	-	126.2
20	7.30, d (*7.7*)	118.6	7.30, d (*7.2*)	119.1	7.29, d (*7.6*)	118.8
21	6.92, ddd (*7.7*, *7.3*, *0.9*)	119.6	6.93, m	120.0	6.92, br dd (*7.6*, *7.2*)	119.8
22	6.97, ddd (*8.1*, *7.3*, *0.9*)	120.7	6.97, m	121.2	6.95, br dd (*7.4*, *7.2*)	120.9
23	7.27, d (*8.1*)	112.4	7.27, d (*7.5*)	112.8	7.26, d (*7.4*)	112.7
24	-	140.4	-	142.1	-	141.8
25	-	71.6	-	75.6	-	73.2
26	1.28, s	25.1	1.33, s	27.1 ^a^	1.29, s	25.2
27	1.26, s	25.9	1.26, s	27.1 ^a^	1.27, s	26.2
1′	-	nd	-	nd	-	-
2′	2.87 ^a^, m	72.9	2.86, d (*9.4*)	76.0	-	-
3′	*a* 1.77, m *b* 1.69, m	23.8	2.11, m	28.7	- -	- -
4′	0.91, t (*7.4*)	10.1	0.97, t (*7.4*)	19.9	-	-
5′	-	-	0.97, t (*7.4*)	19.9	-	-
3-Me	1.37, s	16.3	1.40, s	16.9	1.32, s	16.5
4-Me	1.08, s	19.4	1.14, s	21.0	1.04, s	19.6
NMe_2_	2.26, s	43.4	2.34, s	42.4	-	-

nd: Not detected. ^a^ Resonances with the same superscript within a column are overlapping. * Obscured by residual solvent resonance. ^#^ Data for **8**–**9** acquired on the free bases (not TFA salts).

**Table 3:** Comparison of **5**–**9** and minor co-metabolites **i**–**viii**.

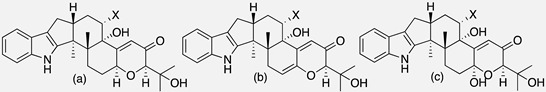			
X (ester)	(a)	(b)	(c)
*N*,*N*-dimethyl-valine	(**5**)	(**9**)	(**viii**)
*N*-methyl-valine	(**6**)	-	-
*N*,*N*-dimethyl-leucine	(**7**)	(**vii**)	(**vi**)
*N*,*N*-dimethyl-homoalanine	(**8**)	-	-
*N*,*N*-dimethyl-alanine ^A^	(**i**)	-	-
*N*,*N*-dimethyl-serine ^B^	(**ii**)	-	-
*N*,*N*-dimethyl-threonine	(**iii**)	-	-
*N*-methyl-proline ^C^	(**iv**)	-	-
*N*,*N*-dimethyl-isoleucine ^D^	(**v**)	-	-

(a,b,c) Proposed hexacyclic scaffolds. Possible alternate isomers: ^A^ *N*-methyl-homoalanine, valine; ^B^ *N*-methyl-threonine; ^C^ pipecolic acid; ^D^ *N*,*N*-dimethyl-*allo*-isoleucine.

## Data Availability

Raw NMR data is available at https://npmrd-project.org/ (access on 26 September 2022).

## Supplementary Materials

The following supporting information can be downloaded at: https://www.mdpi.com/article/10.3390/md20110698/s1, Figure S1: ITS gene sequence of CMB-M0339, Figure S2: NCBI-BLAST search of 18S rRNA sequence of CMB-M0339, Figure S3: Blast search for CMB-M0339, Figure S4: Phylogenetic tree, Figure S5: CMB-M0339 cultivated on SD agar, Figure S6: UPLC-DAD chromatograms of crude extract of CMB-M0339, Figure S7: CMB-M0339 cultivated under MATRIX conditions, Figure S8: UPLC-DAD chromatograms of MATRIX extracts, Figure S9: Isolation scheme for **5–14**, Figures S10–S69: Annotated 1D and 2D NMR spectra and HRMS spectra for **5–14**, Figure S70–S71: Single ion extraction of fresh crude extract from CMB-M0339 showing the presence of **5–14** and **1**, Figures S72–S79: MS/MS fragmentation pattern of **5–9** and **i**-**v**, Figures S80–S81: Antimicrobial and cytotoxic activities of **5–14**. Table S1: Composition of media used for cultivation profiling, Tables S2–S11: 1D and 2D NMR data for **5–14**, Table S12: Predicted molecular formulae generated for **5–9** and **i**-**viii** observed in GNPS cluster, Table S13: Bond lengths and angles for X-ray crystal structure of **5**. Refs. [18,24] are cited in the Supplementary Materials.
