# Supplementary material for: Noonindoles A–F: Rare Indole Diterpene Amino Acid Conjugates from a Marine-Derived Fungus, Aspergillus noonimiae CMB-M0339
Source: Mar Drugs. 2022 Nov 7;20(11):698. doi: 10.3390/md20110698 (PMC9694122; doi:10.3390/md20110698)
Supplement: Supplementary file 1 [file marinedrugs-20-00698-s001.zip › marinedrugs-2008590-supplementary.pdf]

Supporting Information

Noonindoles A–F: Rare indole diterpene amino acid conjugates from a marine-derived fungus, *Aspergillus noonimiae* CMB-M0339

Sarani Kankanamge[1], Zeinab G. Khalil[2], Paul V. Bernhardt[3] and Robert J. Capon[2,*]

[1]Institute for Molecular Bioscience, The University of Queensland, Brisbane, QLD 4072, Australia

[3]School of Chemistry and Molecular Bioscience, The University of Queensland, Brisbane, QLD 4072, Australia

*Corresponding author: r.capon@uq.edu.au

List of contents

1 Phylogenetic tree 8
2 Chemical investigation of CMB-M0339 9
3 MATRIX cultivation profiling of CMB-M0339 9
4 Noonindole A (**5**) 13
5 Noonindole B (**6**) 18
6 Noonindole C (**7**) 23
7 Noonindole D (**8**) 28
8 Noonindole E (**9**) 33
9 Noonindole F (**10**) 38
10 Paspaline (**11**) 43
11 Paspaline B (**12**) 47
12 12-demethylpaspaline-12-carboxylic acid (**13**) 51
13 Emindole SB (**14**) 55
14 MS/MS fragmentation patterns of **5** - **9** and **i-v** 60
15 Crystal data and structure refinement for noonindole A (**5**). 68
16 Biological activity of **5-14** 72
17 References 72

List of figures

Figure S1. ITS gene sequence of CMB-M0339........6
Figure S2. NCBI-BLAST search of 18S rRNA sequence of CMB-M0339........6
Figure S3. Blast search (closet match) for CMB-M0339........7
Figure S4. Phylogenetic tree........8
Figure S5. CMB-M0339 cultivated on SD agar........8
Figure S6. UPLD-DAD chromatograms of crude extract of CMB-M0339........9
Figure S7. CMB-M0339 cultivated under MATRIX conditions........9
Figure S8. UPLC-DAD chromatograms of MATRIX extracts of CMB-M0339........10
Figure S9. Isolation scheme for **5-14** from CMB-M0339........12
Figure S10. ^{1}H NMR (600 MHz, methanol-d_4) spectrum of noonindole A (**5**)........14
Figure S11. ^{13}C NMR (150 MHz, methanol-d_4) spectrum of noonindole A (**5**)........14
Figure S12. HSQC (methanol-d_4) spectrum of noonindole A (**5**)........15
Figure S13. COSY (methanol-d_4) spectrum of noonindole A (**5**)........15
Figure S14. HMBC (methanol-d_4) spectrum of noonindole A (**5**)........16
Figure S15. ROESY (methanol-d_4) spectrum of noonindole A (**5**)........16
Figure S16. HRMS spectrum of noonindole A (**5**)........17
Figure S17.^{1}H NMR (600 MHz, methanol-d_4) spectrum of noonindole B (**6**)........19
Figure S18. ^{13}C NMR (150 MHz, methanol-d_4) spectrum of noonindole B (**6**)........19
Figure S19. HSQC (methanol-d_4) spectrum of noonindole B (**6**)........20
Figure S20. COSY (methanol-d_4) spectrum of noonindole B (**6**)........20
Figure S21. HMBC (methanol-d_4) spectrum of noonindole B (**6**)........21
Figure S22. ROESY (methanol-d_4) spectrum of noonindole B (**6**)........21
Figure S23. HRMS spectrum of noonindole B (**6**)........22
Figure S24. ^{1}H NMR (600 MHz, methanol-d_4) spectrum of noonindole C (**7**)........24
Figure S25. ^{13}C NMR (150 MHz, methanol-d_4) spectrum of noonindole C (**7**)........24
Figure S26. HSQC (methanol-d_4) spectrum of noonindole C (**7**)........25
Figure S27. COSY (methanol-d_4) spectrum of noonindole C (**7**)........25
Figure S28. HMBC (methanol-d_4) spectrum of noonindole C (**7**)........26
Figure S29. ROESY (methanol-d_4) spectrum of noonindole C (**7**)........26
Figure S30. HRMS spectrum of noonindole C (**7**)........27
Figure S31. ^{1}H NMR (methanol-d_4) spectrum of noonindole D (**8**)........29
Figure S32. HSQC (methanol-d_4) spectrum of noonindole D (**8**)........30
Figure S33. COSY (methanol-d_4) spectrum of noonindole D (**8**)........30
Figure S34. HMBC (methanol-d_4) spectrum of noonindole D (**8**)........31
Figure S35. ROESY (methanol-d_4) spectrum of noonindole D (**8**)........31
Figure S36. HRMS spectrum of noonindole D (**8**)........32
Figure S37. ^{1}H NMR (methanol-d_4) spectrum of noonindole E (**9**)........34
Figure S38. HSQC (methanol-d_4) spectrum of noonindole E (**9**)........35
Figure S39. COSY (methanol-d_4) spectrum of noonindole E (**9**)........35
Figure S40. HMBC (methanol-d_4) spectrum of noonindole E (**9**)........36
Figure S41. ROESY (methanol-d_4) spectrum of noonindole E (**9**)........36
Figure S42. HRMS spectrum of noonindole E (**9**)........37
Figure S43. ^{1}H NMR (methanol-d_4) spectrum of noonindole F (**10**)........39
Figure S44. ^{13}C NMR (methanol-d_4) spectrum of noonindole F (**10**)........39
Figure S45. HSQC (methanol-d_4) spectrum of compound noonindole F (**10**)........40
Figure S46. COSY (methanol-d_4) spectrum of compound noonindole F (**10**)........40
Figure S47. HMBC (methanol-d_4) spectrum of compound noonindole F (**10**)........41
Figure S48. ROESY (methanol-d_4) spectrum of compound noonindole F (**10**)........41
Figure S49. HRMS spectrum of noonindole F (**10**)........42

Figure S50.^{1}H NMR (DMSO-d_6) spectrum of paspaline (**11**)....44
Figure S51. HSQC (DMSO-d_6) spectrum of paspaline (**11**)....44
Figure S52. COSY (DMSO-d_6) spectrum of paspaline (**11**)....45
Figure S53. HMBC (DMSO-d_6) spectrum of paspaline (**11**)....45
Figure S54. HRMS spectrum of paspaline (**11**)....46
Figure S55. ^{1}H NMR ($CDCl_3$) spectrum of paspaline B (**12**)....48
Figure S56. HSQC ($CDCl_3$) spectrum of paspaline B (**12**)....48
Figure S57. COSY ($CDCl_3$) spectrum of paspaline B (**12**)....49
Figure S58. HMBC ($CDCl_3$) spectrum of paspaline B (**12**)....49
Figure S59. HRMS spectrum of paspaline B (**12**)....50
Figure S60. ^{1}H NMR (DMSO-d_6) spectrum of 12-demethylpaspaline-12-carboxylic acid (**13**)....52
Figure S61. HSQC (DMSO-d_6) spectrum of 12-demethylpaspaline-12-carboxylic acid (**13**) 52
Figure S62. COSY (DMSO-d_6) spectrum of 12-demethylpaspaline-12-carboxylic acid (**13**) 53
Figure S63. HMBC (DMSO-d_6) spectrum of 12-demethylpaspaline-12-carboxylic acid (**13**)....53
Figure S64. HRMS spectrum of 12-demethylpaspaline-12-carboxylic acid (**13**)....54
Figure S65. ^{1}H NMR (DMSO-d_6) spectrum of emindole SB (**14**)....56
Figure S66. HSQC (DMSO-d_6) spectrum of emindole SB (**14**)....56
Figure S67. COSY (DMSO-d_6) spectrum of emindole SB (**14**)....57
Figure S68. HMBC (DMSO-d_6) spectrum of emindole SB (**14**)....57
Figure S69. HRMS spectrum of emindole SB (**14**)....58
Figure S70. Single ion extraction of CMB-M0339 fresh crude extract, showing the presence of isolated metabolites....59
Figure S71. Single ion extraction of crude extract of CMB-M0339, showing the presence of paxilline (**1**)....59
Figure S72. MS/MS fragmentation pattern of (**5**)....60
Figure S73. MS/MS fragmentation pattern of (**6**)/(**8**)....61
Figure S74. MS/MS fragmentation pattern of (**7**)/**v**....62
Figure S75. MS/MS fragmentation pattern of (**9**)....63
Figure S76. MS/MS fragmentation pattern of **i**....64
Figure S77. MS/MS fragmentation pattern of **ii**....65
Figure S78. MS/MS fragmentation pattern of **iii**....66
Figure S79. MS/MS fragmentation pattern of **iv**....67
Figure S80. Antimicrobial activity of metabolites **5–14**....72
Figure S81. Cytotoxicity of metabolites **5–14**....72

List of tables

Table S1. Composition of media used for cultivation profiling 11
Table S2. 1D and 2D NMR (methanol-d_4, 600 MHz) data for noonindole A (**5**)....................13
Table S3. 1D and 2D NMR (methanol-d_4, 600 MHz) data for noonindole B (**6**)....................18
Table S4. 1D and 2D NMR (in methanol-d_4, 600 MHz) data for noonindole C (**7**)................23
Table S5. 1D and 2D NMR (methanol-d_4, 600 MHz) data for noonindole D (**8**)....................28
Table S6. 1D and 2D NMR (methanol-d_4, 600 MHz) data for noonindole E (**9**)....................33
Table S7. 1D and 2D NMR (in methanol-d_4, 600 MHz) data for noonindole F (**10**) 38
Table S8. 1D and 2D NMR (DMSO-d_6, 600 MHz) data of paspaline (**11**) 43
Table S9. 1D and 2D NMR ($CDCl_3$, 600 MHz) data of paspaline B (**12**)..............................47
Table S10. 1D and 2D NMR (DMSO-d_6, 600 MHz) data of 12-demethylpaspaline-12-carboxylic acid (**13**) 51
Table S11. 1D and 2D NMR (DMSO-d_6, 600 MHz) data of emindole SB (**14**)......................55
Table S12. Molecular formulae generated for noonindoles A-E (**5** - **9**) and predicted molecular formulae for minor metabolites (**i**) – (**viii**) observed in GNPS cluster 68
Table S13. Bond lengths [Å] and angles [°] for X-ray crystal structure of noonindole A (**5**).69

CGGCACCCTCGCGGTGCCAACCTCCCATCCTTGTCTATTGTTACCGTCGTTGCTTCGGCGGG
CCCGTTCCTCCTCCCCCGGGGGGAGGGCCGTCGGGGGGCATTCGCCCCCGGGCGAGCGCCCG
CCGGAGACCCCAACACGAACTCTGAGTGAAAGACTGTCGTCTGAGTGGGCTTTTTGAATCAG
TTAAAACTTTCAACAACGGATCTCTTGGTTCCGGCATCGATGAAGAACGCAGCGAACTGCGA
TAAGTAATGTGAATTGCAGAATTCAGTGAATCATCGAGTCTTTGAACGCATATTGCGCCCCC
TGGTATTCCGGGGGGCATGCCTGTCCGAGCGTCATTGCTACCCTCAAGCACGGCTTGTGTGT
TGGGTCGGCGTCCCCGGGGAGTCCCCGGGGACGGGCCCGAAAGGCAGCGGCGGCACCGCGTC
CTGGTCCTCGAGCGTATGGGGCTCTGTCACCCGCTCTGAGGGGCCGGCCGGCGCCTTTGGCC
AACCTGTTTATGGGCCCTTCCGGGGGACCGAAACACCATTTTTTTCTCAGGTTGACCTCGGA
TCAGGTAGGGATACCCGCTGAACTTAAGCATATCAATAAGGCGGAGGA (**606 bp**)

Figure S1. ITS gene sequence of CMB-M0339

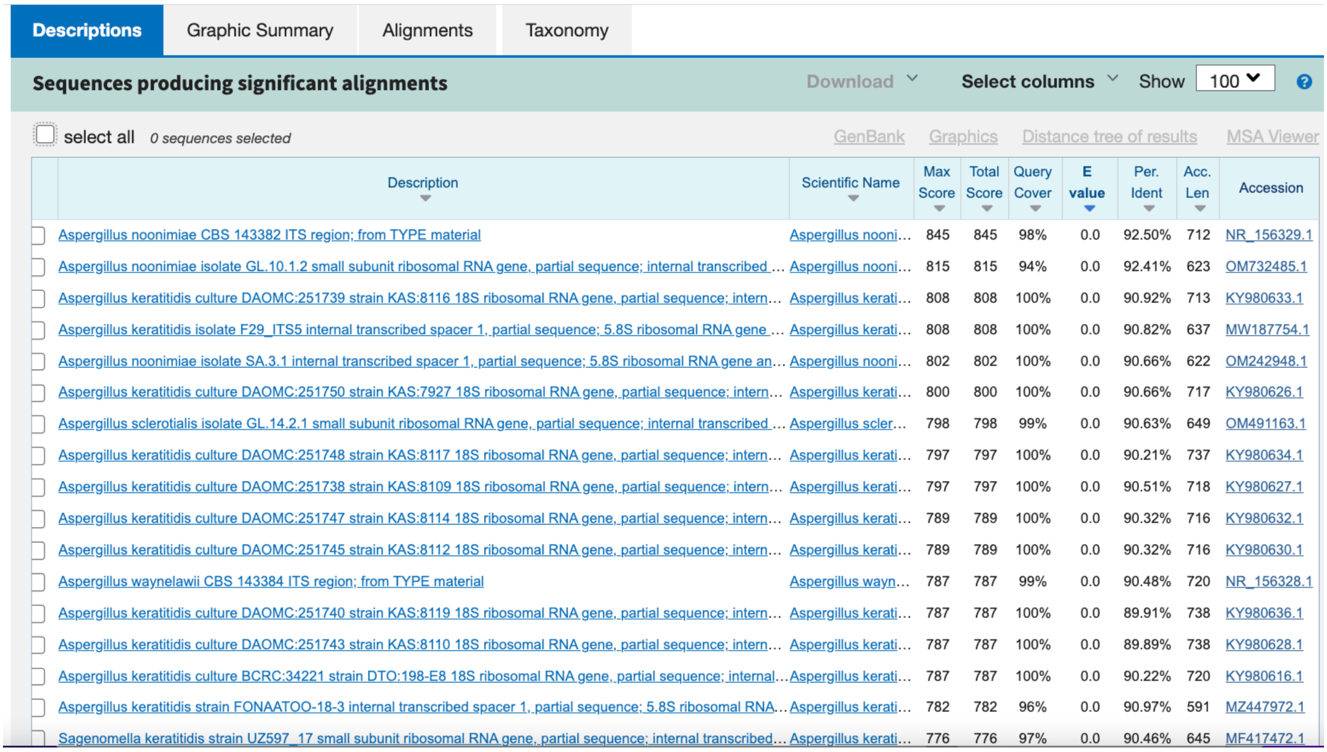
Descriptions | Graphic Summary | Alignments | Taxonomy

Sequences producing significant alignments

Download | Select columns | Show 100

select all 0 sequences selected | GenBank | Graphics | Distance tree of results | MSA Viewer

Description	Scientific Name	Max Score	Total Score	Query Cover	E value	Per. Ident	Acc. Len	Accession
Aspergillus noonimiae CBS 143382 ITS region; from TYPE material	Aspergillus nooni…	845	845	98%	0.0	92.50%	712	NR_156329.1
Aspergillus noonimiae isolate GL.10.1.2 small subunit ribosomal RNA gene, partial sequence; internal transcribed …	Aspergillus nooni…	815	815	94%	0.0	92.41%	623	OM732485.1
Aspergillus keratitidis culture DAOMC:251739 strain KAS:8116 18S ribosomal RNA gene, partial sequence; intern…	Aspergillus kerati…	808	808	100%	0.0	90.92%	713	KY980633.1
Aspergillus keratitidis isolate F29_ITS5 internal transcribed spacer 1, partial sequence; 5.8S ribosomal RNA gene …	Aspergillus kerati…	808	808	100%	0.0	90.82%	637	MW187754.1
Aspergillus noonimiae isolate SA.3.1 internal transcribed spacer 1, partial sequence; 5.8S ribosomal RNA gene an…	Aspergillus nooni…	802	802	100%	0.0	90.66%	622	OM242948.1
Aspergillus keratitidis culture DAOMC:251750 strain KAS:7927 18S ribosomal RNA gene, partial sequence; intern…	Aspergillus kerati…	800	800	100%	0.0	90.66%	717	KY980626.1
Aspergillus sclerotialis isolate GL.14.2.1 small subunit ribosomal RNA gene, partial sequence; internal transcribed …	Aspergillus scler…	798	798	99%	0.0	90.63%	649	OM491163.1
Aspergillus keratitidis culture DAOMC:251748 strain KAS:8117 18S ribosomal RNA gene, partial sequence; intern…	Aspergillus kerati…	797	797	100%	0.0	90.21%	737	KY980634.1
Aspergillus keratitidis culture DAOMC:251738 strain KAS:8109 18S ribosomal RNA gene, partial sequence; intern…	Aspergillus kerati…	797	797	100%	0.0	90.51%	718	KY980627.1
Aspergillus keratitidis culture DAOMC:251747 strain KAS:8114 18S ribosomal RNA gene, partial sequence; intern…	Aspergillus kerati…	789	789	100%	0.0	90.32%	716	KY980632.1
Aspergillus keratitidis culture DAOMC:251745 strain KAS:8112 18S ribosomal RNA gene, partial sequence; intern…	Aspergillus kerati…	789	789	100%	0.0	90.32%	716	KY980630.1
Aspergillus waynelawii CBS 143384 ITS region; from TYPE material	Aspergillus wayn…	787	787	99%	0.0	90.48%	720	NR_156328.1
Aspergillus keratitidis culture DAOMC:251740 strain KAS:8119 18S ribosomal RNA gene, partial sequence; intern…	Aspergillus kerati…	787	787	100%	0.0	89.91%	738	KY980636.1
Aspergillus keratitidis culture DAOMC:251743 strain KAS:8110 18S ribosomal RNA gene, partial sequence; intern…	Aspergillus kerati…	787	787	100%	0.0	89.89%	738	KY980628.1
Aspergillus keratitidis culture BCRC:34221 strain DTO:198-E8 18S ribosomal RNA gene, partial sequence; internal…	Aspergillus kerati…	787	787	100%	0.0	90.22%	720	KY980616.1
Aspergillus keratitidis strain FONAATOO-18-3 internal transcribed spacer 1, partial sequence; 5.8S ribosomal RNA…	Aspergillus kerati…	782	782	96%	0.0	90.97%	591	MZ447972.1
Sagenomella keratitidis strain UZ597_17 small subunit ribosomal RNA gene, partial sequence; internal transcribed…	Aspergillus kerati…	776	776	97%	0.0	90.46%	645	MF417472.1

Figure S2. NCBI-BLAST search of 18S rRNA sequence of CMB-M0339

Aspergillus noonimiae CBS 143382 ITS region; from TYPE material

Sequence ID: NR_156329.1 Length: 712 Number of Matches: 1

See 1 more title(s) See all Identical Proteins(IPG)

Range 1: 52 to 642 GenBank Graphics ▼ Next Match ▲ Previous Match

Score	Expect	Identities	Gaps	Strand
845 bits(457)	0.0	555/600(93%)	15/600(2%)	Plus/Plus

```
Query  13   GGTGCCAACCTCCCATCCTTGTCTATTGTTACCGTCGTTGCTTCGGCGGGCCCGTTCCTC  72
            |||||||||||||||||| ||||||||| |||| |||||||||||||||||||||| |||
Sbjct  52   GGTGCCAACCTCCCATCCGTGTCTATTG-TACCTTCGTTGCTTCGGCGGGCCCGTTTCTC  110

Query  73   CT---CCCCCGGG-GGGAGGGCCGTCGGGGGGCATTCGCCCCCGGGCGAGCGCCCGCCGG  128
            ||   ||||||||  |||||||||||||||||||   ||||||||||| | |||||||||
Sbjct  111  CTTCCCCCCCGGGAAGGAGGGCCGTCGGGGGGCAGCTGCCCCCGGGCGTGTGCCCGCCGG  170

Query  129  AGACCCCAACACGAACTCTGAGTGAAAGACTGTCGTCTGAGTGGGCTTTT-TGAATCAGT  187
            ||||||||||||||||||||  ||||||||||||||||||||||| |||| | ||||| |
Sbjct  171  AGACCCCAACACGAACTCTGTCTGAAAGACTGTCGTCTGAGTGGGTTTTTATAAATCATT  230

Query  188  TAAAACTTTCAACAACGGATCTCTTGGTTCCGGCATCGATGAAGAACGCAGCGAACTGCG  247
            ||||||||||||||||||||||||||||||||||||||||||||||||||||||||||||
Sbjct  231  TAAAACTTTCAACAACGGATCTCTTGGTTCCGGCATCGATGAAGAACGCAGCGAACTGCG  290

Query  248  ATAAGTAATGTGAATTGCAGAATTCAGTGAATCATCGAGTCTTTGAACGCATATTGCGCC  307
            ||||||||||||||||||||||||||||||||||||||||||||||||||||||||||||
Sbjct  291  ATAAGTAATGTGAATTGCAGAATTCAGTGAATCATCGAGTCTTTGAACGCATATTGCGCC  350

Query  308  CCCTGGTATTCCGGGGGGCATGCCTGTCCGAGCGTCATTGCTACCCTCAAGCACGGCTTG  367
            ||||||||||||||||||||||||||||||||||||||||||||||||||||||||||||
Sbjct  351  CCCTGGTATTCCGGGGGGCATGCCTGTCCGAGCGTCATTGCTACCCTCAAGCACGGCTTG  410

Query  368  TGTGTTGGGTCGGCGTCCCCGGGGAGT-CCCCGGGGACGGCCCGAAAGGCAGCGGCGGC  426
            ||||||||||||||||||| ||||    ||||||||||||||||||||||||||||||||
Sbjct  411  TGTGTTGGGTCGGCGTCCCTGGGGCTCCCCCCGGGGACGGCCCGAAAGGCAGCGGCGGC  470

Query  427  ACCGCGTCCTGGTCCTCGAGCGTATGGGGCTCTGTCACCCGCTCTGAGGGGCCGGCCGGC  486
            |||||||||||||||||||||||||||||||||||||||||||| |||||||||||||||
Sbjct  471  ACCGCGTCCTGGTCCTCGAGCGTATGGGGCTCTGTCACCCGCTCGGAGGGGCCGGCCGGC  530

Query  487  GCCTTTGGCCAACCTGTTTATGGGCCCTTCCGGGGGACCGAAACACCAtttttttCTCAG  546
            |||||||||||   | ||| ||| |  ||| |||  | ||||| ||  ||  ||||| ||
Sbjct  531  GCCTTTGGCCATTATTTTTCTGGTC--TTCTGGG--ATCGAAAAAC--TTC-TTTCTTAG  583

Query  547  GTTGACCTCGGATCAGGTAGGGATACCCGCTGAACTTAAGCATATCAATAAGGCGGAGGA  606
            |||||||||||||||||||||||||||||||||||||||||||||||||||| |||||||
Sbjct  584  GTTGACCTCGGATCAGGTAGGGATACCCGCTGAACTTAAGCATATCAATAAG-CGGAGGA  642
```

Aspergillus noonimiae CBS 143382 ITS region; from TYPE material

NCBI Reference Sequence: NR_156329.1

FASTA Graphics

Go to:

```
LOCUS       NR_156329                712 bp    DNA     linear   PLN 27-JUN-2018
DEFINITION  Aspergillus noonimiae CBS 143382 ITS region; from TYPE material.
ACCESSION   NR_156329
VERSION     NR_156329.1
DBLINK      BioProject: PRJNA177353
KEYWORDS    RefSeq.
SOURCE      Aspergillus noonimiae
  ORGANISM  Aspergillus noonimiae
            Eukaryota; Fungi; Dikarya; Ascomycota; Pezizomycotina;
            Eurotiomycetes; Eurotiomycetidae; Eurotiales; Aspergillaceae;
            Aspergillus; Aspergillus subgen. Polypaecilum.
REFERENCE   1  (bases 1 to 712)
  AUTHORS   Tanney,J.B., Visagie,C.M., Yilmaz,N. and Seifert,K.A.
  TITLE     Aspergillus subgenus Polypaecilum from the built environment
  JOURNAL   Stud. Mycol. 88, 237-267 (2018)
REFERENCE   2  (bases 1 to 712)
  CONSRTM   NCBI RefSeq Targeted Loci Project
  TITLE     Direct Submission
  JOURNAL   Submitted (01-MAY-2018) National Center for Biotechnology
            Information, NIH, Bethesda, MD 20894, USA
REFERENCE   3  (bases 1 to 712)
  AUTHORS   Tanney,J.B., Visagie,C.M., Yilmaz,N. and Seifert,K.A.
  TITLE     Direct Submission
  JOURNAL   Submitted (21-APR-2017) Biodiversity (Mycology), Agriculture and
            Agri-Food Canada, 960 Carling Avenue, Ottawa, Ontario K1A0C6,
            Canada
```

Figure S3. Blast search (closet match) for CMB-M0339

1 Phylogenetic tree

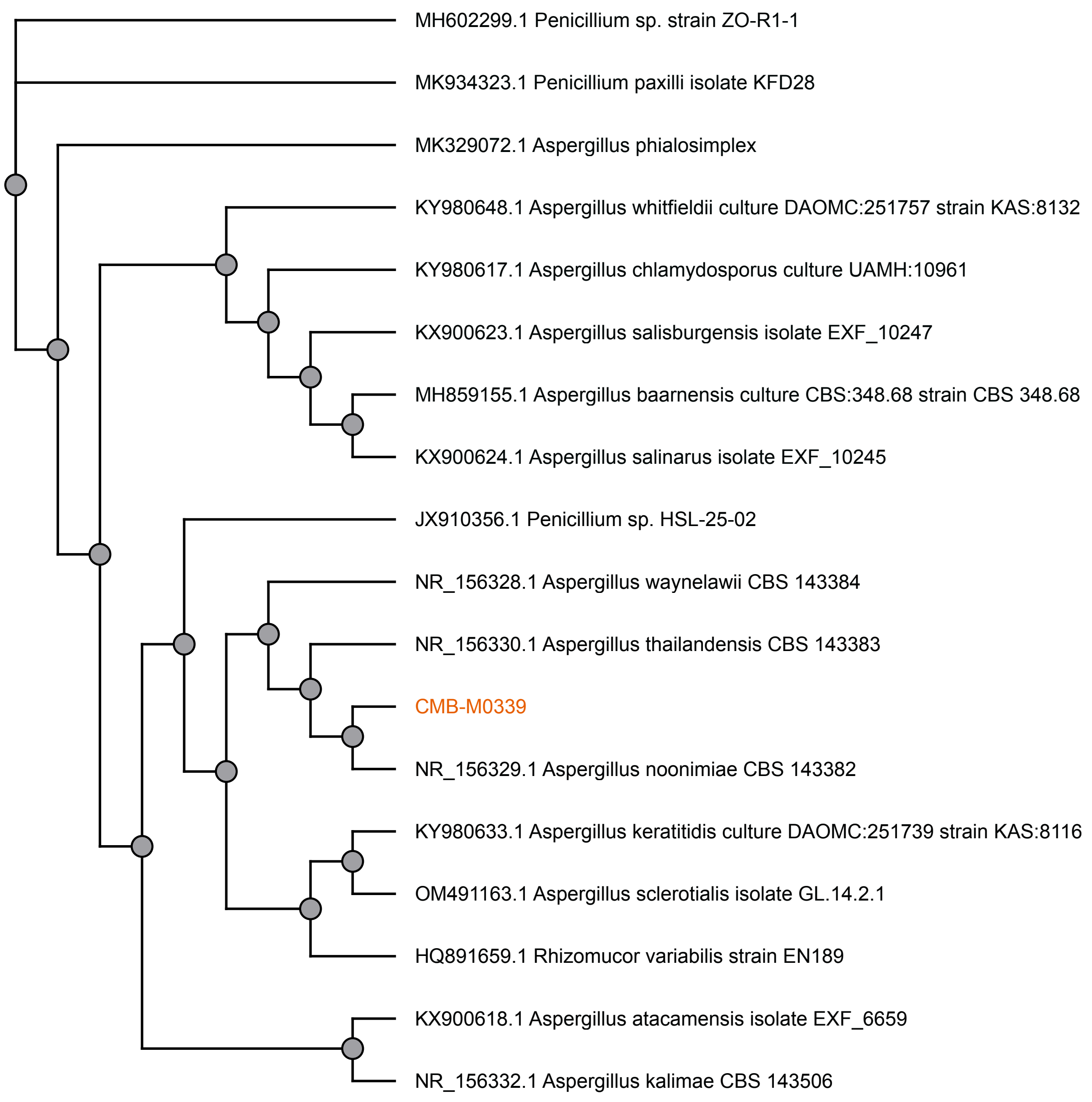

Figure S4. Phylogenetic tree by PhyML Maximum Likehood analysis of 18s rRNA sequences showing the relationship of CMB-M0339 among selected reference strains (RefSeq GenBank) with the accession numbers

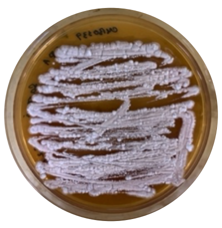

Figure S5. CMB-M0339 cultivated on SD agar

2 Chemical investigation of CMB-M0339

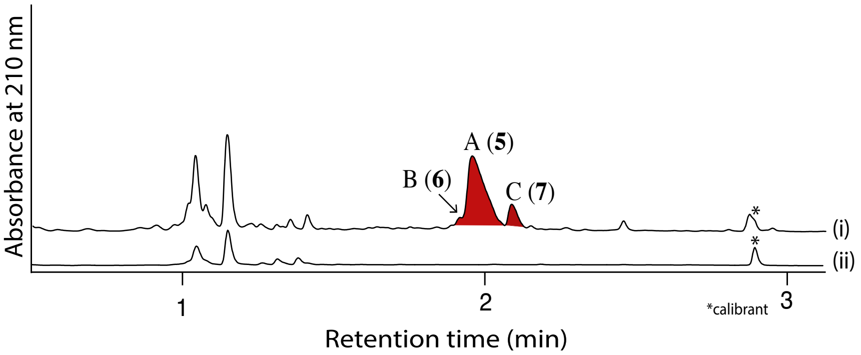

Figure S6. UPLD-DAD chromatograms of (i) crude extract of CMB-M0339 cultivated on M1 agar (supplemented with 3.3% artificial sea salt) (ii) Media blank

The crude extract of *Aspergillus* sp. CMB-M0339 cultivated on M1 agar (supplemented with 3.3% artificial sea salt) was subjected to UPLC-DAD analysis which revealed the production of one major metabolite, noonindole A (**5**) and two minor metabolites, noonindoles B (**6**) and C (**7**) sharing similar UV-Vis chromophore.

3 MATRIX cultivation profiling of CMB-M0339

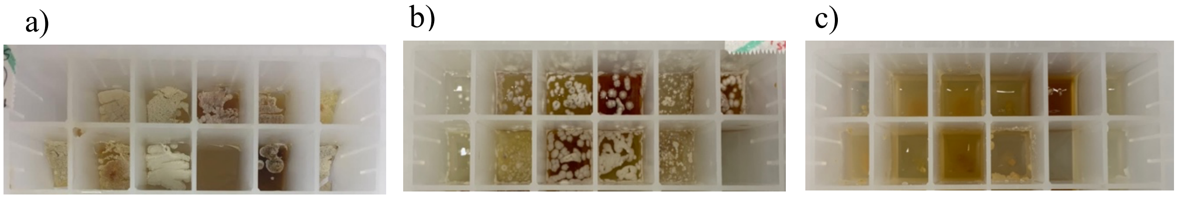

Figure S7. CMB-M0339 cultivated under MATRIX conditions. (a) agar (b) static broth (c) shaken broth

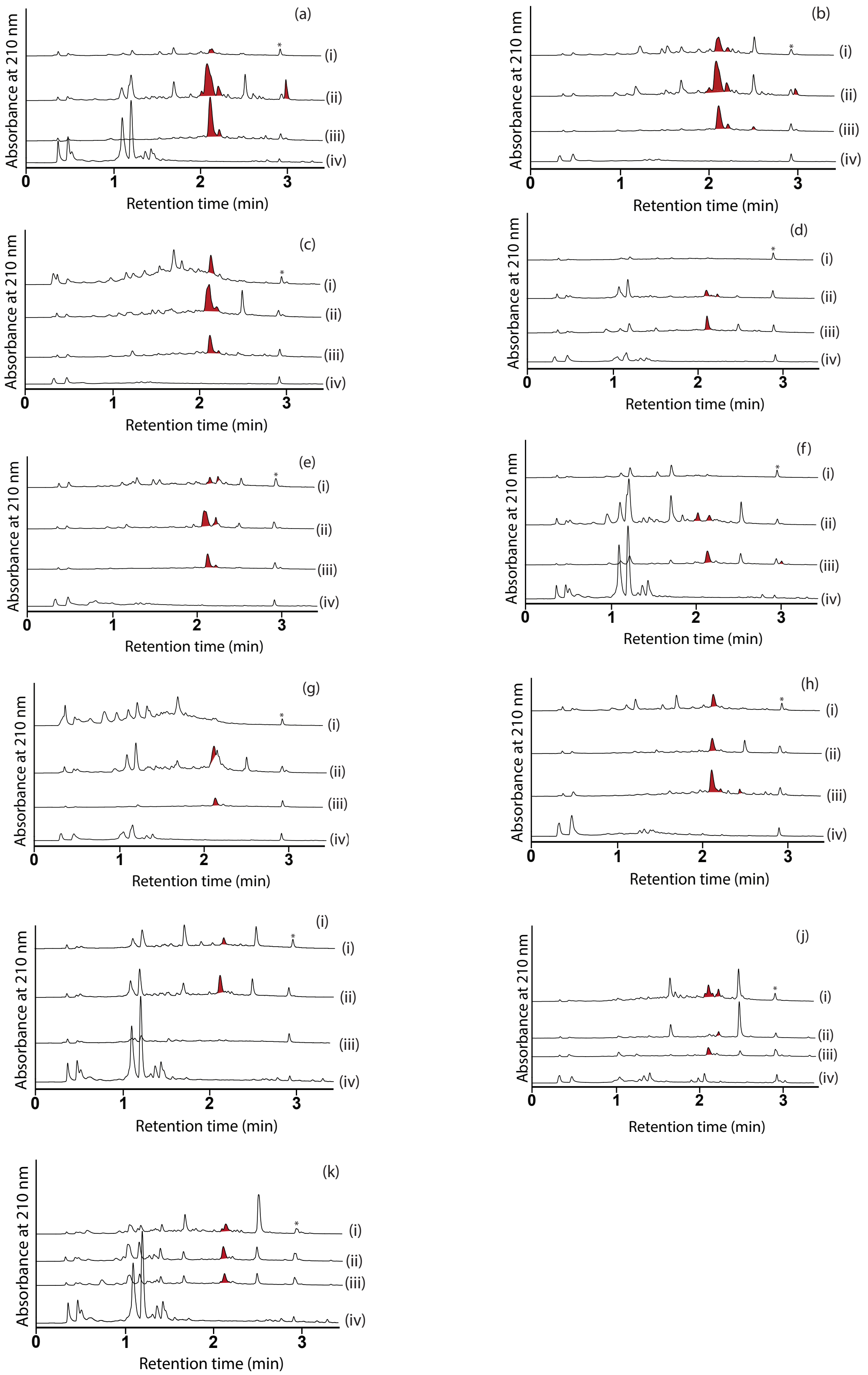

Figure S8. UPLC-DAD chromatograms of MATRIX extracts of CMB-M0339 showing the production of noonindoles A–C **(5–7)** (highlighted in red) in different media and culture conditions; (a) D400, (b) GY, (c) IM ,(d) M1, (e) M2, (f) SGG, (g) YEME, (h) YES, (i) 333, (j) PD, (k) SD, (i) shaken broth, (ii) static broth, (iii) agar, (iv) Media blank, * Internal calibrant

Table S1. Composition of media used for cultivation profiling

Medium	Composition (per Litre)
**M1**	Peptone (2.0 g), yeast extract (4.0 g), starch (10.0 g), artificial sea salt (33.0 g), agar (18.0 g)
**M2**	Mannitol (40.0 g), maltose (40.0 g), yeast extract (10.0 g), K_2HPO_4 (2.0 g), $MgSO_4.7H_2O$ (0.5 g), $FeSO_4.7H_2O$ (0.01 g), agar (18.0 g),
**IM**	Yeast extract (Difco) (4.0 g), malt extract (Difco) (10.0 g), glucose (country brewers) (4.0 g), mannitol (Amyl) 40.0 g, agar (Amyl) (18.0 g)
**Modified YEME**	Bacto peptone (Difco) (5.0 g), yeast extract (Difco) (3.0 g), Oxoin malt extract (3.0 g), glucose (10.0 g), sucrose (170.0 g), agar (18.0 g)
**GY**	Yeast extract (Difco) (4.0 g), malt extract (Difco) (10.0 g), glucose (country brewers) (4.0 g), $CaCO_3$ (Univar Ajax) (2.0 g), soluble starch (Difco) (20.0 g), agar (Amyl) (18.0 g)
**YES**	Sucrose (150 g), yeast extract (20 g), $MgSO_4.7H_2O$ (0.5 g), $ZnSO_4.7H_2O$ (0.01 g), $CuSO_4.5H_2O$ (0.005 g), agar (18.0 g)
**D400**	Glucose (10.0 g), malt extract (3.0 g), peptone (3.0 g), soluble starch (20.0 g), yeast extract (5.0 g), $CaCO_3$ (3.0 g), agar (18.0 g).
**SGG**	Glucose (10.0 g), glycerol (10.0 g), cornsteep powder (2.5 g), peptone (5.0 g), soluble starch 10.0 g), yeast extract (2.0 g), $CaCO_3$ (3.0 g), NaCl (1.0 g), agar (18.0 g).
**333**	Glucose (5.0 g), peptone (3.0 g), soluble starch (10.0 g), yeast extract (3.0 g), $CaCO_3$ (2.0 g), agar (18.0 g).
**PD**	Potato extract (4.0 g), dextrose (20.0 g), agar (18 g)
**SD**	Peptic digest of animal tissue (5.0 g), pancreatic digest of casein (5.0 g), dextrose (40.0 g), agar (18 g)

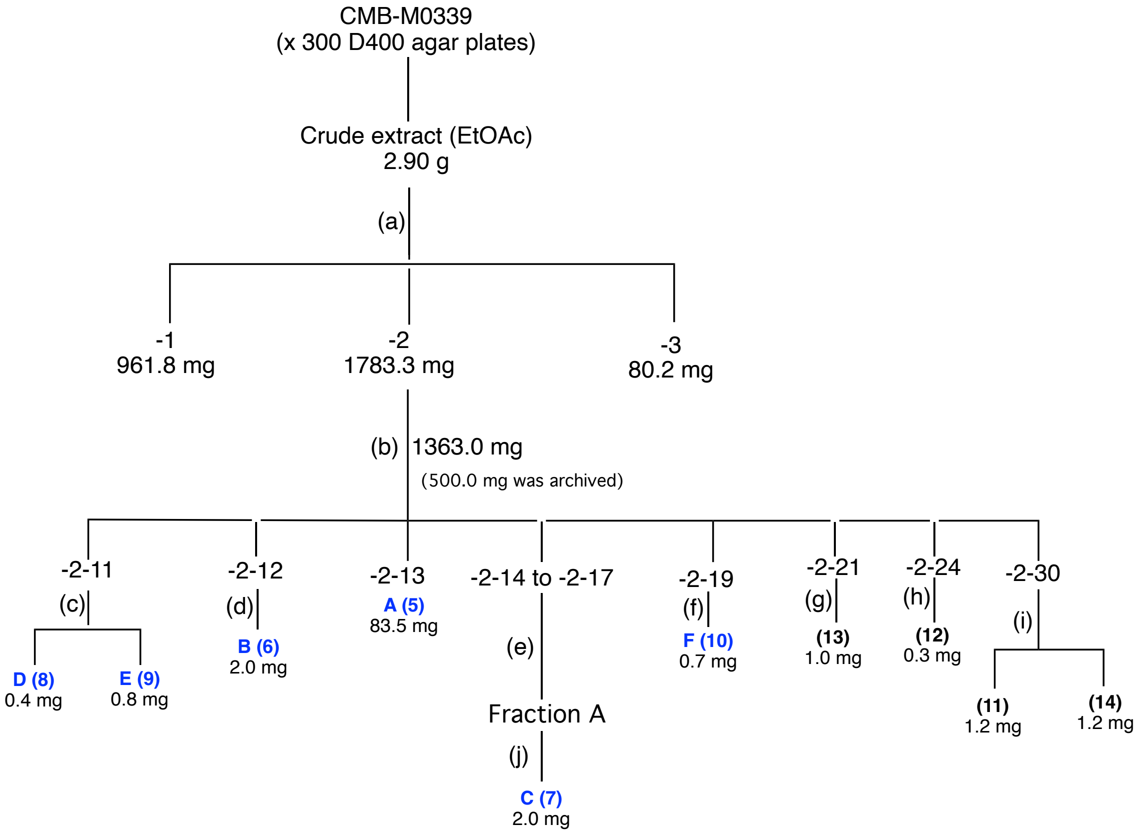

Figure S9. Isolation scheme for **5-14** from CMB-M0339. (a) Trituration [hexane (-1), DCM (-2), MeOH (-3)]. (b) Preparative HPLC (Phenomenex Luna-C_8 10 µm, 21.2 × 250 mm column, 20 mL/min gradient elution from 90% H_2O/MeCN to 100% MeCN over 20 min with constant 0.1% TFA modifier). (c) Semi preparative HPLC (Zorbax C_{18} 5µm column, 9.4 × 250 mm, 3 mL/min isocratic elution of 40% MeCN/H_2O over 30 min with constant 0.1% TFA modifier. (d) Semi preparative HPLC (Zorbax C_8 5µm column, 9.4 × 250 mm, 3 mL/min isocratic elution of 37% MeCN/H_2O over 20 min with constant 0.1% TFA modifier. (e) Column chromatography: Sep-Pak (Agilent Bond Elut C_{18} column, 5 g) gradient elution from 90% H_2O/MeCN to 100% MeCN (f) Semi preparative HPLC (Agilent C_8-Ep 5µm column, 9.4 × 250 mm, 3 mL/min isocratic elution of 50% MeCN/H_2O over 25 min with constant 0.1% TFA modifier. (g) Semi preparative HPLC (Zorbax C_{18} 5µm column, 9.4 × 250 mm, 3 mL/min isocratic elution of 85% MeCN/H_2O over 15 min with constant 0.1% TFA modifier. (h) Semi preparative HPLC (Agilent C_8-Ep 5µm column, 9.4 × 250 mm, 3 mL/min isocratic elution of 60% MeCN/H_2O over 25 min with constant 0.1% TFA modifier. (i) Semi preparative HPLC (Agilent CN 5µm column, 9.4 × 250 mm, 3 mL/min isocratic elution of 60% MeCN/H_2O over 20 min with constant 0.1% TFA modifier. (j) Zorbax C_8 5µm column, 9.4 × 250 mm, 3 mL/min isocratic elution of 40% MeCN/H_2O over 20 min with constant 0.1% TFA modifier

4 Noonindole A (5)

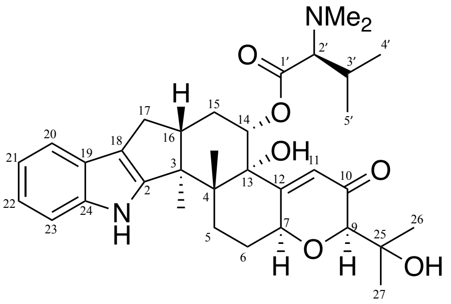

Table S2. 1D and 2D NMR (methanol-d_4, 600 MHz) data for noonindole A (**5**)

Pos.	$\delta_{H,}$ mult. (J in Hz)	δ_C	COSY	^{1}H-^{13}C HMBC	ROESY
1-NH	-	-	-	-	-
2	-	152.4	-	-	-
3	-	52.0	-	-	-
4	-	46.4	-	-	-
5	*a* 2.87 (ddd, 1H, J =*13.6, 13.6, 5.2* Hz)	28.1	5*b*, 6*a*, 6*b*	4, 6, 13, 3	3-Me
	b 1.81 (br dd, 1H, J =*13.6, 5.2* Hz)		5*a*, 6*b*	4, 6, 7, 13	
6	*a* 2.38 (m, 1H)	30.6	5*a*, 6*b*, 7	5, 7, 4, 12	-
	b 1.91 (m, 1H)		5*a*, 6*a*, 7	5, 7	
7	4.91 (m, 1H)	74.6	6, 9, 11	6, 11, 12	9
9	3.68 (d, 1H, J = *1.9* Hz)	84.0	7	7, 25, 26, 27, 10	7, 27
10	-	198.5	-	-	-
11	5.62 (d, 1H, J =*1.9* Hz)	122.4	7	7, 9, 13	-
12	-	165.9	-	-	-
13	-	80.0	-	-	-
14	5.53 (dd, 1H, J = *10.2, 5.3* Hz)	77.9	15*a*, 15*b*	15, 1′	16
15	*a* 2.24 (dd, 1H, J = *11.1, 10.2* Hz)	28.5	14, 15*b*, 16	3, 13, 14, 16	3-Me
	b 2.21 (m, 1H)		14, 15*a*, 16	3, 13, 14, 16	
16	2.92 (m, 1H)	47.4	15*a*, 15*b*, 17*a*	17, 3-Me, 3	14
17	*a* 2.73 (dd, 1H, J = *13.0, 6.2* Hz)	27.6	16, 17*b*[a]	2, 3, 15, 16, 18	-
	b 2.47[a] (m, 1H)		-	-	
18	-	117.4	-	-	-
19	-	126.2	-	-	-
20	7.31 (d, 1H, J = *7.5* Hz)	119.0	21	18, 22, 24	-
21	6.94 (ddd, 1H, J = *7.5, 7.5, 1.1* Hz)	120.1	20, 22	19, 23	-
22	6.98 (ddd, 1H, J = *7.5, 7.5, 1.1* Hz)	121.1	21, 23	20, 24	-
23	7.27 (d, 1H, J = *7.5* Hz)	112.7	22	19, 21	-
24	-	141.8	-	-	-
25	-	73.3	-		-
26	1.28 (s, 3H)	25.4	-	9, 25, 27	-
27	1.26 (s, 3H)	26.2	-	9, 25, 26	9
1′	-	167.4	-	-	-
2′	3.86 (d, 1H, J = *5.5* Hz)	74.8	3′[a]	1′, 3′, 4′ [b], 5′, NMe$_2$	NMe$_2$
3′	2.47[a] (m, 1H)	28.4	-	-	-
4′	1.05[b]	19.4	3′[a]	5′	-
5′	1.03 (d, 1H, J = *7.1* Hz)	17.0	3′[a]	4′[b]	-
3-Me	1.37 (s, 3H)	16.7	-	2, 3, 4, 16	15*a*, 5*a*
4-Me	1.04[b]	19.4	-	13, 3, 4	-
NMe$_2$	2.90 (s, 6H)	43.0	-	2′, NMe$_2$	2′

[a-b] Resonances with the same superscript within a column are overlapping and assignments may be interchanged

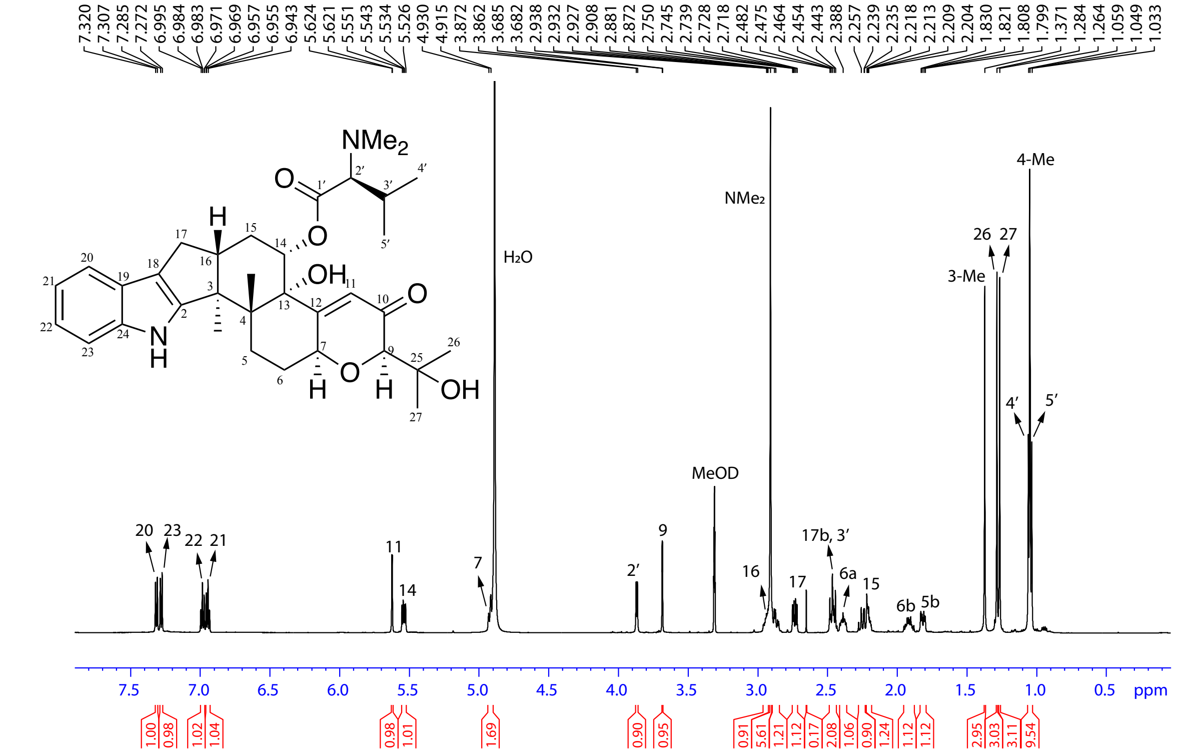

Figure S10. ^{1}H NMR (600 MHz, methanol-d_4) spectrum of noonindole A (**5**)

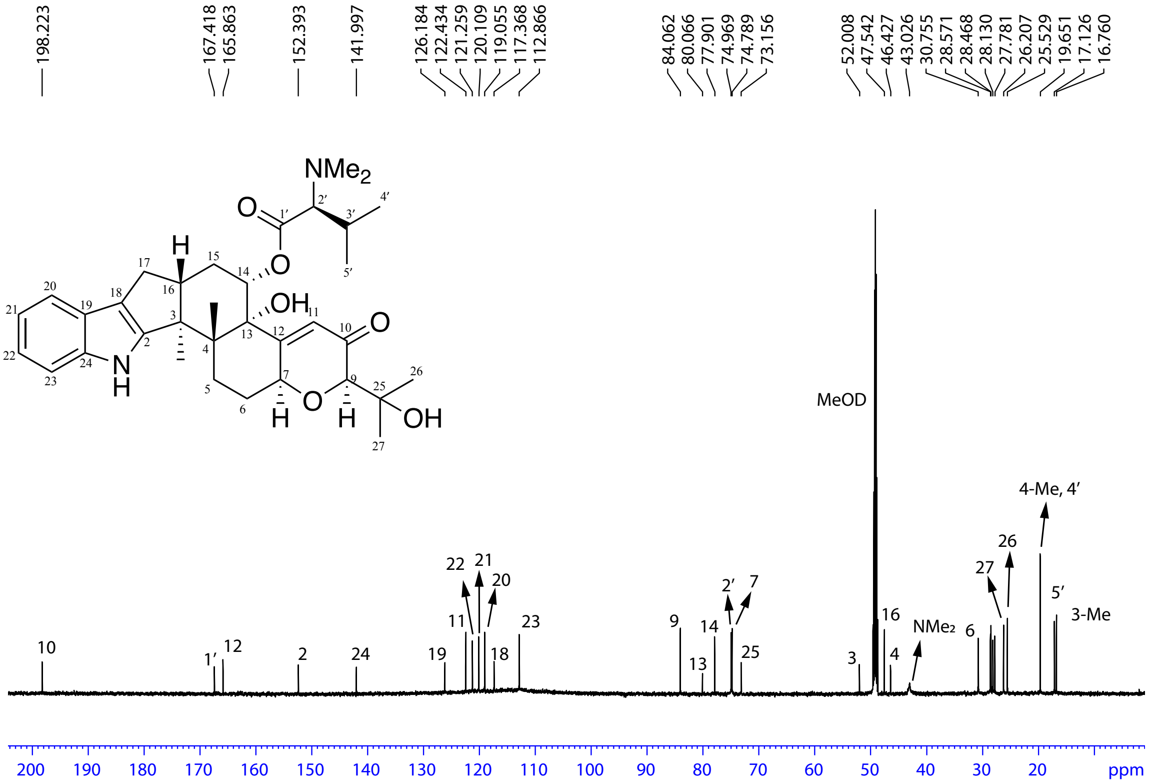

Figure S11. ^{13}C NMR (150 MHz, methanol-d_4) spectrum of noonindole A (**5**)

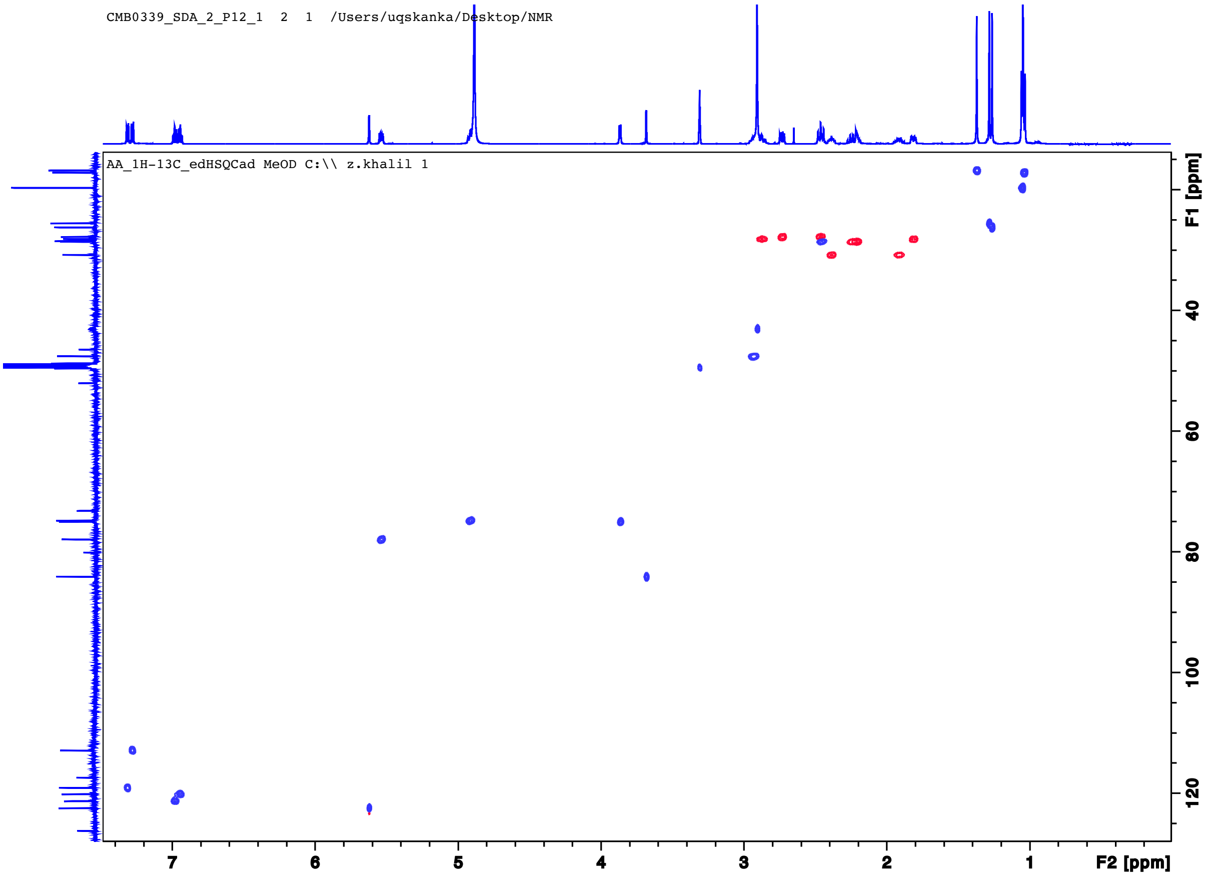

Figure S12. HSQC (methanol-d_4) spectrum of noonindole A (**5**)

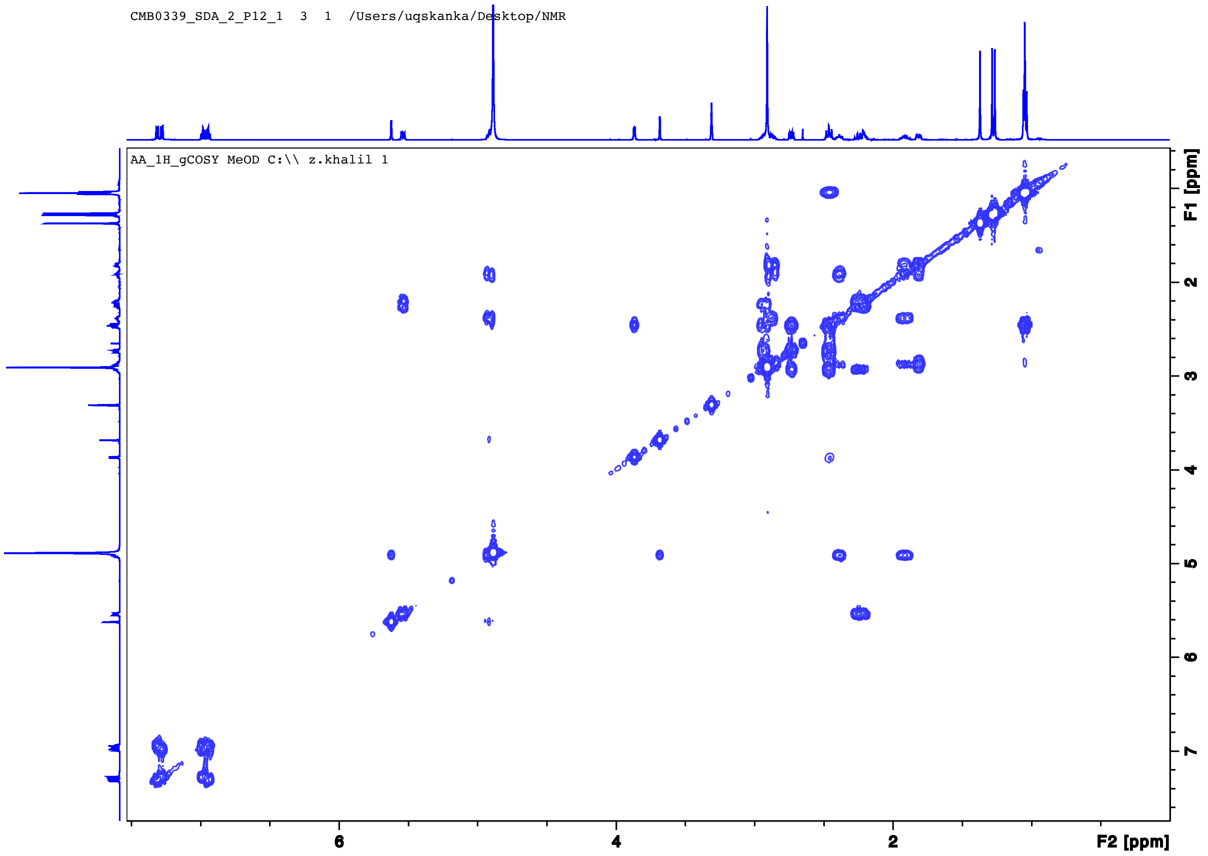

Figure S13. COSY (methanol-d_4) spectrum of noonindole A (**5**)

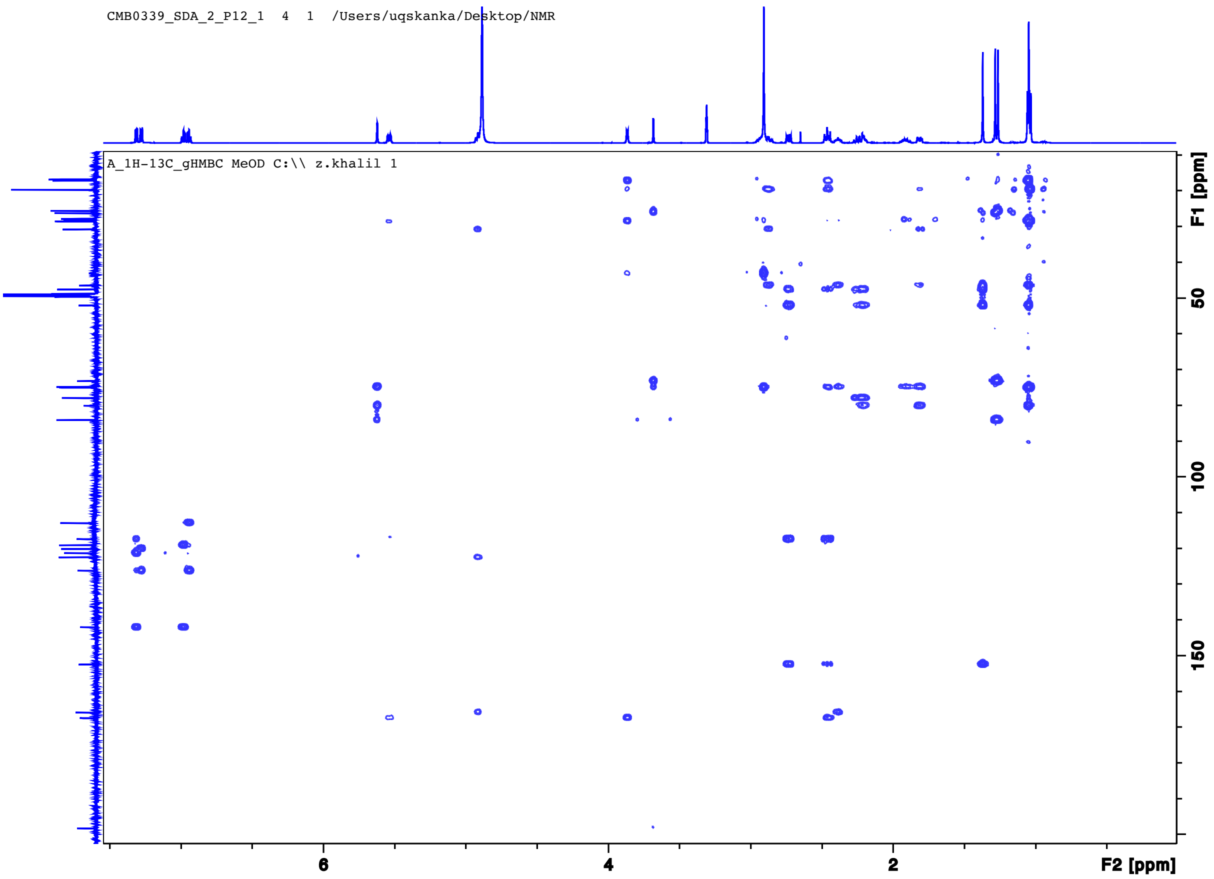

Figure S14. HMBC (methanol-d_4) spectrum of noonindole A (**5**)

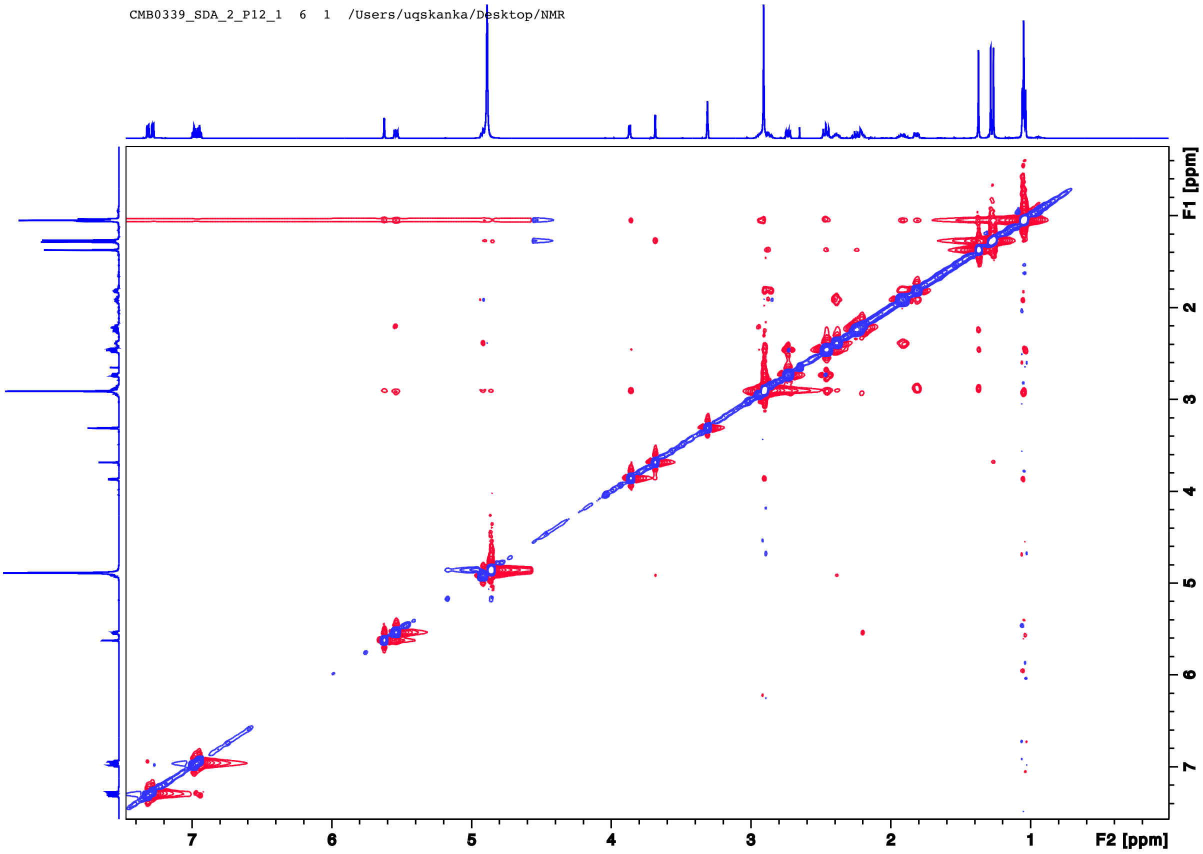

Figure S15. ROESY (methanol-d_4) spectrum of noonindole A (**5**)

Mass Spectrum Molecular Formula Report

Analysis Info

Analysis Name D:\Data\s.kankanamge\CMB0339_SDA_2_P12.d
Method tune-medhigh_AP.m
Sample Name CMB0339_SDA_2_P12
Comment

Acquisition Date 10/28/2020 4:38:30 PM
Operator a.salim
Instrument / Ser# micrOTOF 213750.00232

Acquisition Parameter

Source Type	ESI	Ion Polarity	Positive	Set Nebulizer	0.5 Bar
Focus	Not active			Set Dry Heater	180 °C
Scan Begin	100 m/z	Set Capillary	4500 V	Set Dry Gas	5.0 l/min
Scan End	1500 m/z	Set End Plate Offset	-500 V	Set Divert Valve	Source

Generate Molecular Formula Parameter

Formula, min.					
Formula, max.					
Measured m/z		Tolerance		Charge	
Check Valence		Minimum		Maximum	
Nirogen Rule		Electron Configuration			
Filter H/C Ratio		Minimum		Maximum	
Estimate Carbon					

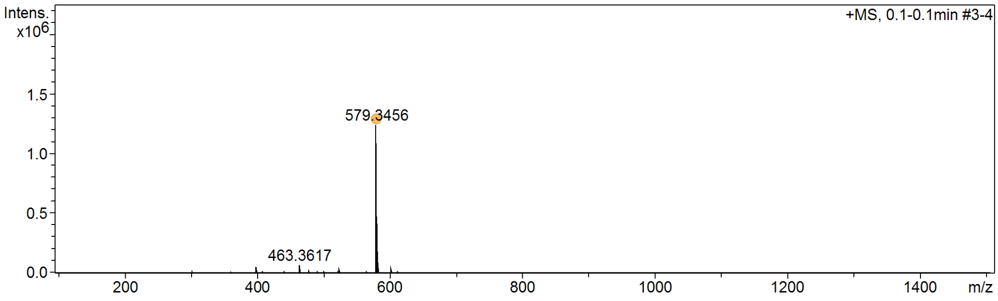

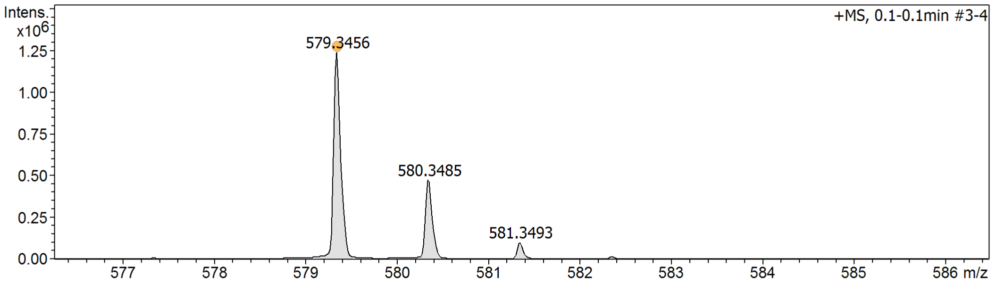

Meas. m/z	#	Ion Formula	m/z	err [ppm]	mSigma	# Sigma	Score	rdb	e⁻ Conf	N-Rule
579.3456	1	C33H47N4O5	579.3541	-14.6	1.8	1	0.01	12.5	even	ok
	2	C34H47N2O6	579.3429	-4.8	2.0	2	41.79	12.5	even	ok
	3	C31H39N12	579.3415	-7.1	4.4	3	9.52	18.5	even	ok
	4	C34H43N8O	579.3554	-16.9	10.0	4	0.00	17.5	even	ok
	5	C30H43N8O4	579.3402	-9.4	11.9	5	1.34	13.5	even	ok
	6	C35H43N6O2	579.3442	2.5	12.1	6	100.00	17.5	even	ok
	7	C32H51O9	579.3528	12.3	13.5	7	0.08	7.5	even	ok
	8	C29H43N10O3	579.3514	10.0	14.4	8	0.80	13.5	even	ok

Bruker Compass DataAnalysis 4.1 printed: 10/28/2020 5:38:42 PM Page 1 of 2

Figure S16. HRMS spectrum of noonindole A (**5**)

5 Noonindole B (6)

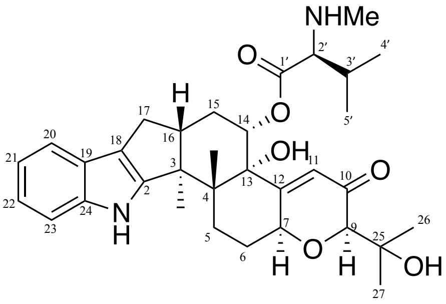

Table S3. 1D and 2D NMR (methanol-d_4, 600 MHz) data for noonindole B (**6**)

Pos.	$\delta_{H,}$ mult. (J in Hz)	δ_C	COSY	^{1}H-^{13}C HMBC	ROESY
1-NH	-	-	-	-	-
2	-	152.3	-	-	-
3	-	51.9	-	-	-
4	-	46.2	-	-	-
5	*a* 2.88 (ddd, 1H, J = *13.5, 13.5, 5.1* Hz)	28.0	5*b*, 6*a*, 6*b*	3, 4, 6, 13, 4-Me	3-Me
	b 1.83 (br dd, 1H, J = *13.5, 5.1* Hz)		5*a*, 6*a*, 6*b*	4, 6, 7, 13, 4-Me	-
6	*a* 2.39 (m, 1H)	30.5	5*a*, 6*b*, 7	5, 7, 4, 12	-
	b 1.93 (m, 1H)		5*a*, 5*b*, 6*a*, 7	4, 5, 7	
7	4.90 (m, 1H)	74.8	6, 9, 11	6, 11, 12	-
9	3.72 (d, 1H, J = *1.5* Hz)	83.9	7	7, 25, 26, 27	26, 27
10	-	198.1	-	-	-
11	5.57 (d, 1H, J = *1.5* Hz)	122.0	7	7, 9, 13	-
12	-	165.8	-	-	-
13	-	80.0	-	-	-
14	5.50 (dd, 1H, J = *10.4, 5.*0 Hz)	77.3	15*a*, 15*b*	15, 1′	16, 4-Me
15	*a* 2.25 (dd, 1H, J = *13.0, 10.4* Hz)	28.3	14, 15*b*, 16	3, 13, 14, 16	3-Me
	b 2.17 (m, 1H)		14, 15*a*, 16	3, 13, 14, 16	-
16	2.98 (m, 1H)	47.5	15*a*, 17*a*, 17*b*	14, 17, 3-Me, 3	14, 4-Me
17	*a* 2.74 (dd, 1H, J = *13.1, 6.3* Hz)	27.6	16, 17*b*	2, 3, 16, 18	3-Me
	b 2.47 (dd, 1H, J = *13.1, 11.1* Hz)		16, 17*a*	2, 15, 16, 18	-
18	-	117.3	-	-	-
19	-	126.0	-	-	-
20	7.30 (d, 1H, J = *7.9* Hz)	118.8	21	18, 22, 24	-
21	6.93 (br dd, 1H, J = *7.9,7.7* Hz)	119.9	20, 22	19, 23	-
22	6.97 (ddd, 1H, J = *7.9,7.9, 0.9* Hz)	121.1	21, 23	20, 24	-
23	7.28 (d, 1H, J = *7.9* Hz)	112.7	22	19, 21	-
24	-	141.9	-	-	-
25	-	73.0	-		-
26	1.28 (s, 3H)	25.3	-	9, 25, 27	9
27	1.26 (s, 3H)	26.4	-	9, 25, 26	9
1′	-	168.9	-	-	-
2′	3.90 (br s, 1H)	68.7	3′	-	-
3′	2.31 (m, 1H)	30.7	2′, 4′, 5′	-	-
4′	1.09 (br d, 1H, J = *5.3* Hz)	18.7	3′	2′, 3′, 5′	-
5′	0.99 (d, 1H, J = *7.0* Hz)	17.7	3′	2′, 3′, 4′	-
3-Me	1.37 (s, 3H)	16.6	-	2, 3, 4, 16	5*a*, 15*a*, 17*a*
4-Me	1.10 (s, 3H)	19.5	-	3, 4, 5, 13	16, 14
NMe	2.72 (s, 3H)	33.8	-	2′	-

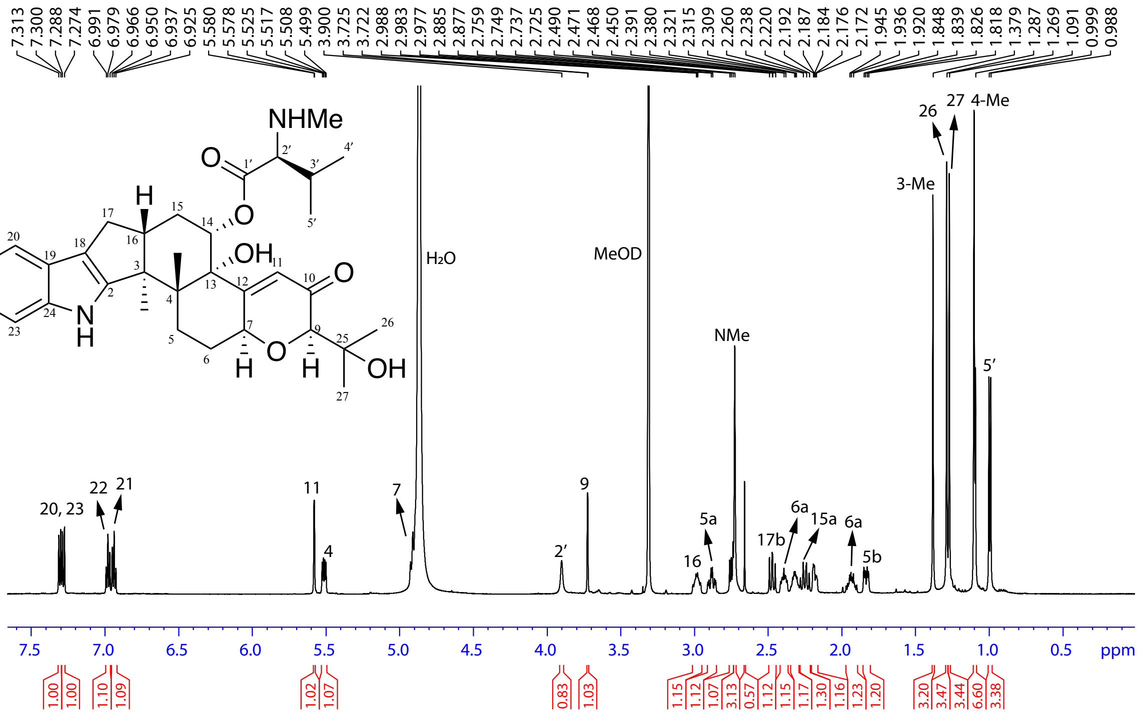

Figure S17.^{1}H NMR (600 MHz, methanol-d_4) spectrum of noonindole B (**6**)

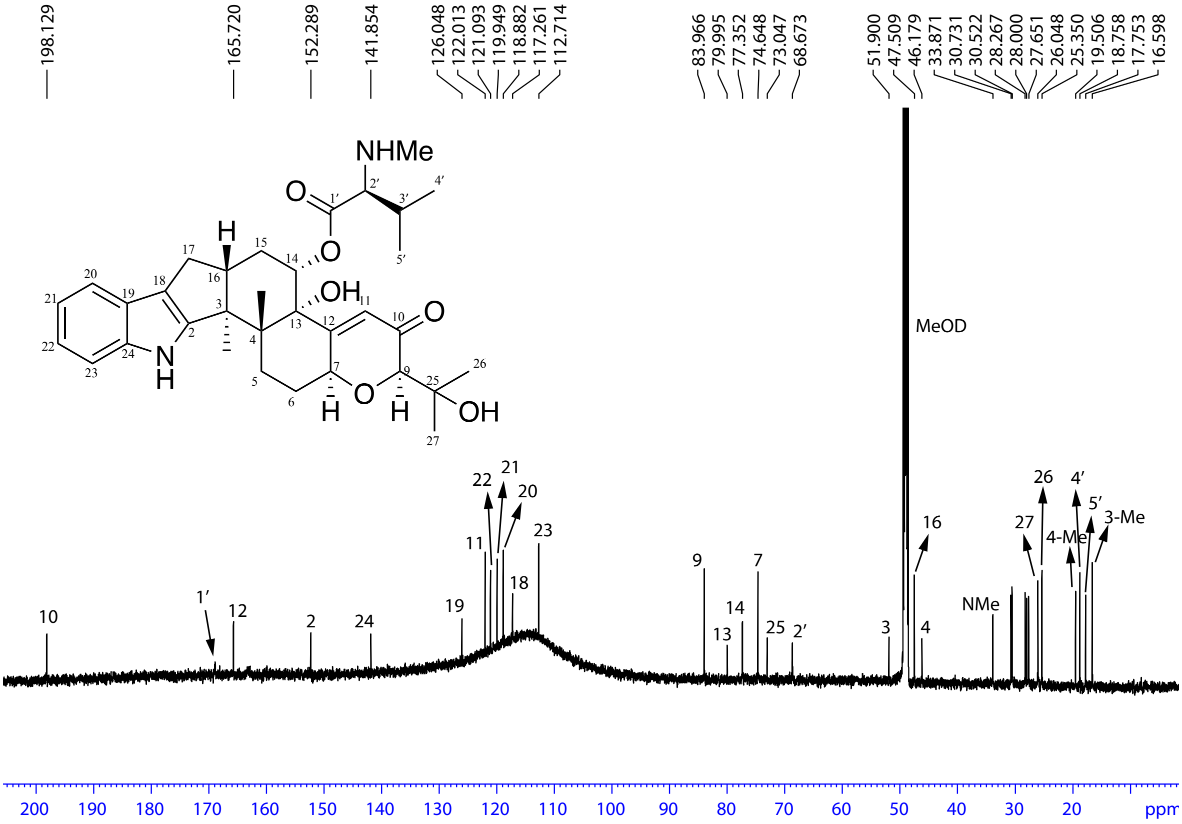

Figure S18. ^{13}C NMR (150 MHz, methanol-d_4) spectrum of noonindole B (**6**)

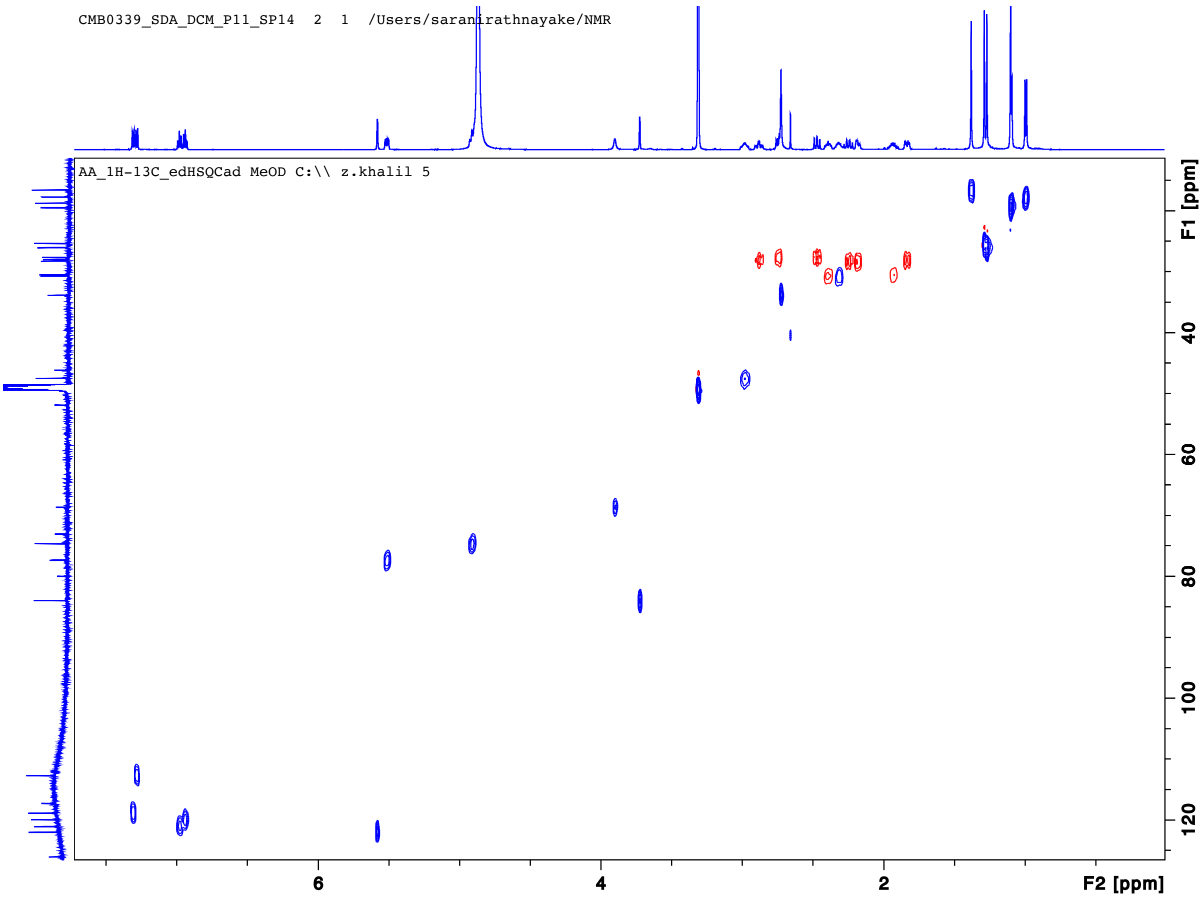

Figure S19. HSQC (methanol-d_4) spectrum of noonindole B (**6**)

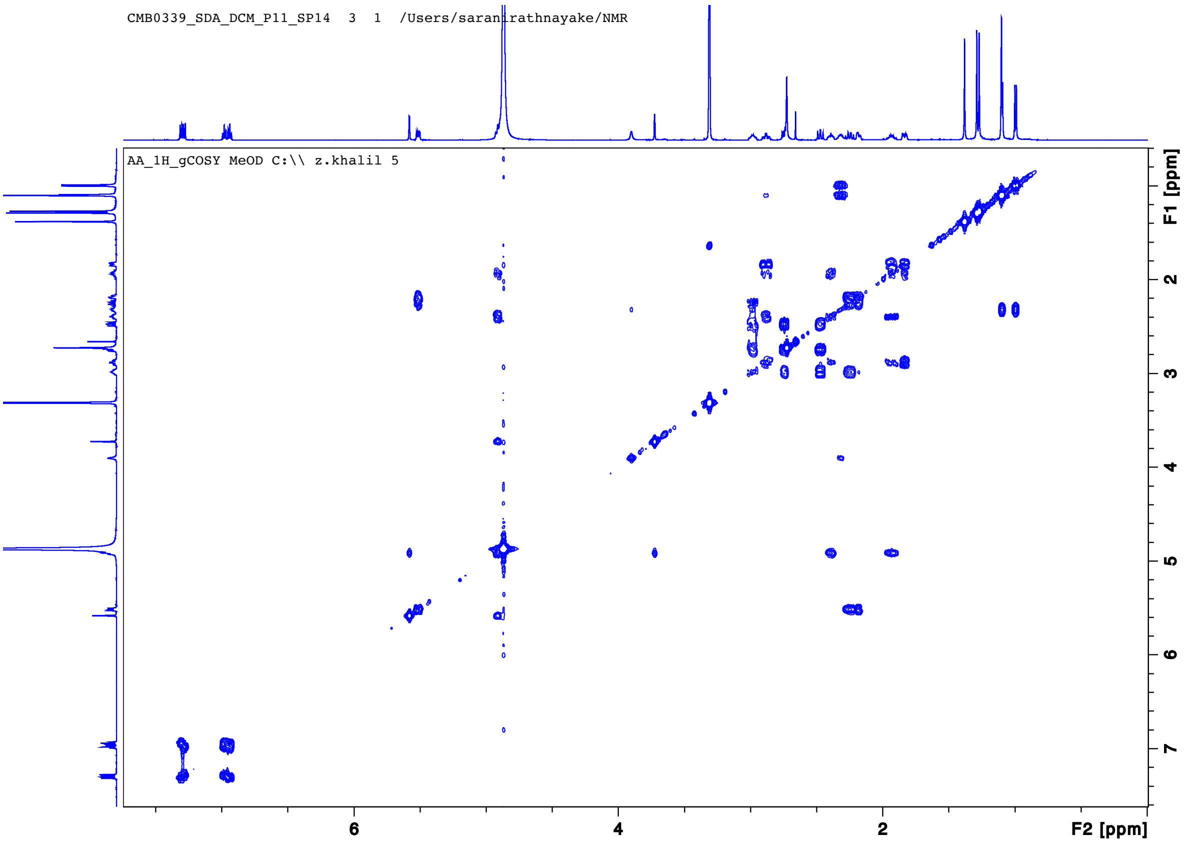

Figure S20. COSY (methanol-d_4) spectrum of noonindole B (**6**)

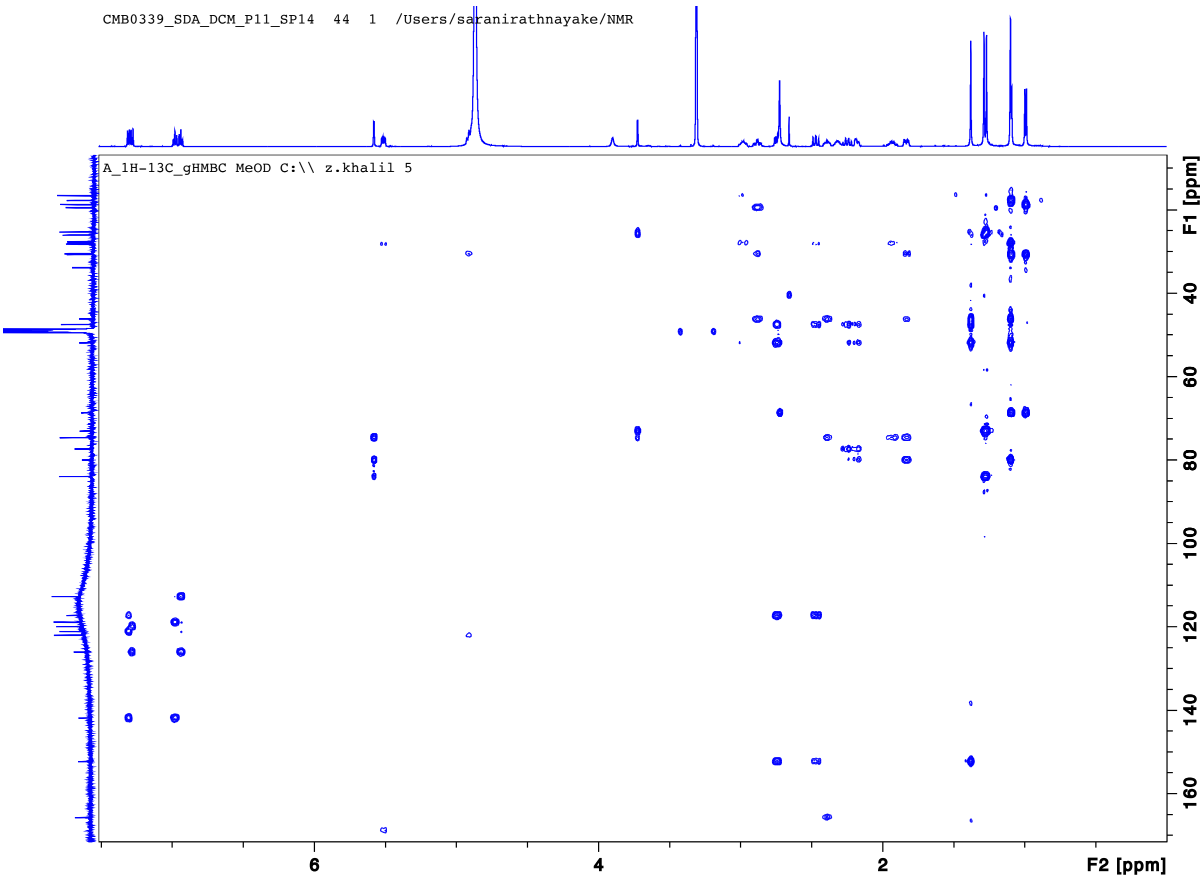

Figure S21. HMBC (methanol-d_4) spectrum of noonindole B (**6**)

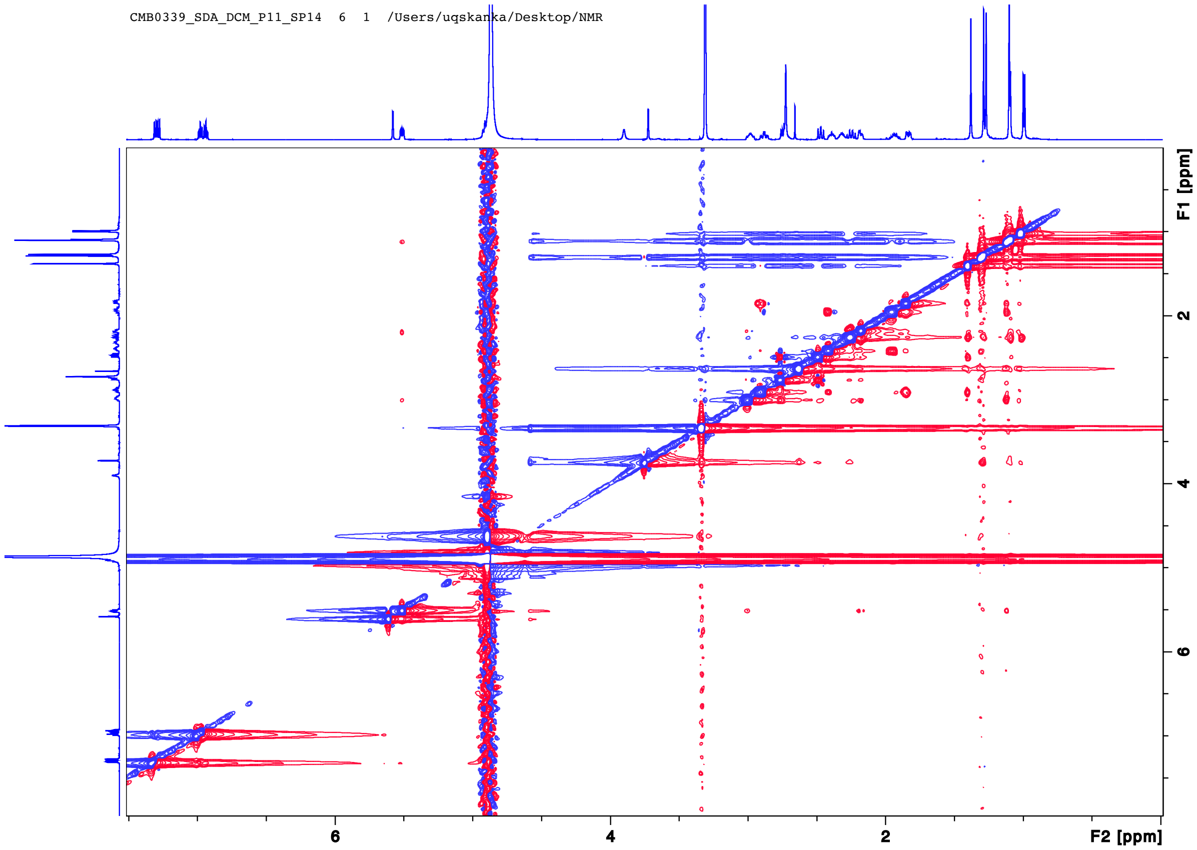

Figure S22. ROESY (methanol-d_4) spectrum of noonindole B (**6**)

Mass Spectrum Molecular Formula Report

Analysis Info

Analysis Name	D:\Data\s.kankanamge\CMB0339_SDA_P11_SP14_E.d	Acquisition Date	8/4/2022 12:03:32 PM
Method	tune-med_AP.m	Operator	a.salim
Sample Name	CMB0339_SDA_P11_SP14_E	Instrument / Ser#	micrOTOF 213750.00232
Comment			

Acquisition Parameter

Source Type	ESI	Ion Polarity	Positive	Set Nebulizer	0.8 Bar
Focus	Not active			Set Dry Heater	180 °C
Scan Begin	100 m/z	Set Capillary	4500 V	Set Dry Gas	5.0 l/min
Scan End	1000 m/z	Set End Plate Offset	-500 V	Set Divert Valve	Source

Generate Molecular Formula Parameter

Formula, min.		
Formula, max.		
Measured m/z	Tolerance	Charge
Check Valence	Minimum	Maximum
Nirogen Rule	Electron Configuration	
Filter H/C Ratio	Minimum	Maximum
Estimate Carbon		

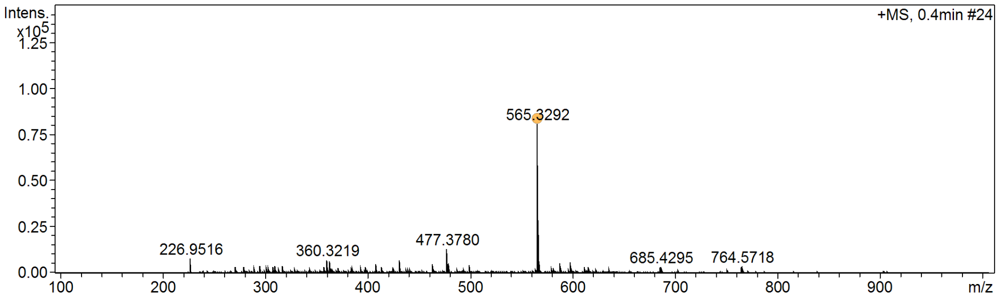

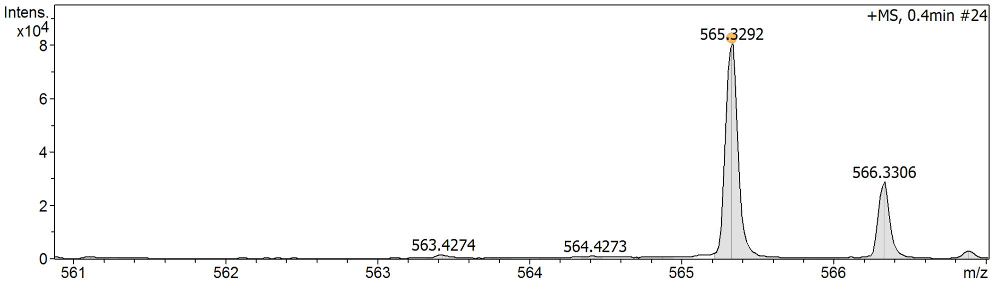

Meas. m/z	#	Ion Formula	m/z	err [ppm]	mSigma	# Sigma	Score	rdb	e⁻ Conf	N-Rule
565.3292	1	C33H45N2O6	565.3272	3.5	7.0	1	53.94	12.5	even	ok
	2	C34H41N6O2	565.3286	1.1	18.5	2	100.00	17.5	even	ok
	3	C38H45O4	565.3312	3.6	30.9	3	30.65	16.5	even	ok

Bruker Compass DataAnalysis 4.1 printed: 8/4/2022 12:05:53 PM Page 1 of 1

Figure S23. HRMS spectrum of noonindole B (**6**)

6 Noonindole C (7)

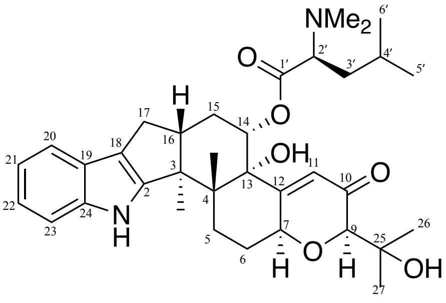

Table S4.1D and 2D NMR (in methanol-d_4, 600 MHz) data for noonindole C (**7**)

Pos.	δ_H, mult. (*J* in Hz)	δ_C	COSY	^{1}H-^{13}C HMBC	ROESY
1-NH	-	-	-	-	-
2	-	152.2	-	-	-
3	-	51.8	-	-	-
4	-	46.2	-	-	-
5	*a* 2.87 (ddd, 1H, J = *13.8, 13.8, 5.4* Hz)	27.9	5*b*, 6*a*, 6*b*	4, 6, 4-Me	-
	b 1.84 (br dd, 1H, J = *13.8, 5.4* Hz)		5*a*, 6*a*, 6*b*	4, 6, 7, 4-Me, 13	-
6	*a* 2.39 (m, 1H)	30.5	5*a*, 6*b*, 7	7, 4, 12	-
	b 1.93 (m, 1H)		5*a*, 6*a*, 6*b*, 7	4, 5, 7	-
7	4.92 (br t, 1H, J = *8.5* Hz)	74.8	6, 9, 11	6, 11	9
9	3.71 (br s, 1H)	84.0	7	7, 25, 26	7
10	-	197.9	-	-	-
11	5.57 (d, 1H, J = *1.9* Hz)	121.8	7	7, 9, 13	-
12	-	165.0	-	-	-
13	-	80.1	-	-	-
14	5.51 (dd, 1H, J =*10.4, 5.1* Hz)	77.5	15*a*, 15*b*	1′	4-Me, 16
15	*a* 2.25 (dd, 1H, J = *13.2, 10.4* Hz)	28.1	14, 15*b*, 16	3, 14, 16	3-Me
	b 2.20 (m, 1H)		14, 15*a*, 16	3, 14, 13, 16	-
16	2.97 (m, 1H)	47.5	15, 17*b*	15	4-Me, 14
17	*a* 2.74 (dd, 1H, J = *13.0, 6.3* Hz)	27.6	16, 17*b*	2, 3, 16, 18	3-Me
	b 2.47 (dd, 1H, J = *13.0, 10.8* Hz)		16, 17*a*	2, 16, 18	-
18	-	117.2	-	-	-
19	-	126.1	-	-	-
20	7.30 (d, 1H, J = *7.8* Hz)	118.9	21	18, 22, 24	-
21	6.93 (ddd, 1H, J = *7.8, 7.8, 1.1* Hz)	119.9	20, 22	19, 23	-
22	6.98 (ddd, 1H, J = *7.8, 7.8, 1.1* Hz)	121.1	21, 23	20, 24	-
23	7.28 (d, 1H, J = *7.8* Hz)	112.7	22	19, 21	-
24	-	141.9	-	-	-
25	-	73.0	-		-
26	1.30 (s, 3H)	25.5	-	9, 25, 27	-
27	1.27 (s, 3H)	25.8	-	9, 25, 26	-
1′	-	168.9	-	-	-
2′	4.02 (m, 1H)	67.5	3′	-	6′
3′	*a* 1.90 (m, 1H)	37.4	2′, 3′ *b*, 4′	4′	5′
	b 1.69, (m, 1H)		2′, 3′ *a*, 4′	-	5′, 6′, NMe$_2$
4′	1.56 (m, 1H)	26.2	3′, 5′, 6′	-	-
5′	0.97 (d, 3H, J = *6.4* Hz)	23.4	4′	3′, 4′, 6′	3′ b, 3′ *a*
6′	0.91 (d, 3H, J = *6.4* Hz)	21.4	4′	3′, 4′, 5′	2′, 3′ b
3-Me	1.38 (s, 3H)	16.6	-	2, 3, 4, 16	17*a*, 15*a*
4-Me	1.09 (br s, 3H)	19.4	-	3, 4, 5, 13	14, 16
NMe$_2$	2.93 (s, 6H)	42.1	-	2′, NMe$_2$	3′ *b*

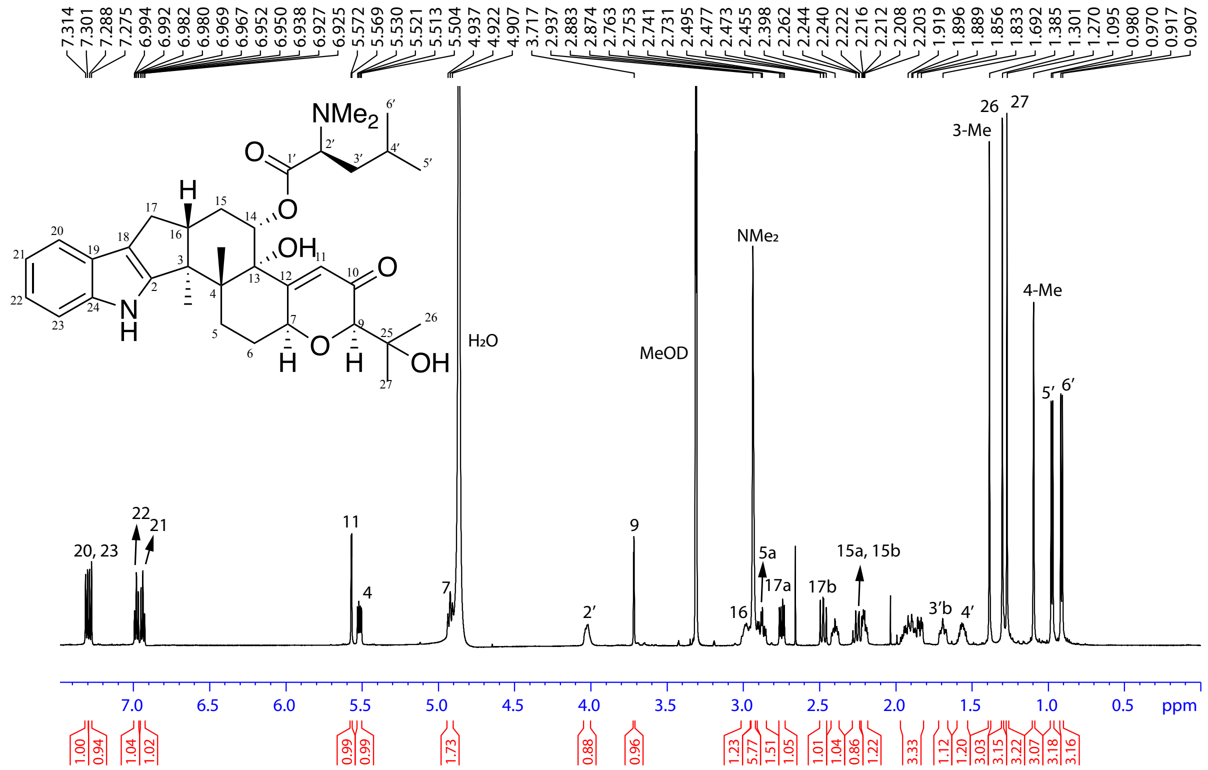

Figure S24. ^{1}H NMR (600 MHz, methanol-d_4) spectrum of noonindole C (**7**)

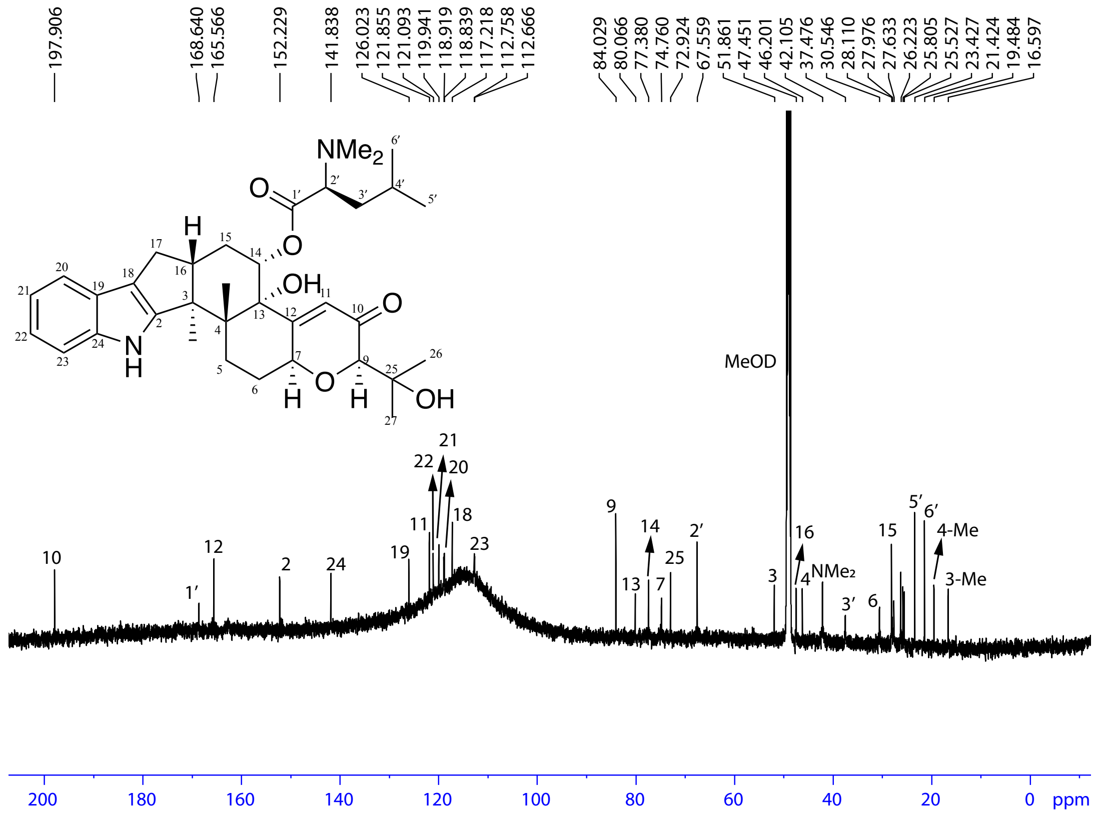

Figure S25. ^{13}C NMR (150 MHz, methanol-d_4) spectrum of noonindole C (**7**)

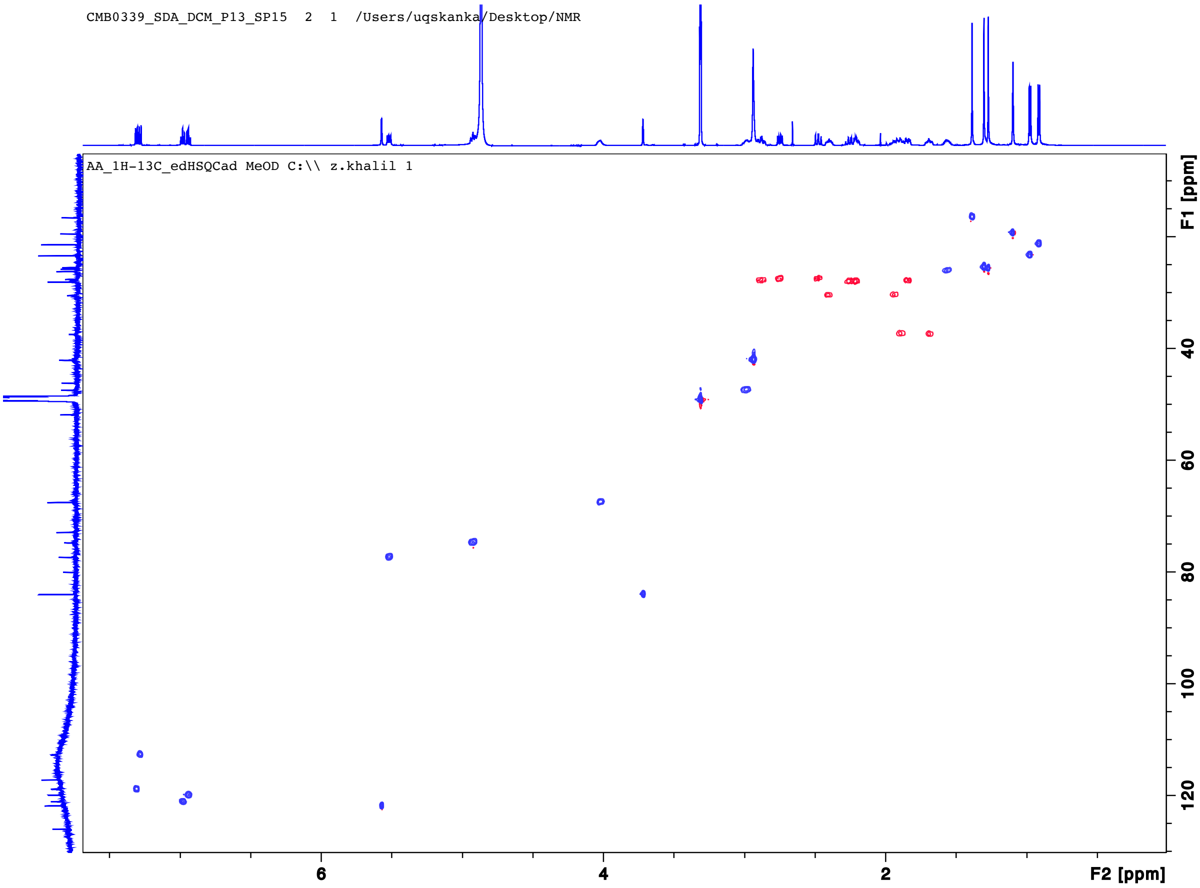

Figure S26. HSQC (methanol-d_4) spectrum of noonindole C (**7**)

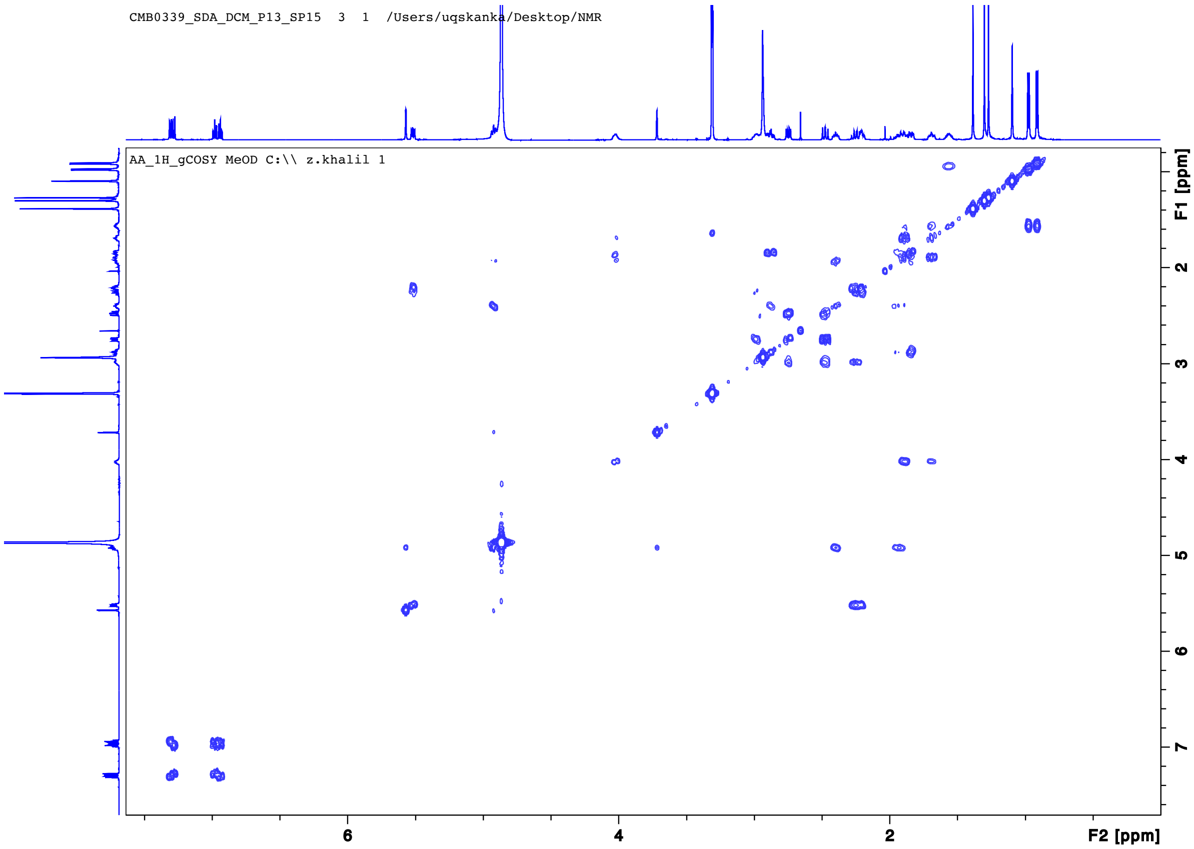

Figure S27. COSY (methanol-d_4) spectrum of noonindole C (**7**)

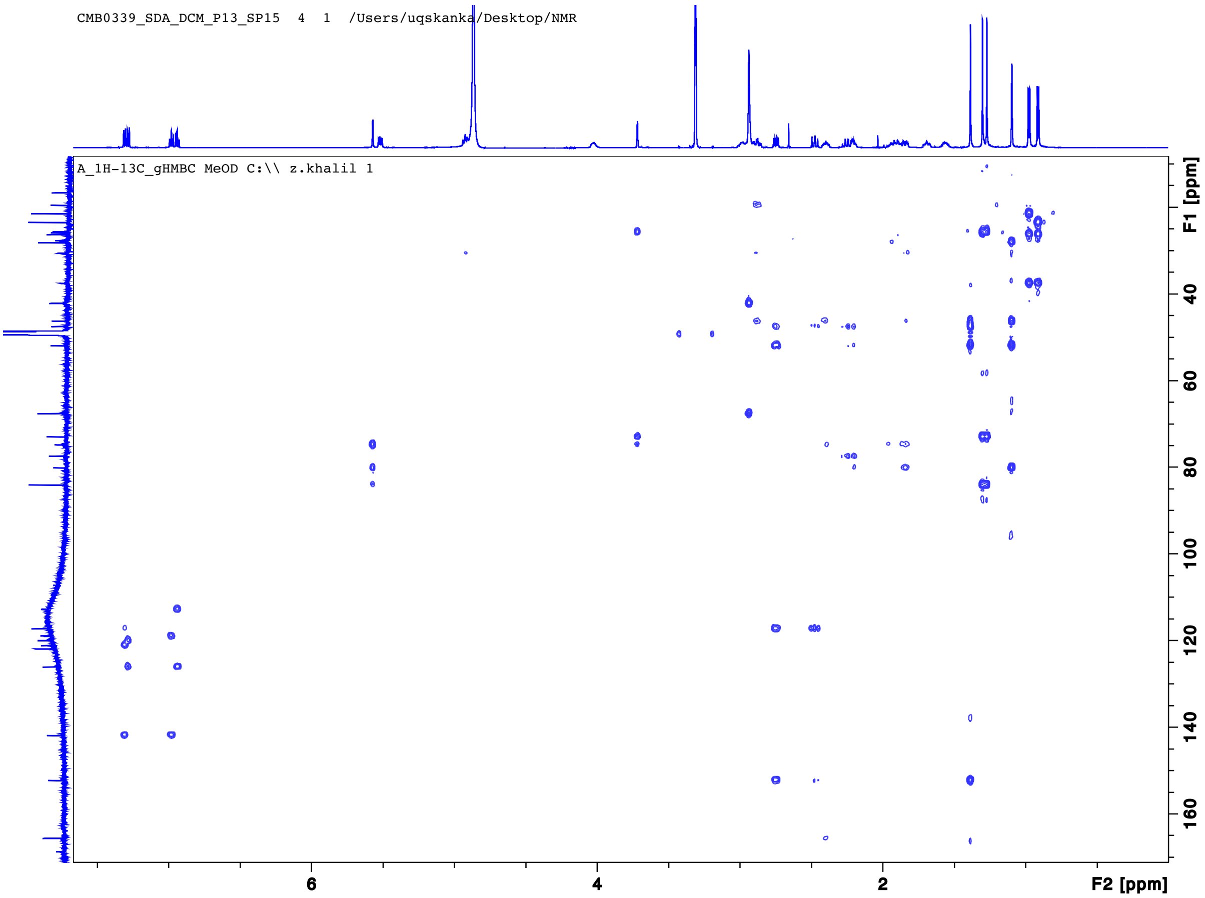

Figure S28. HMBC (methanol-d_4) spectrum of noonindole C (**7**)

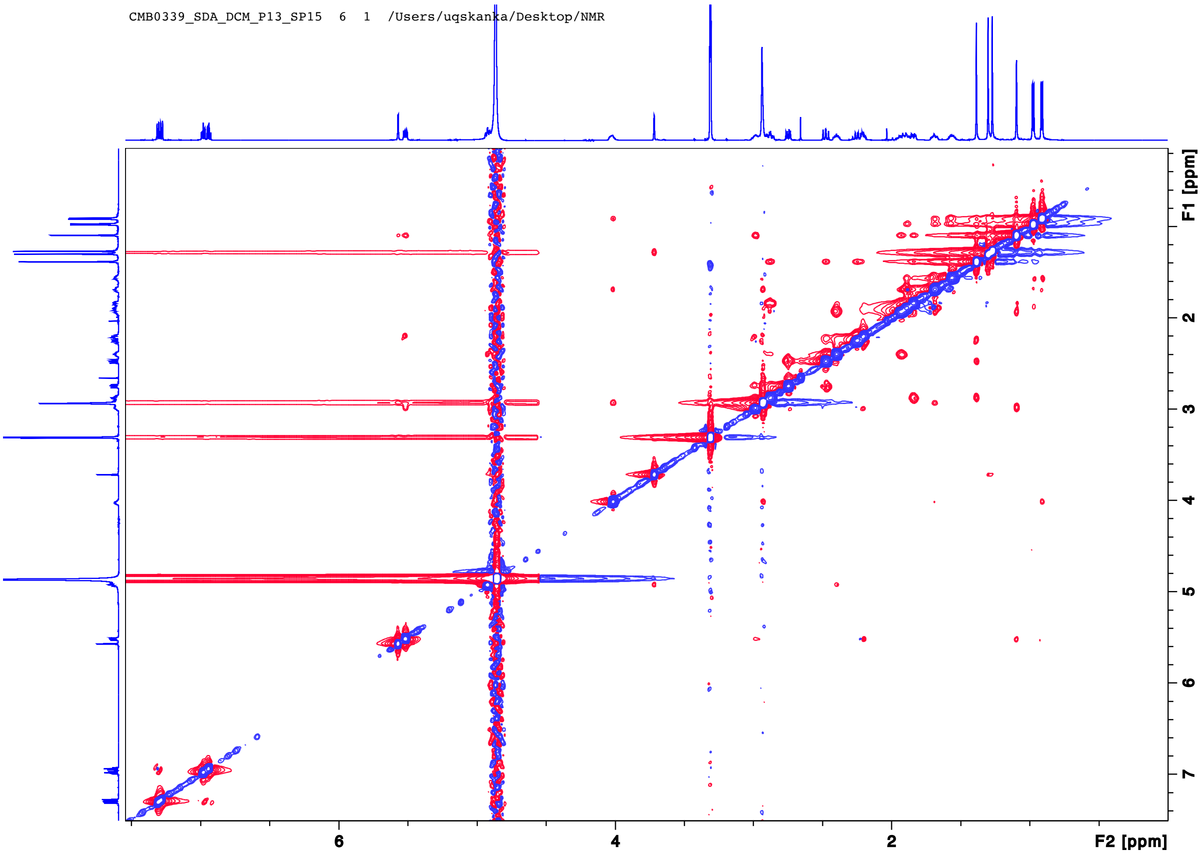

Figure S29. ROESY (methanol-d_4) spectrum of noonindole C (**7**)

Mass Spectrum Molecular Formula Report

Analysis Info

Analysis Name	D:\Data\s.kankanamge\CMB0339_SDA_DCM_P13_SP15_2.d	Acquisition Date	10/27/2020 3:48:30 PM
Method	tune-medhigh_AP.m	Operator	a.salim
Sample Name	CMB0339_SDA_DCM_P13_SP15_2	Instrument / Ser#	micrOTOF 213750.00232
Comment			

Acquisition Parameter

Source Type	ESI	Ion Polarity	Positive	Set Nebulizer	0.5 Bar
Focus	Not active			Set Dry Heater	180 °C
Scan Begin	100 m/z	Set Capillary	4500 V	Set Dry Gas	5.0 l/min
Scan End	1500 m/z	Set End Plate Offset	-500 V	Set Divert Valve	Source

Generate Molecular Formula Parameter

Formula, min.			
Formula, max.			
Measured m/z	Tolerance	Charge	
Check Valence	Minimum	Maximum	
Nirogen Rule	Electron Configuration		
Filter H/C Ratio	Minimum	Maximum	
Estimate Carbon			

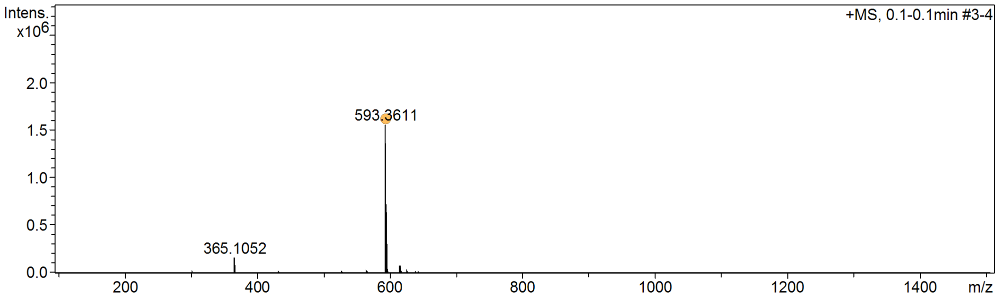

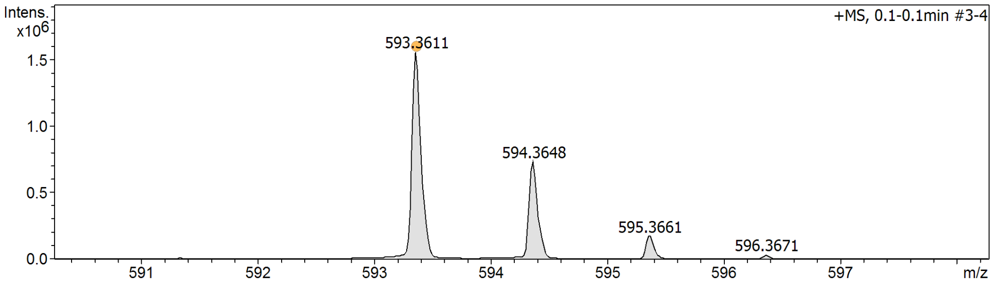

Meas. m/z	#	Ion Formula	m/z	err [ppm]	mSigma	# Sigma	Score	rdb	e⁻ Conf	N-Rule
593.3611	1	C41H45N4	593.3639	-4.6	2.7	1	43.33	21.5	even	ok
	2	C40H49O4	593.3625	2.4	14.0	2	100.00	16.5	even	ok
	3	C36H45N6O2	593.3599	-2.2	26.8	3	82.09	17.5	even	ok
	4	C35H49N2O6	593.3585	-4.4	38.1	4	22.59	12.5	even	ok
	5	C32H41N12	593.3572	-6.7	39.9	5	5.36	18.5	even	ok
	6	C31H45N8O4	593.3558	-8.9	50.8	6	0.66	13.5	even	ok
	7	C33H53O9	593.3684	12.3	51.9	7	0.02	7.5	even	ok
	8	C30H45N10O3	593.3671	10.0	53.3	8	0.23	13.5	even	ok

Bruker Compass DataAnalysis 4.1 printed: 10/27/2020 3:56:36 PM Page 1 of 2

Figure S30. HRMS spectrum of noonindole C (**7**)

7 Noonindole D (8)

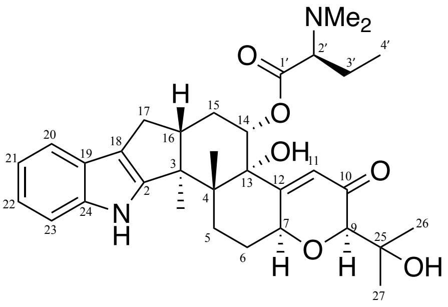

Table S5. 1D and 2D NMR (methanol-d_4, 600 MHz) data for noonindole D (**8**)

Pos.	$\delta_{H,}$ mult. (*J* in Hz)	δ_C	COSY	^{1}H-^{13}C HMBC	ROESY
1-NH	-	-	-	-	-
2	-	151.1	-	-	-
3	-	50.5	-	-	-
4	-	44.6	-	-	-
5	*a* 2.87[a] (m, 1H)	28.0	-	-	-
	b 1.80, (m, 1H)	28.0	5*a*[a], 6*b*	-	-
6	*a* 2.37 (m, 1H)	30.4	5*a*[a], 6*b*, 7	-	-
	b 1.91 (m, 1H)	30.4	5*b*, 6*a*	-	-
7	4.90 (m, 1H)	74.6	6*a*, 6*b*, 9, 11	-	-
9	3.71 (d, 1H, J = *1.9* Hz)	83.7	7	25	27
10	-	nd	-	-	-
11	5.58 (d, 1H, J = *1.9* Hz)	121.7	7	7, 9, 13	14
12	-	nd	-	-	-
13	-	78.9	-	-	-
14	5.37 (dd, 1H, J = *10.6, 5.1* Hz)	74.4	15*a*, 15*b*	-	11, 16, 4-Me
15	*a* 2.16 (m, 1H)	28.4	14, 15*b*, 16	-	-
	b 2.04 (ddd, 1H, J = *7.1, 5.1, 2.3* Hz)	28.4	15*a*, 14, 16	-	-
16	2.95 (m, 1H)	47.0	15*a*, 17*a*, 17*b*	-	14, 4-Me
17	*a* 2.72 (dd, 1H, J = *13.1, 6.4* Hz)	27.5	16, 17*b*	2, 3, 18	-
	b 2.45 (dd, 1H, J = *13.1, 10.7* Hz)	27.5	17*a*, 16	18	-
18	-	116.8	-	-	-
19	-	124.7	-	-	-
20	7.30 (d, 1H, J = *7.7* Hz)	118.6	21	18, 22, 24	-
21	6.92 (ddd, 1H, J = *7.7, 7.3, 0.9* Hz)	119.6	20, 22	19, 23	-
22	6.97 (ddd, 1H, J = *8.1, 7.3, 0.9* Hz)	120.7	21, 23	20, 24	-
23	7.27 (d, 1H, J = *8.1* Hz)	112.4	22	19, 21	-
24	-	140.4	-	-	-
25	-	71.6	-	-	-
26	1.28 (s, 3H)	25.1	-	9, 25, 27	-
27	1.26 (s, 3H)	25.9	-	9, 25, 26	9
1′	-	nd	-	-	-
2′	2.87[a] (m, 1H)	72.9	-	-	-
3′	*a* 1.77 (m, 1H)	23.8	2′[a], 4′	-	-
	b 1.69 (m, 1H)		2′[a], 4′	-	-
4′	0.91 (t, 1H, J = *7.4* Hz)	10.1	3′*a*, 3′*b*	2′, 3′	-
3-Me	1.37 (s, 3H)	16.3	-	2, 3, 4, 16	-
4-Me	1.08 (s, 3H)	19.4	-	3, 4, 5, 13	14, 16
NMe_2	2.26 (s, 6H)	43.4	-	2′, NMe_2	-

[nd] not detected

[a] Resonances with the same superscript within a column are overlapping and assignments may be interchanged

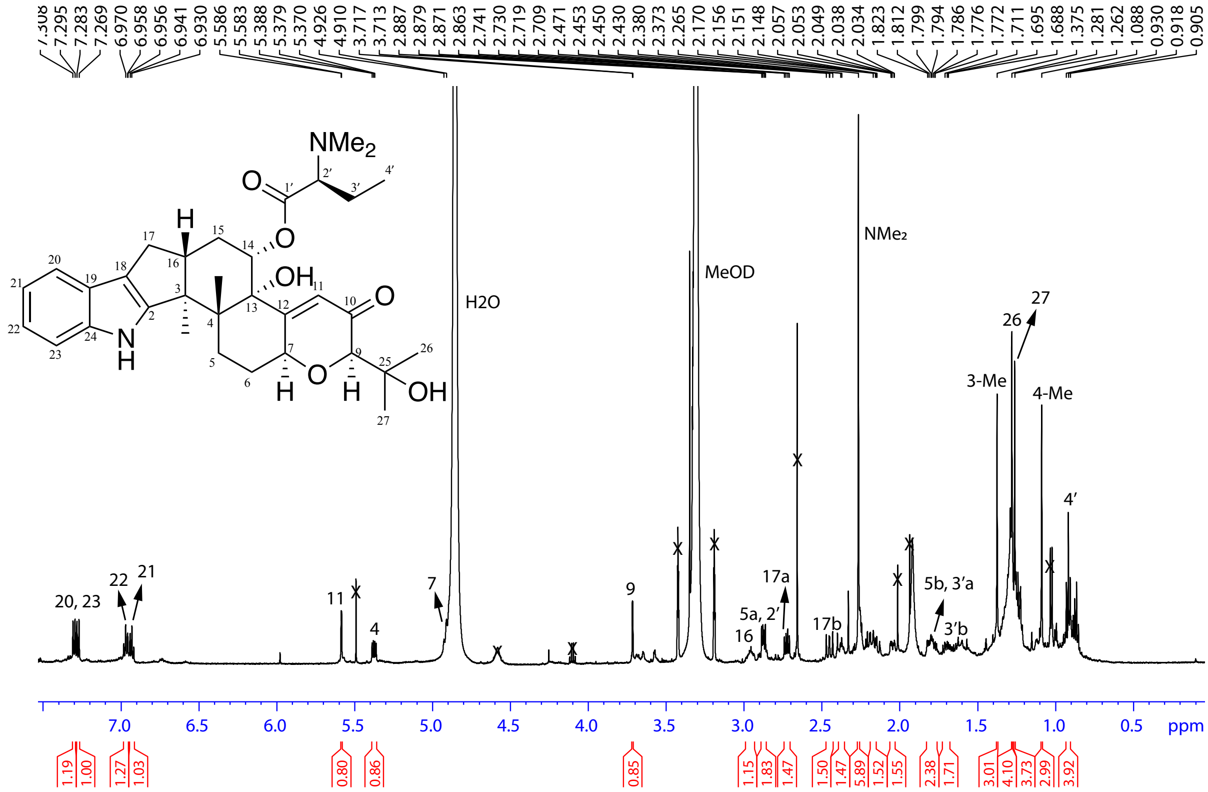

Figure S31. ^{1}H NMR (methanol-*d*4) spectrum of noonindole D (**8**)

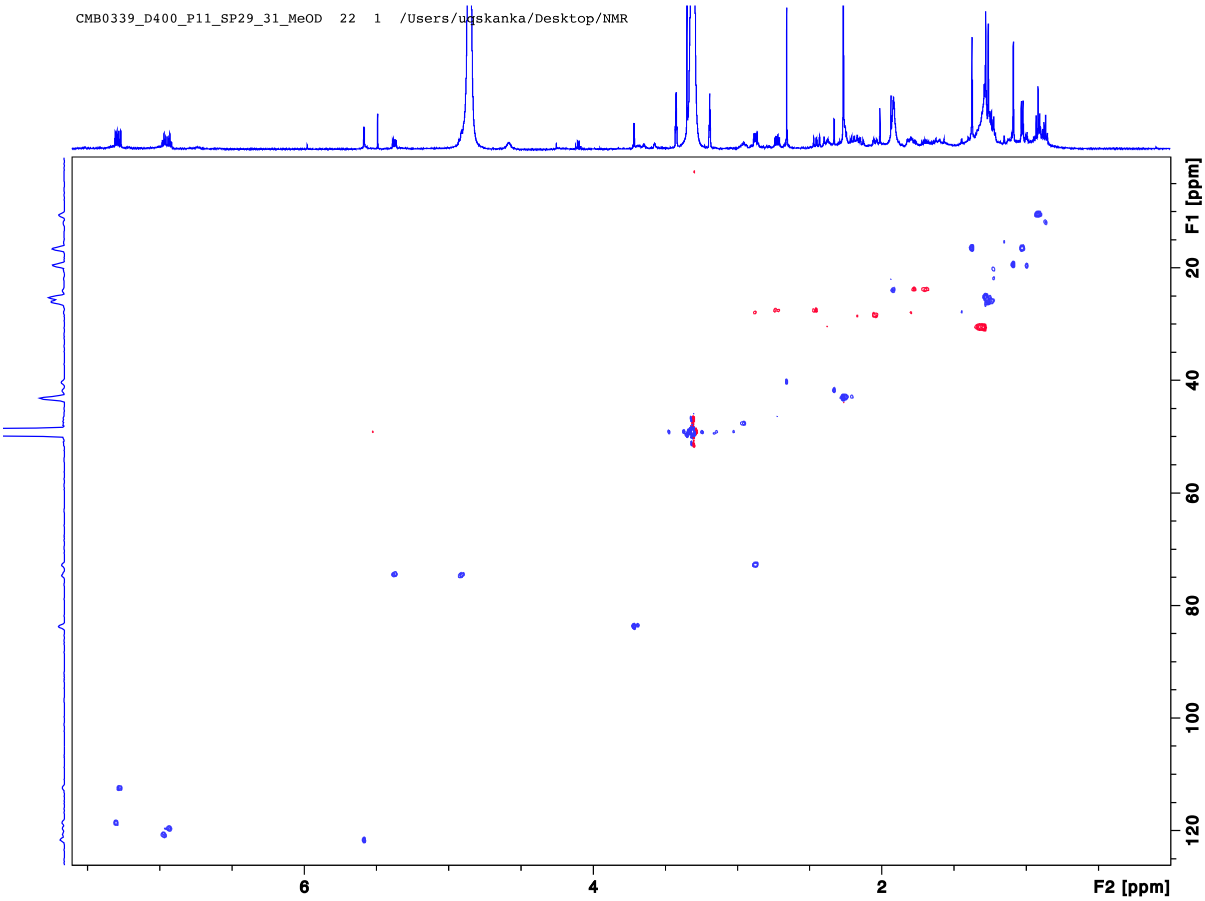

Figure S32. HSQC (methanol-*d*4) spectrum of noonindole D (**8**)

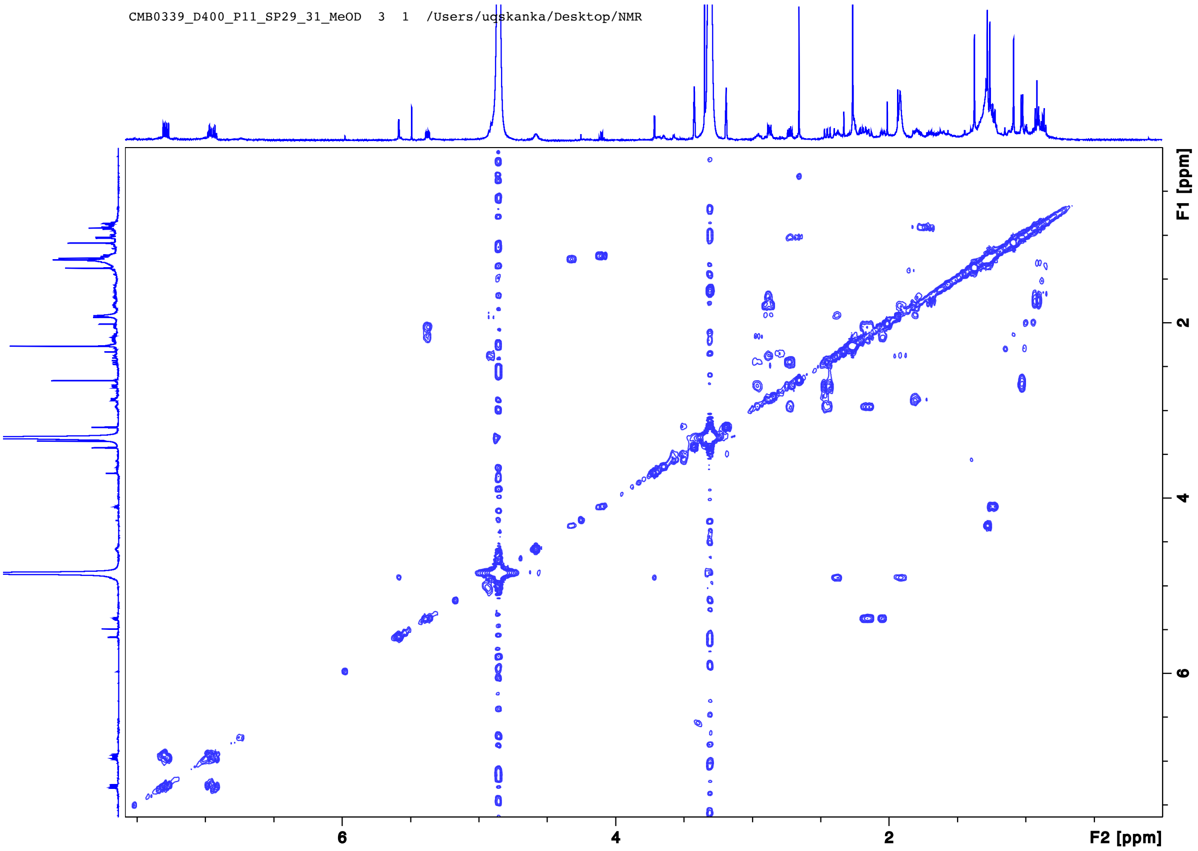

Figure S33. COSY (methanol-*d*4) spectrum of noonindole D (**8**)

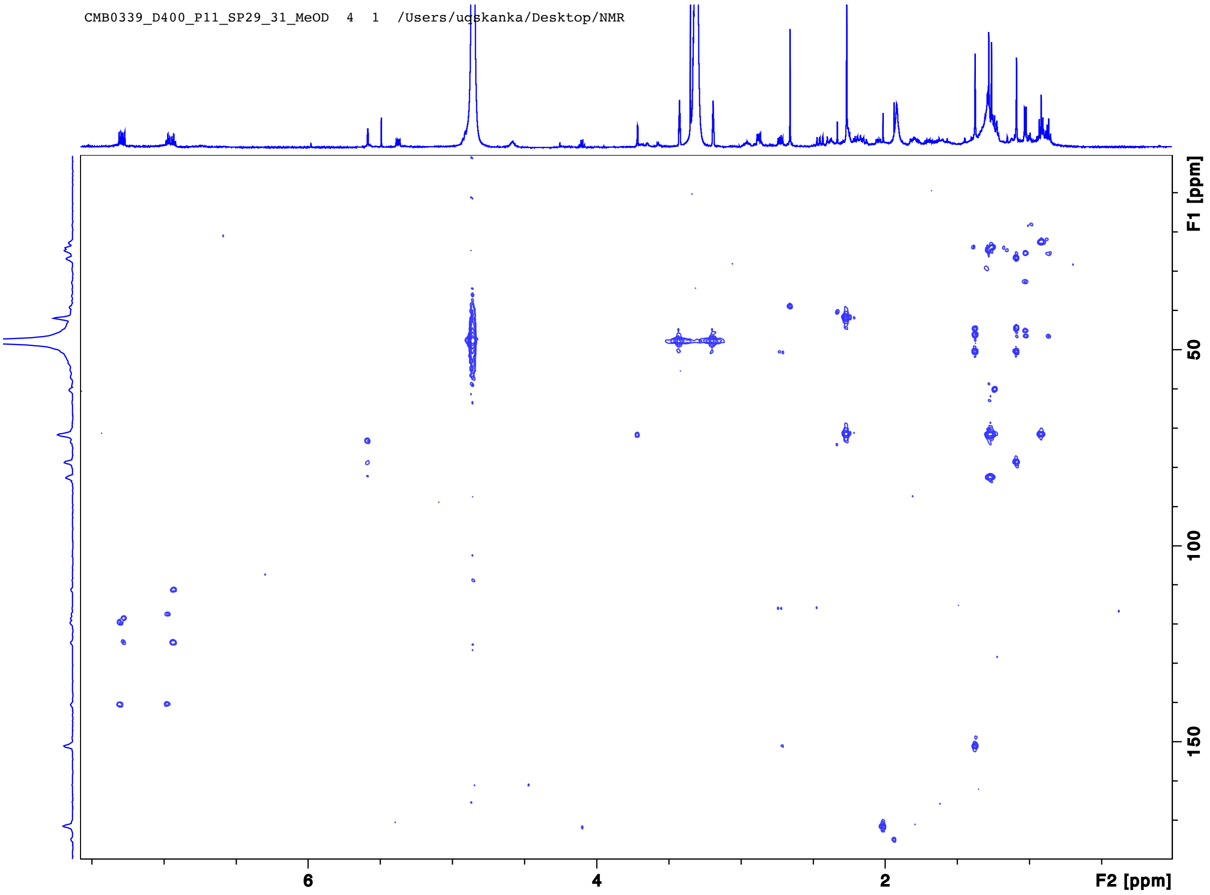

Figure S34. HMBC (methanol-d_4) spectrum of noonindole D (**8**)

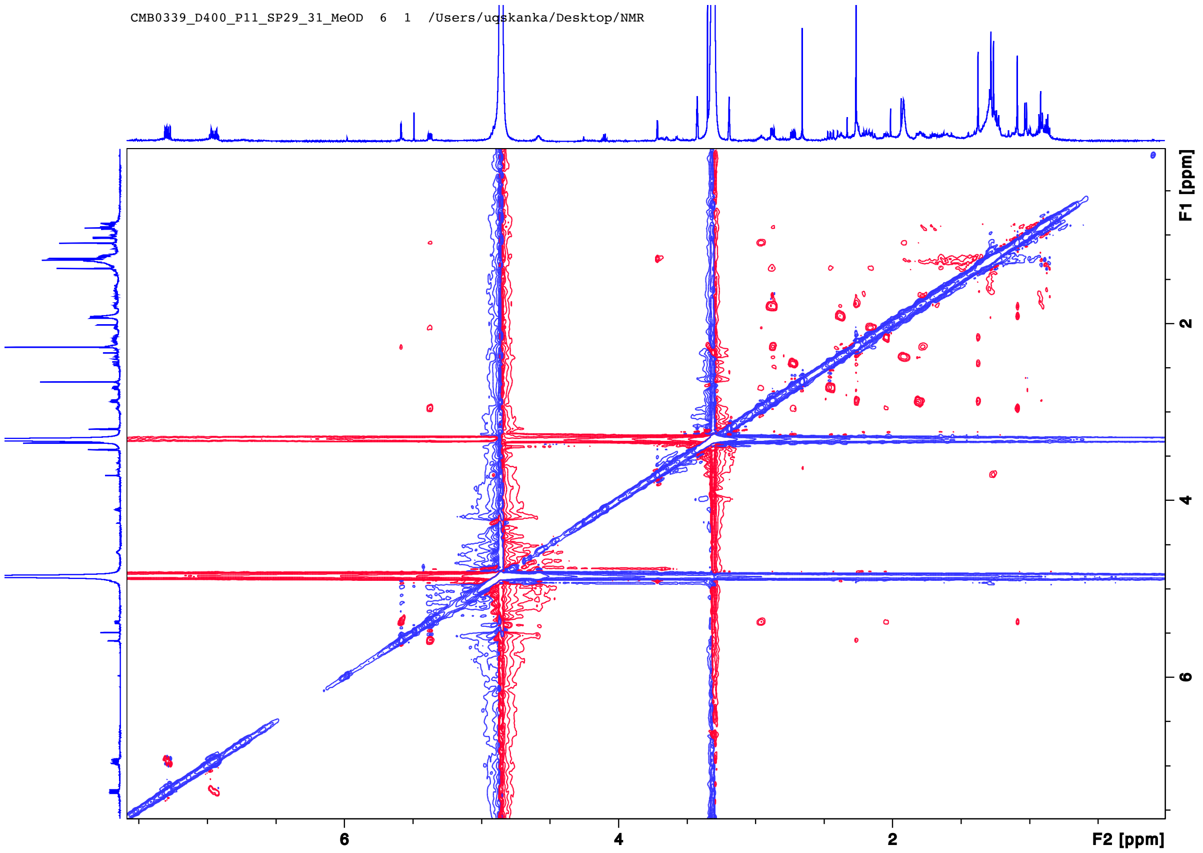

Figure S35. ROESY (methanol-d_4) spectrum of noonindole D (**8**)

Mass Spectrum Molecular Formula Report

Analysis Info

Analysis Name	D:\Data\s.kankanamge\CMB0339_D400_P11_SP29_31_again.d	Acquisition Date	11/2/2021 1:56:53 PM
Method	tune-med_AP.m	Operator	a.salim
Sample Name	CMB0339_D400_P11_SP29_31_again	Instrument / Ser#	micrOTOF 213750.00232
Comment			

Acquisition Parameter

Source Type	ESI	Ion Polarity	Positive	Set Nebulizer	0.8 Bar
Focus	Not active			Set Dry Heater	180 °C
Scan Begin	100 m/z	Set Capillary	4500 V	Set Dry Gas	5.0 l/min
Scan End	1000 m/z	Set End Plate Offset	-500 V	Set Divert Valve	Source

Generate Molecular Formula Parameter

Formula, min.		
Formula, max.		
Measured m/z	Tolerance	Charge
Check Valence	Minimum	Maximum
Nirogen Rule	Electron Configuration	
Filter H/C Ratio	Minimum	Maximum
Estimate Carbon		

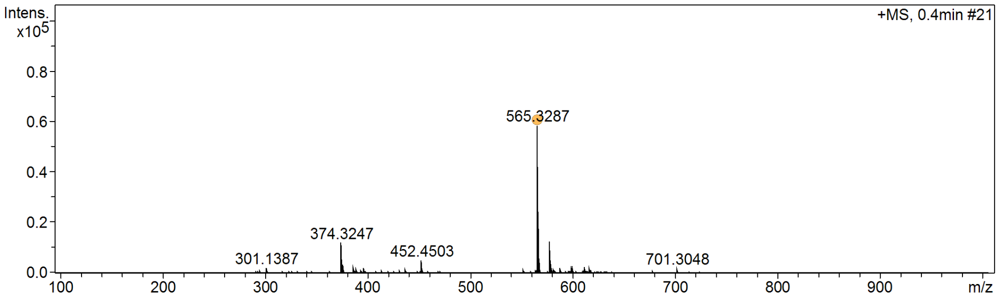

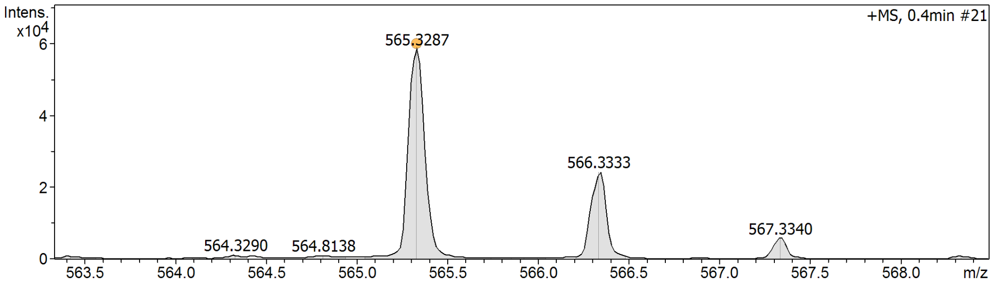

Meas. m/z	#	Ion Formula	m/z	err [ppm]	mSigma	# Sigma	Score	rdb	e⁻ Conf	N-Rule
565.3287	1	C38H45O4	565.3312	-4.5	3.7	1	25.45	16.5	even	ok
	2	C39H41N4	565.3326	-6.9	13.5	2	5.45	21.5	even	ok
	3	C34H41N6O2	565.3286	-0.2	13.9	3	100.00	17.5	even	ok
	4	C33H45N2O6	565.3272	-2.6	23.7	4	39.72	12.5	even	ok
	5	C30H37N12	565.3259	-5.0	26.6	5	12.80	18.5	even	ok

Bruker Compass DataAnalysis 4.1 printed: 11/2/2021 2:05:05 PM Page 1 of 1

Figure S36. HRMS spectrum of noonindole D (**8**)

8 Noonindole E (9)

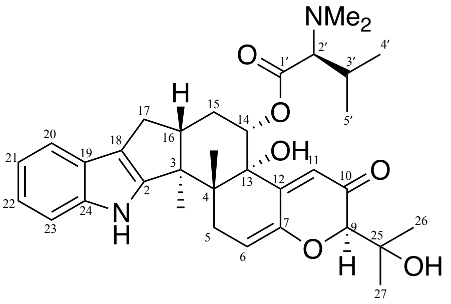

Table S6. 1D and 2D NMR (methanol-d_4, 600 MHz) data for noonindole E (**9**)

Pos.	δ_H, mult. (J in Hz)	δ_C	COSY	^{1}H-^{13}C HMBC	ROESY
1-NH	-	-	-	-	-
2	-	152.5	-	-	-
3	-	51.9	-	-	-
4	-	46.2	-	-	-
5	*a* 3.28*	33.0	5*b*, 6	6, 7, 4-Me	-
	b 2.36 (d, 1H, J = *6.3* Hz)		5*a*, 6	6, 7	-
6	5.74 (m, 1H)	113.5	5*a*, 5*b*	-	-
7	-	146.4	-	-	-
9	4.09 (s, 1H)	87.6	-	7, 26[a], 27[a], 10	26, 27
10	-	196.8	-	-	-
11	5.71 (br s, 1H)	119.5	-	7, 9, 13	14
12	-	nd	-	-	-
13	-	77.8	-	-	-
14	5.41 (dd, 1H, J = *9.8, 5.8* Hz)	75.5	15	-	11, 16, 4-Me
15	2.18 (m, 1H)	30.0	14, 16	3, 13, 16	-
16	2.90 (m, 1H)	47.5	15, 17*a*, 17*b*	-	14, 4-Me
17	*a* 2.72 (dd, 1H, J = *13.0, 6.5* Hz)	28.0	16, 17*b*	2, 3, 18	-
	b 2.44 (dd, 1H, J = *13.0, 10.8* Hz)		16, 17*a*	18	-
18	-	117.4	-	-	-
19	-	126.2	-	-	-
20	7.30 (d, 1H, J = *7.2* Hz)	119.1	21	18, 22, 24	-
21	6.93 (m, 1H)	120.0	20, 22	19, 23	-
22	6.97 (m, 1H)	121.2	21, 23	20, 24	-
23	7.27 (d, 1H, J = *7.5* Hz)	112.8	22	19, 21	-
24	-	142.1	-	-	-
25	-	75.6	-	-	-
26	1.33 (s, 3H)	27.1[a]	-	9, 25, 27[a]	9
27	1.26 (s, 3H)	27.1[a]	-	9, 25, 26[a]	9
1′	-	nd	-	-	-
2′	2.86 (d, 1H, J = *9.4* Hz)	76.0	3′	NMe_2	-
3′	2.11 (m, 1H)	28.7	2′, 4′/5′	-	-
4′/5′	0.97 (t, 6H, J = *7.4* Hz)	19.9	3′	2′, 3′, 4′/5′	-
3-Me	1.40 (s, 3H)	16.9	-	2, 3, 4, 16	-
4-Me	1.14 (s, 3H)	21.0	-	3, 4, 5, 13	14, 16
NMe_2	2.34 (s, 6H)	42.4	-	2′, NMe_2	-

[a] Resonances with the same superscript within a column are overlapping and assignments may be interchanged
*Obscured by residual MeOD signal
[nd] not detected

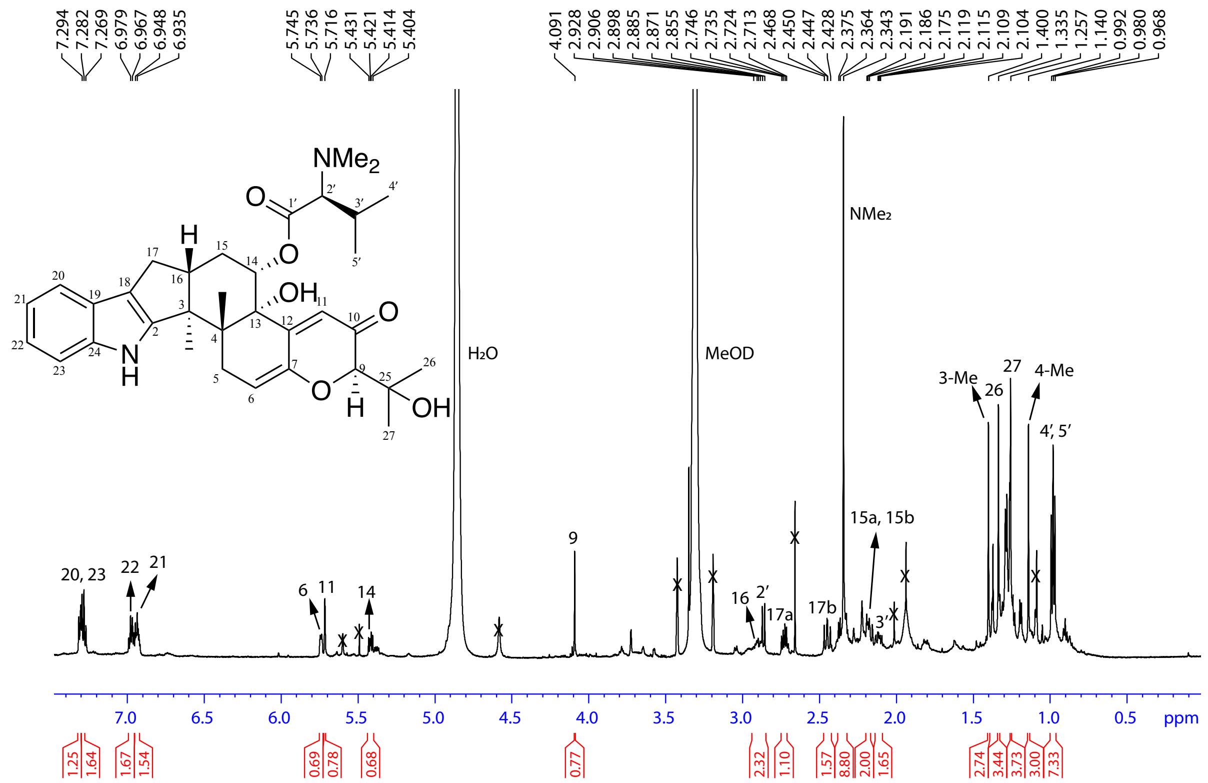

Figure S37. ^{1}H NMR (methanol-d_4) spectrum of noonindole E (**9**)

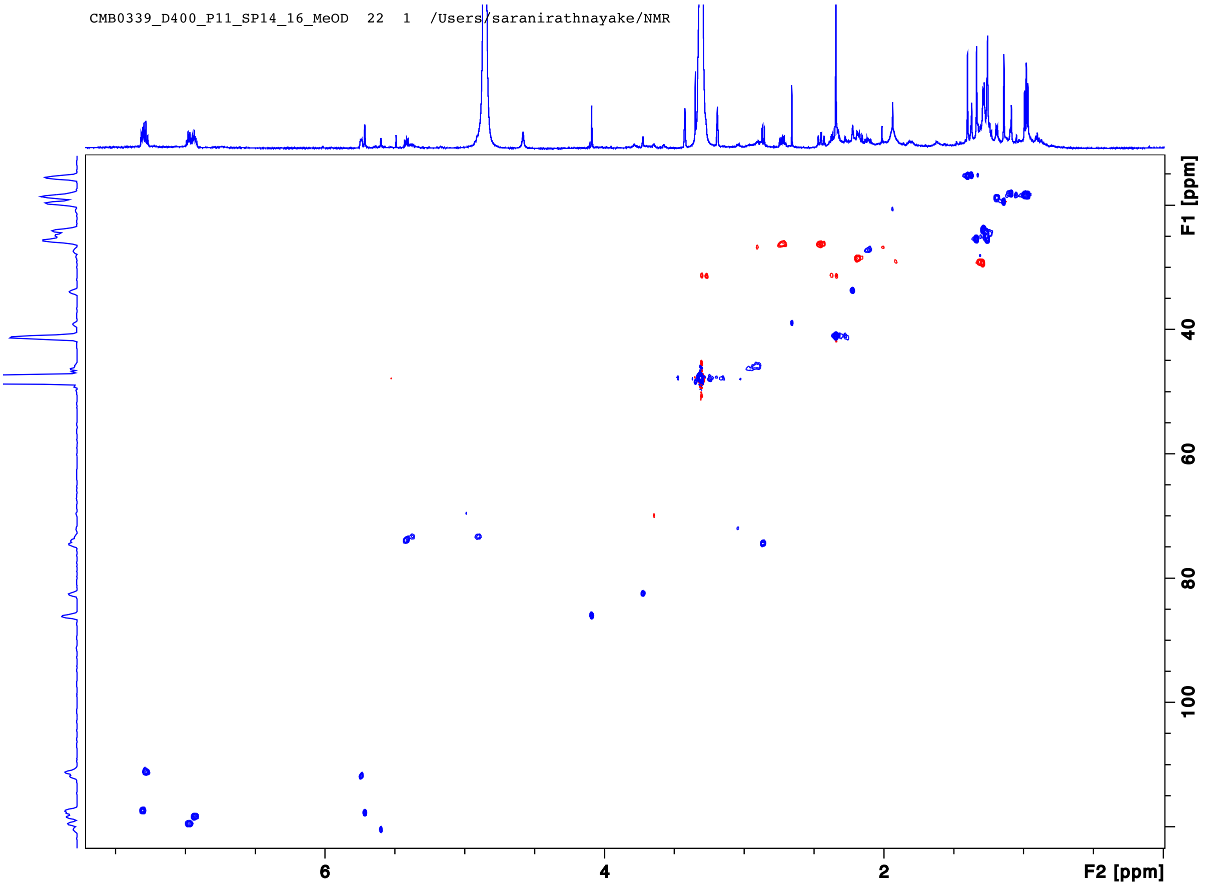

Figure S38. HSQC (methanol-d_4) spectrum of noonindole E (**9**)

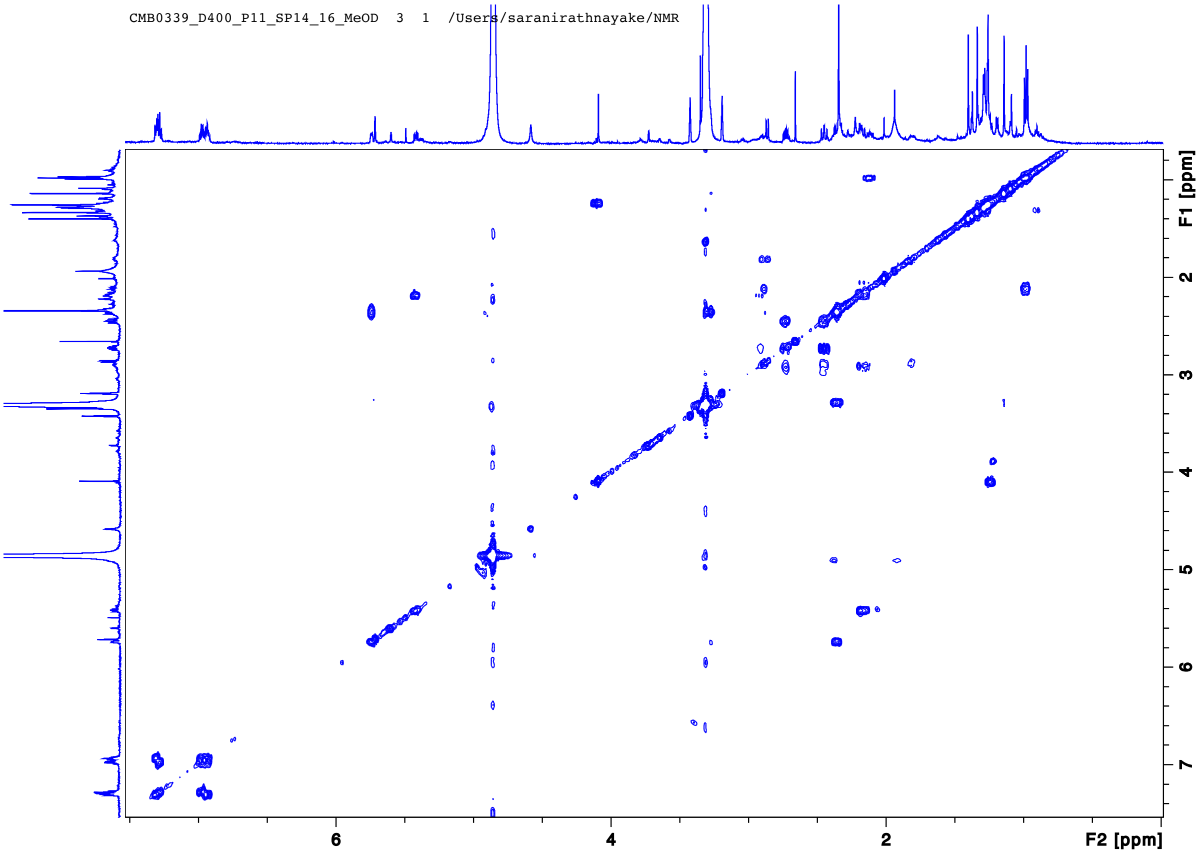

Figure S39. COSY (methanol-d_4) spectrum of noonindole E (**9**)

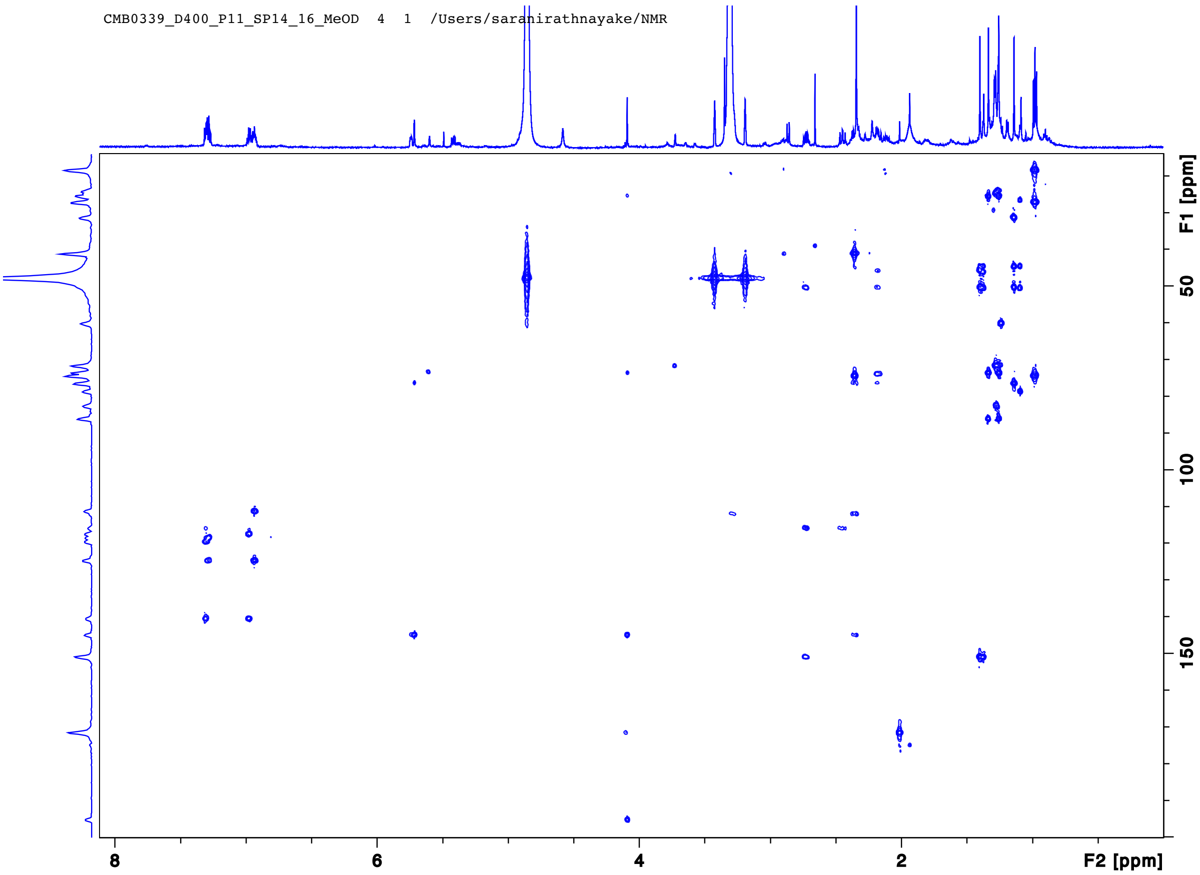

Figure S40. HMBC (methanol-d_4) spectrum of noonindole E (**9**)

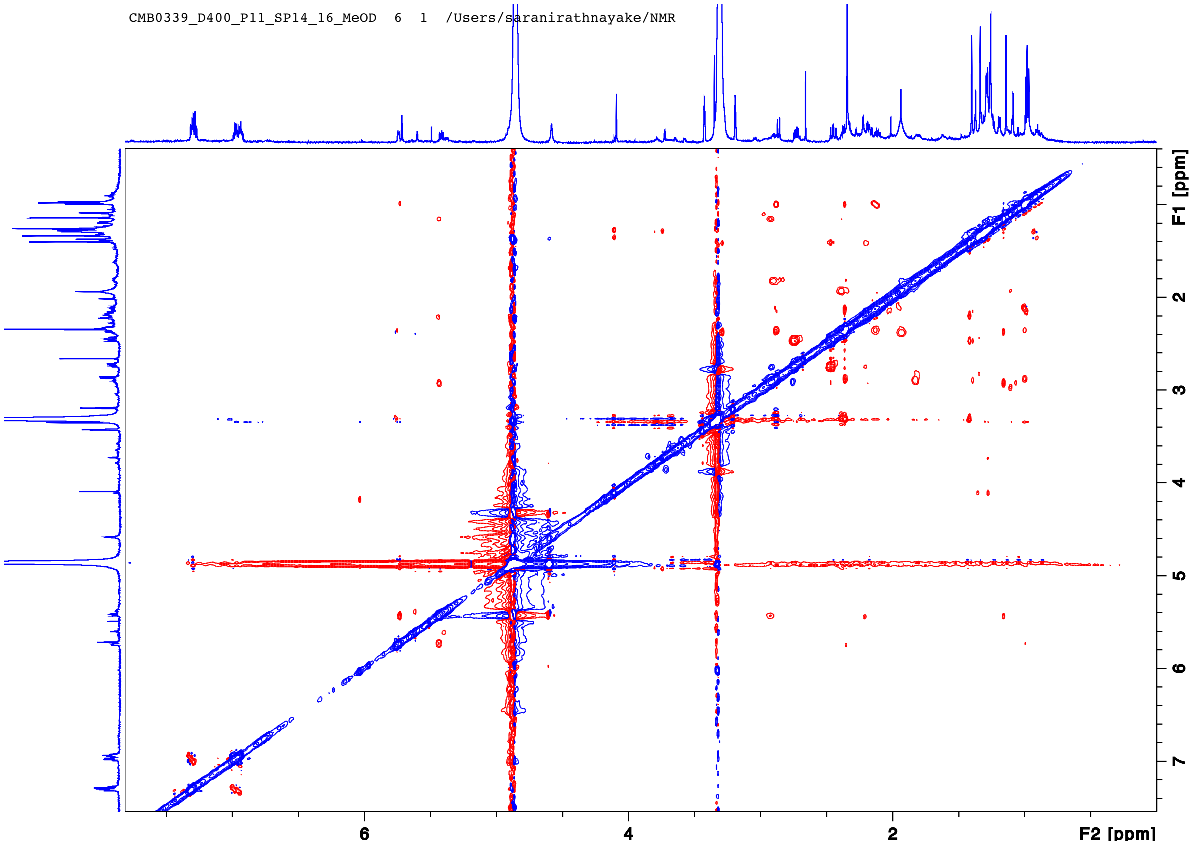

Figure S41. ROESY (methanol-d_4) spectrum of noonindole E (**9**)

Mass Spectrum Molecular Formula Report

Analysis Info

Analysis Name	D:\Data\s.kankanamge\CMB0339_D400_P11_SP14_16_again.d	Acquisition Date	11/2/2021 2:06:04 PM
Method	tune-med_AP.m	Operator	a.salim
Sample Name	CMB0339_D400_P11_SP14_16_again	Instrument / Ser#	micrOTOF 213750.00232
Comment			

Acquisition Parameter

Source Type	ESI	Ion Polarity	Positive	Set Nebulizer	0.8 Bar
Focus	Not active			Set Dry Heater	180 °C
Scan Begin	100 m/z	Set Capillary	4500 V	Set Dry Gas	5.0 l/min
Scan End	1000 m/z	Set End Plate Offset	-500 V	Set Divert Valve	Source

Generate Molecular Formula Parameter

Formula, min.		
Formula, max.		
Measured m/z	Tolerance	Charge
Check Valence	Minimum	Maximum
Nirogen Rule	Electron Configuration	
Filter H/C Ratio	Minimum	Maximum
Estimate Carbon		

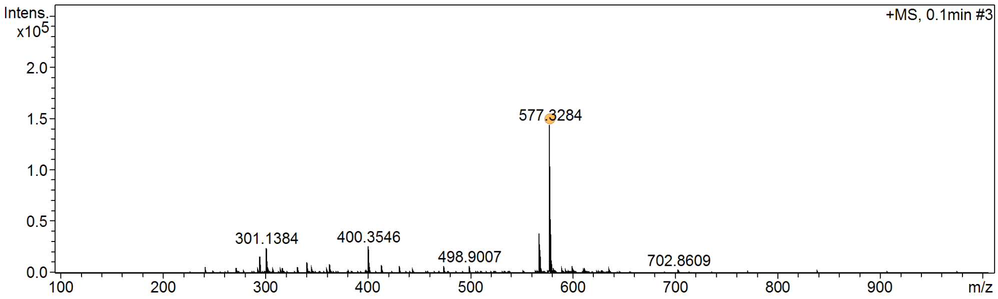

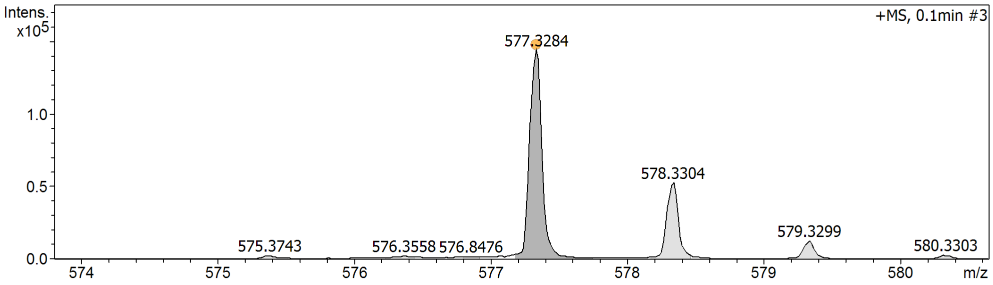

Meas. m/z	#	Ion Formula	m/z	err [ppm]	mSigma	# Sigma	Score	rdb	e⁻ Conf	N-Rule
577.3284	1	C30H41N8O4	577.3245	-6.7	8.0	1	7.33	14.5	even	ok
	2	C34H45N2O6	577.3272	-2.0	9.0	2	74.87	13.5	even	ok
	3	C31H37N12	577.3259	4.4	12.1	3	26.28	19.5	even	ok
	4	C35H41N6O2	577.3286	0.3	20.7	4	100.00	18.5	even	ok
	5	C39H45O4	577.3312	4.9	32.5	5	12.57	17.5	even	ok

Bruker Compass DataAnalysis 4.1 printed: 11/2/2021 2:10:47 PM Page 1 of 1

Figure S42. HRMS spectrum of noonindole E (**9**)

9 Noonindole F (10)

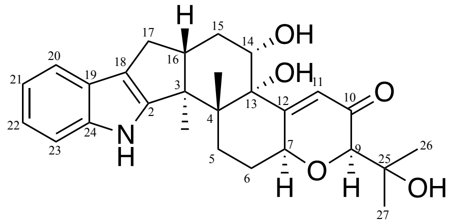

Table S7. 1D and 2D NMR (in methanol-d_4, 600 MHz) data for noonindole F (**10**)

Pos.	$\delta_{H,}$ mult. (*J* in Hz)	δ_C	COSY	^{1}H-^{13}C HMBC	ROESY
1-NH	-	-	-	-	-
2	-	152.9	-	-	-
3	-	51.8	-	-	-
4	-	44.7	-	-	-
5	*a* 2.80 (ddd, 1H, J = *13.5,13.5, 5.1* Hz)	28.1	5*b*, 6*a*	4, 6, 4-Me	3-Me
	b 1.79 (dd, 1H, J = *13.5, 5.1* Hz)		5*a*, 6*b*	6, 7, 13	-
6	*a* 2.33 (m, 1H)	30.2	5*a*, 6*b*, 7	7, 5, 4, 12	3-Me
	b 1.90 (m, 1H)		6*a*, 5*b*, 7	5, 7	-
7	4.88 (m, 1H)	74.7	6*a*, 6*b*, 9, 11	11, 12	9
9	3.78 (d, 1H, J = *1.5* Hz)	84.0	7	7, 25, 26, 27	7, 26, 27
10	-	200.0	-	-	-
11	6.01 (br s, 1H)	122.9	7	7, 9, 13	14
12	-	167.5	-	-	-
13	-	80.8	-	-	-
14	4.16 (dd, 1H, J = *10.5, 4.9* Hz)	70.3	15*a*, 15*b*	-	4-Me, 16, 11
15	*a* 2.08 (dd, 1H, J = *13.3, 10.5* Hz)	31.7	14, 15*b*, 16	14, 16	-
	b 1.93 (m, 1H)		15*a*, 14	14, 16	4-Me
16	2.90 (m, 1H)	48.0	15*a*, 15*b*, 17*a*, 17*b*	3-Me	14, 4-Me
17	*a* 2.68 (dd, 1H, J = *13.0, 6.1* Hz)	27.8	16, 17*b*	2, 3, 16, 18	-
	b 2.42 (dd, 1H, J = *13.0, 10.0* Hz)		16, 17*a*	16, 18	-
18	-	117.4	-	-	-
19	-	126.2	-	-	-
20	7.29 (d, 1H, J = *7.6* Hz)	118.8	21	18, 22, 24	-
21	6.92 (br dd, 1H, J = *7.6, 7.2* Hz)	119.8	20, 22	19, 23	-
22	6.95 (br dd, 1H, J = *7.4, 7.2* Hz)	120.9	21, 23	20, 24	-
23	7.26 (d, 1H, J = *7.4* Hz)	112.7	22	19, 21	-
24	-	141.8	-	-	-
25	-	73.2	-		-
26	1.29 (s, 3H)	25.2	-	9, 25, 27	9
27	1.27 (s, 3H)	26.2	-	9, 25, 26	9
3-Me	1.32 (s, 3H)	16.5	-	2, 3, 4, 16	5*a*, 6*a*
4-Me	1.04 (s, 3H)	19.6	-	3, 4, 5, 13	14, 15b, 16

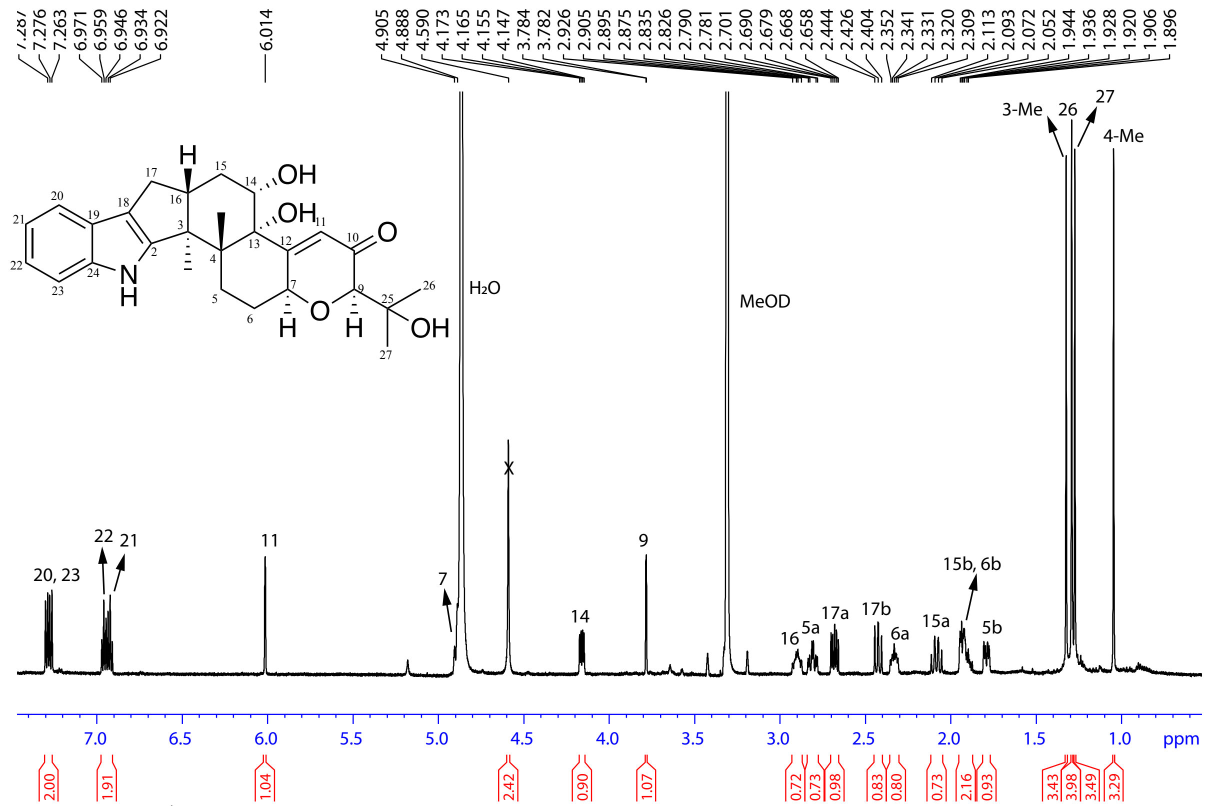

Figure S43. ^{1}H NMR (methanol-d_4) spectrum of noonindole F (**10**)

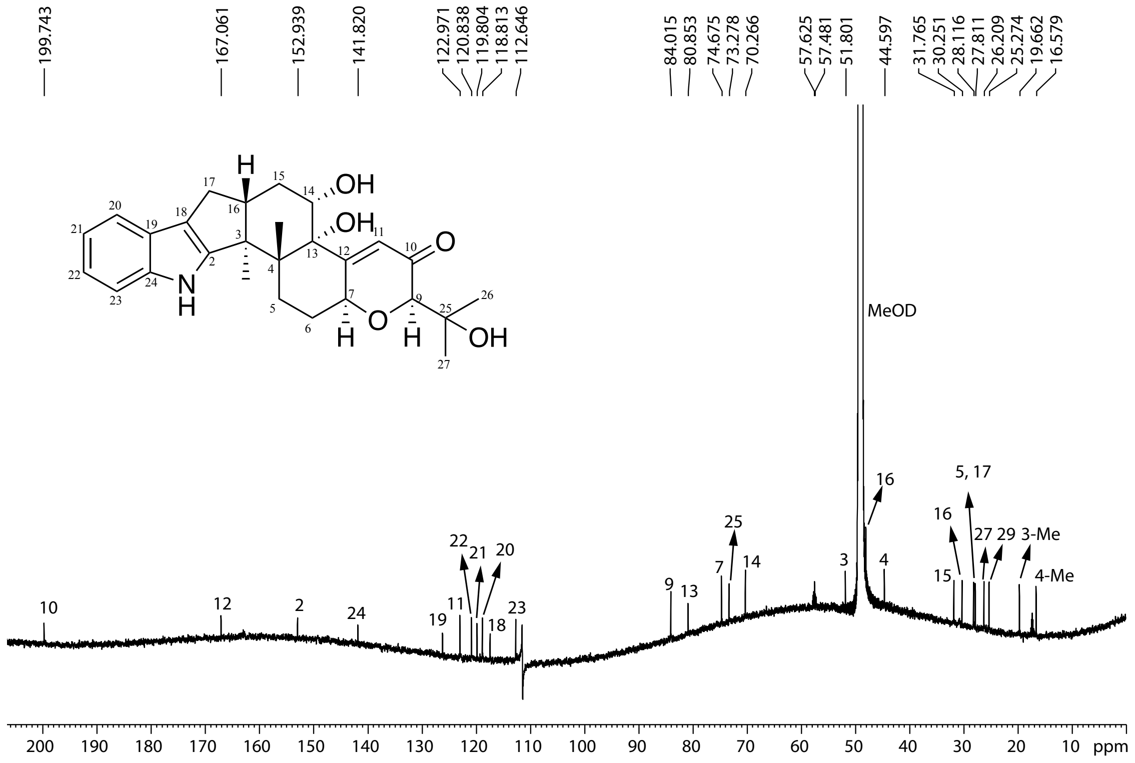

Figure S44. ^{13}C NMR (methanol-d_4) spectrum of noonindole F (**10**)

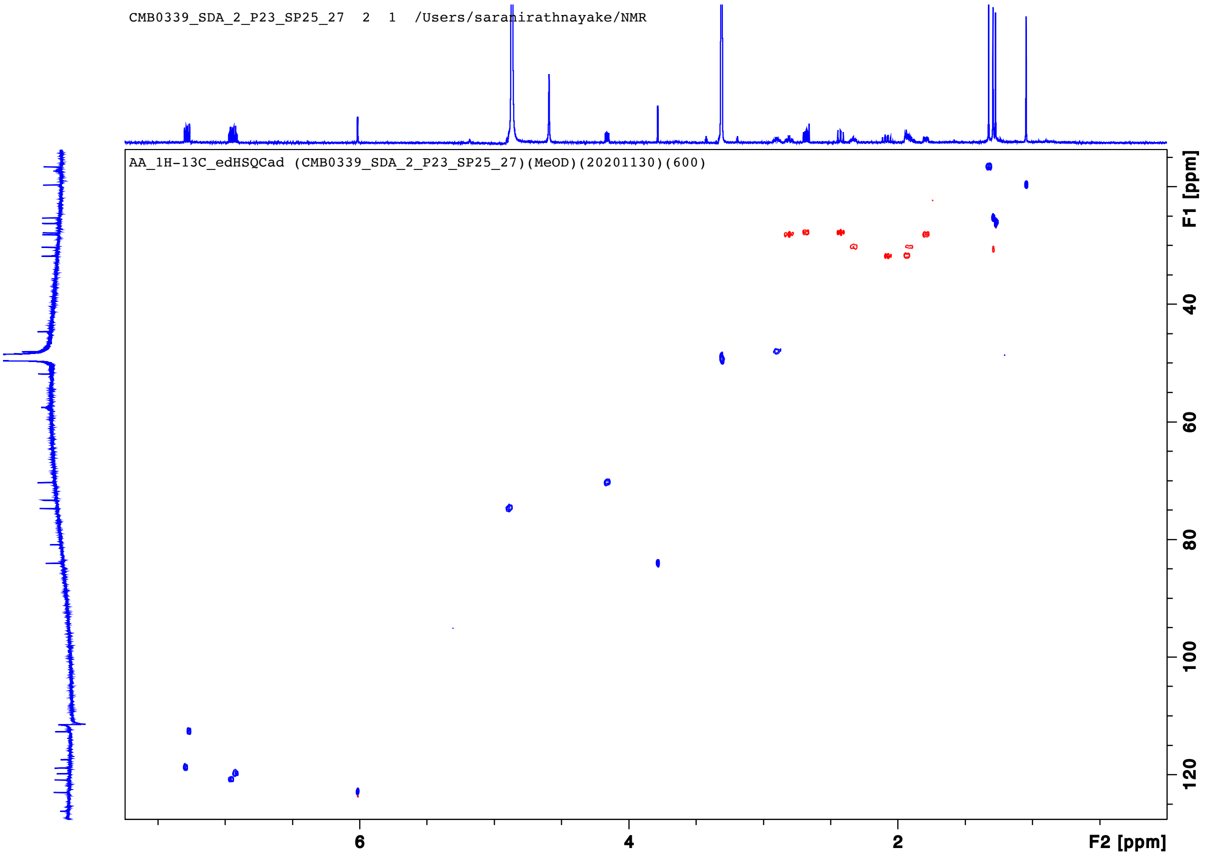

Figure S45. HSQC (methanol-d_4) spectrum of compound noonindole F (**10**)

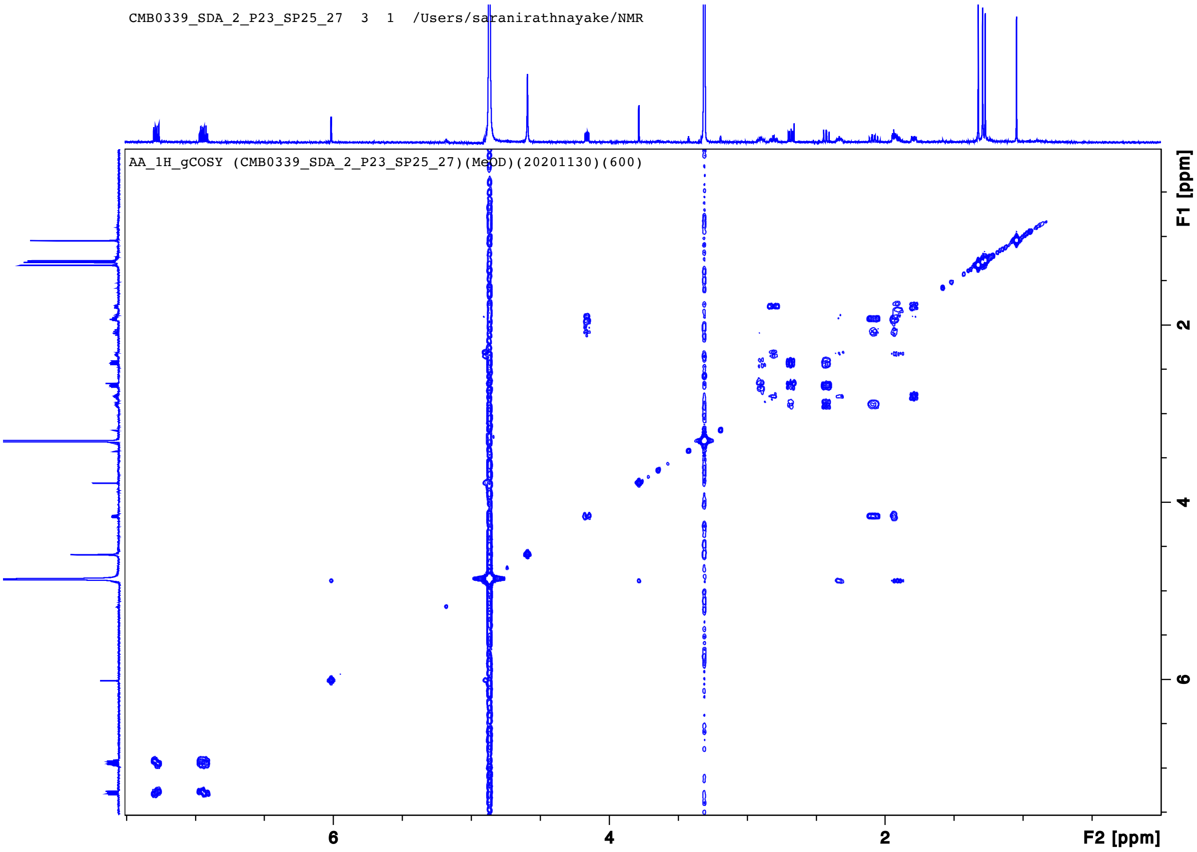

Figure S46. COSY (methanol-d_4) spectrum of compound noonindole F (**10**)

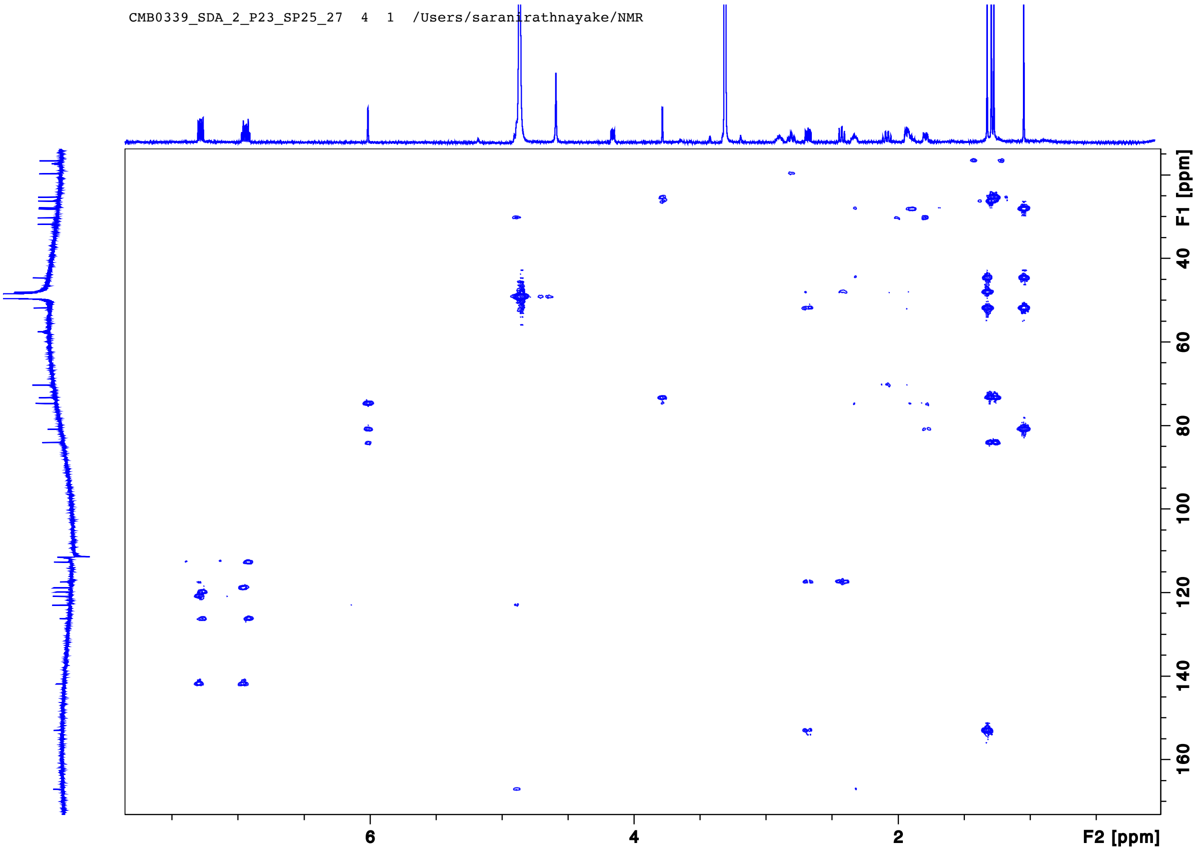

Figure S47. HMBC (methanol-d_4) spectrum of compound noonindole F (**10**)

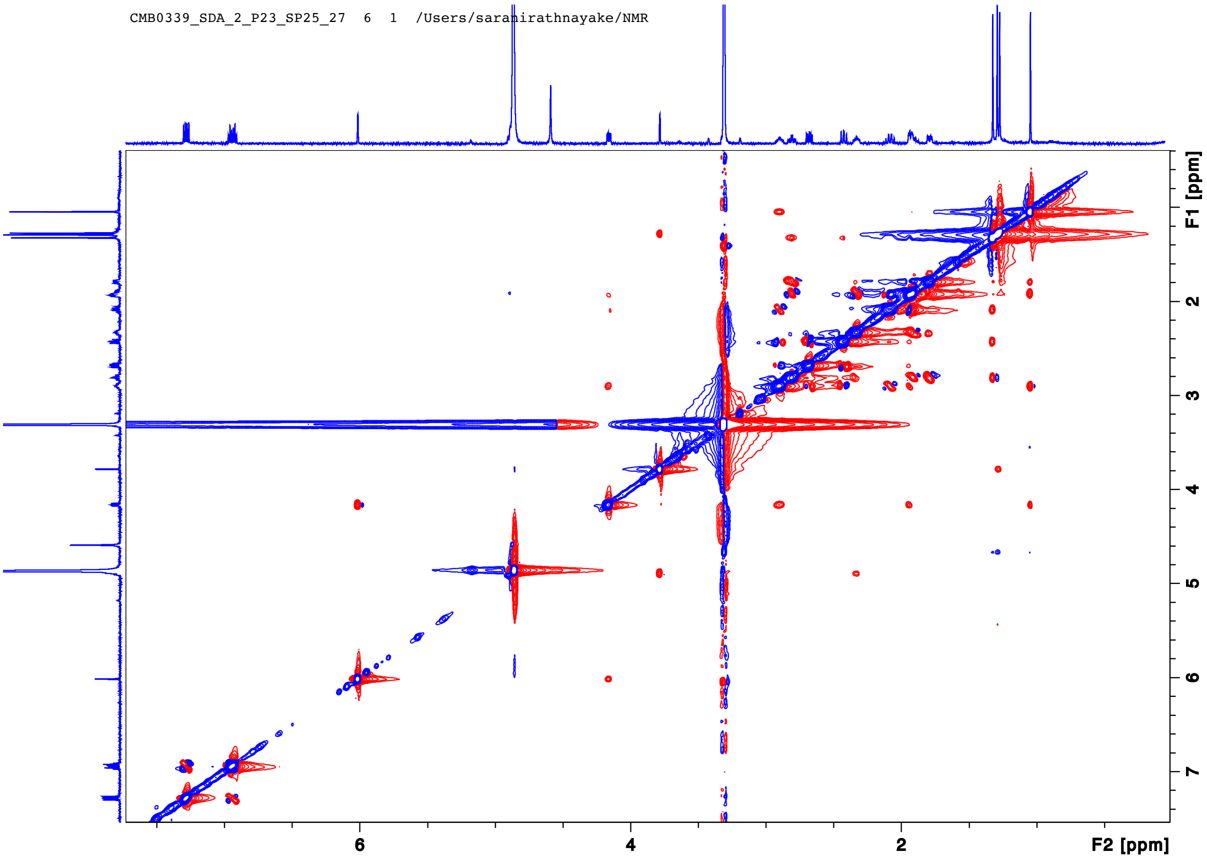

Figure S48. ROESY (methanol-d_4) spectrum of compound noonindole F (**10**)

Mass Spectrum Molecular Formula Report

Analysis Info

		Acquisition Date	11/30/2020 10:43:26 AM
Analysis Name	D:\Data\s.kankanamge\CMB0339_SDA_2_P23_SP25_27.d		
Method	tune-medhigh_AP.m	Operator	a.salim
Sample Name	CMB0339_SDA_2_P23_SP25_27	Instrument / Ser#	micrOTOF 213750.00232
Comment			

Acquisition Parameter

Source Type	ESI	Ion Polarity	Positive	Set Nebulizer	0.5 Bar
Focus	Not active			Set Dry Heater	180 °C
Scan Begin	100 m/z	Set Capillary	4500 V	Set Dry Gas	5.0 l/min
Scan End	1500 m/z	Set End Plate Offset	-500 V	Set Divert Valve	Source

Generate Molecular Formula Parameter

Formula, min.		
Formula, max.		
Measured m/z	Tolerance	Charge
Check Valence	Minimum	Maximum
Nirogen Rule	Electron Configuration	
Filter H/C Ratio	Minimum	Maximum
Estimate Carbon		

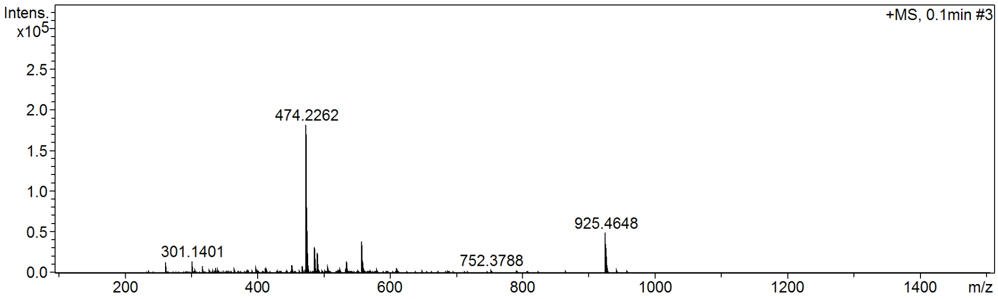

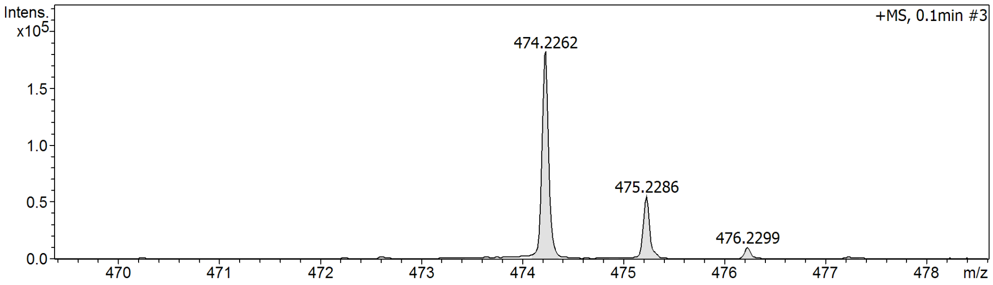

Meas. m/z	#	Ion Formula	m/z	err [ppm]	mSigma	# Sigma	Score	rdb	e⁻ Conf	N-Rule
474.2262	1	C27H33NNaO5	474.2251	-2.4	1.3	1	74.93	11.5	even	ok
	2	C28H29N5NaO	474.2264	0.4	12.0	2	100.00	16.5	even	ok
	3	C23H29N7NaO3	474.2224	-8.1	12.7	3	6.01	12.5	even	ok
	4	C17H29N11NaO4	474.2296	7.1	37.8	4	5.43	8.5	even	ok
	5	C16H33N7NaO8	474.2283	4.3	48.8	5	13.44	3.5	even	ok
	6	C13H25N17NaO2	474.2269	-1.5	57.5	6	18.24	9.5	even	ok
	7	C12H29N13NaO6	474.2256	-1.3	70.0	7	12.21	4.5	even	ok
	8	C9H21N23Na	474.2242	4.2	71.4	8	4.97	10.5	even	ok

Bruker Compass DataAnalysis 4.1 printed: 11/30/2020 10:49:20 AM Page 1 of 2

Figure S49. HRMS spectrum of noonindole F (**10**)

10 Paspaline (11)

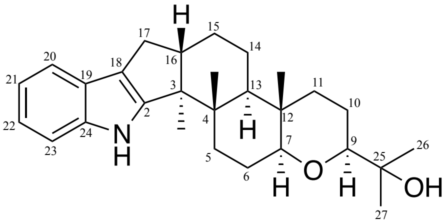

Table S8.1D and 2D NMR (DMSO-d_6, 600 MHz) data of paspaline (**11**)
(Reported data are shaded in grey)

Pos.	δ_H, mult. (*J* in Hz)	δ_C	COSY	^{1}H-^{13}C HMBC	δ_H, mult. (*J* in Hz) [1]	δ_C [1]
1-NH	10.56, s	-	-	2, 18, 19, 24	-	-
2	-	151.6	-	-	-	151.2
3	-	52.9	-	-	-	52.6
4	-	39.9	-	-	-	39.2
5	*a* 1.92 (m, 1H)	32.1	5*b*	4, 7, 13, 4-Me	1.91 (m, 1H)	31.1
	b 1.78 (m, 1H)		5*a*	4, 7, 4-Me	1.60 (m, 1H)	31.1
6	*a* 1.69[a] (m, 1H)	24.3	-	-	1.78 (m, 1H)	24.7
	b 1.55[b] (m, 1H)		-	-	1.72 (m, 1H)	24.7
7	2.97 (dd, 1H, J = *11.6, 3.4* Hz)	84.9	-	5, 9, 11, 12-Me	2.94 (dd, 1H, J = *11.5, 2.4* Hz)	85.4
9	3.08 (dd, 1H, J = *11.5, 1.8* Hz)	84.2	-	7, 25, 26, 27	3.09 (dd, 1H, J = *11.7, 2.2* Hz)	84.7
10	*a* 1.54[b] (m, 1H)	20.9	-	-	1.68 (m, 1H)	21.2
	b 1.47[c] (m, 1H)		-	-	1.44 (m, 1H)	21.2
11	*a* 1.75 (m, 1H)	37.0	11b	10, 12	1.30 (m, 1H)	37.4
	b 1.10 (m, 1H)		11*a*, 10b[c]	-	1.27 (m, 1H)	37.4
12	-	36.6	-		-	36.2
13	1.46[c] (m, 1H)	45.6	-	-	1.42 (m, 1H)	46.1
14	*a* 1.63 (br d, 1H, J = *12.6* Hz)	21.2	14*b*	4, 13, 15, 16	1.55 (m, 1H)	21.6
	b 1.33 (ddd, 1H, J = *16.5, 12.6, 4.1* Hz)		14*a*	4, 13, 15	1.28 (m, 1H)	21.6
15	*a* 1.70[a] (m, 1H)	24.8	-	-	1.77 (m, 1H)	25.3
	b 1.54[b] (m, 1H)		-	-	1.53 (m, 1H)	25.3
16	2.64 (m, 1H)	48.3	17*a*, 17*b*	3, 17, 3-Me	2.72 (t, 1H, J = *6.4* Hz)	48.7
17	*a* 2.55 (dd, 1H, J = *13.2*, *6.3* Hz)	26.8	17*b*, 16	2, 3, 16, 18	2.60 (m, 1H)	28.7
	b 2.23 (dd, 1H, J = *13.2*, *11.3* Hz)		17*a*, 16	2, 15, 16, 18	2.53 (m, 1H)	28.7
18	-	116.2	-	-	-	115.9
19	-	124.9	-	-	-	124.5
20	7.25[d] (dd, 1H, J = *7.4, 2.5* Hz)	117.2	-	-	7.25 (d, 1H, J = *7.9* Hz)	117.7
21	6.88 (dd, 1H, J = *7.9, 7.4* Hz)	118.0	20[d], 22	19, 23	6.92 (t, 1H, J = *6.7* Hz)	118.5
22	6.92 (dd, 1H, J = *7.9, 7.4* Hz)	118.9	21, 23[d]	20, 24	6.90 (t, 1H, J = *6.7* Hz)	119.4
23	7.25[d] (dd, 1H, J = *7.4, 2.5* Hz)	111.5	-	-	7.26 (d, 1H, J = *7.9* HZ)	112.0
24	-	140.8	-	-	-	140.4
25	-	70.9	-	-	-	70.5
26	1.04[e] (s, 3H)	24.4	-	-	1.03 (s, 3H)	26.8
27	1.09 (s, 3H)	26.7	-	9, 25, 29	1.05 (s, 3H)	28.9
12-Me	0.82 (s, 3H)	12.4	-	7, 11, 12, 13	0.81 (s, 3H)	14.0
3-Me	0.95 (s, 3H)	14.3	-	2, 3, 4, 16	1.10 (s, 3H)	14.6
4-Me	1.04[e] (s, 3H)	19.2	-	-	0.81 (s, 3H)	22.2
25-OH	4.07 (s, 1H)	-	-	9, 25, 26, 27	-	-

[a-e] Resonances with the same superscript within a column are overlapping and assignments may be interchanged
*Literature data were recorded (^{1}H NMR in 600 MHz and ^{13}C NMR in 150 MHz) in DMSO-d_6

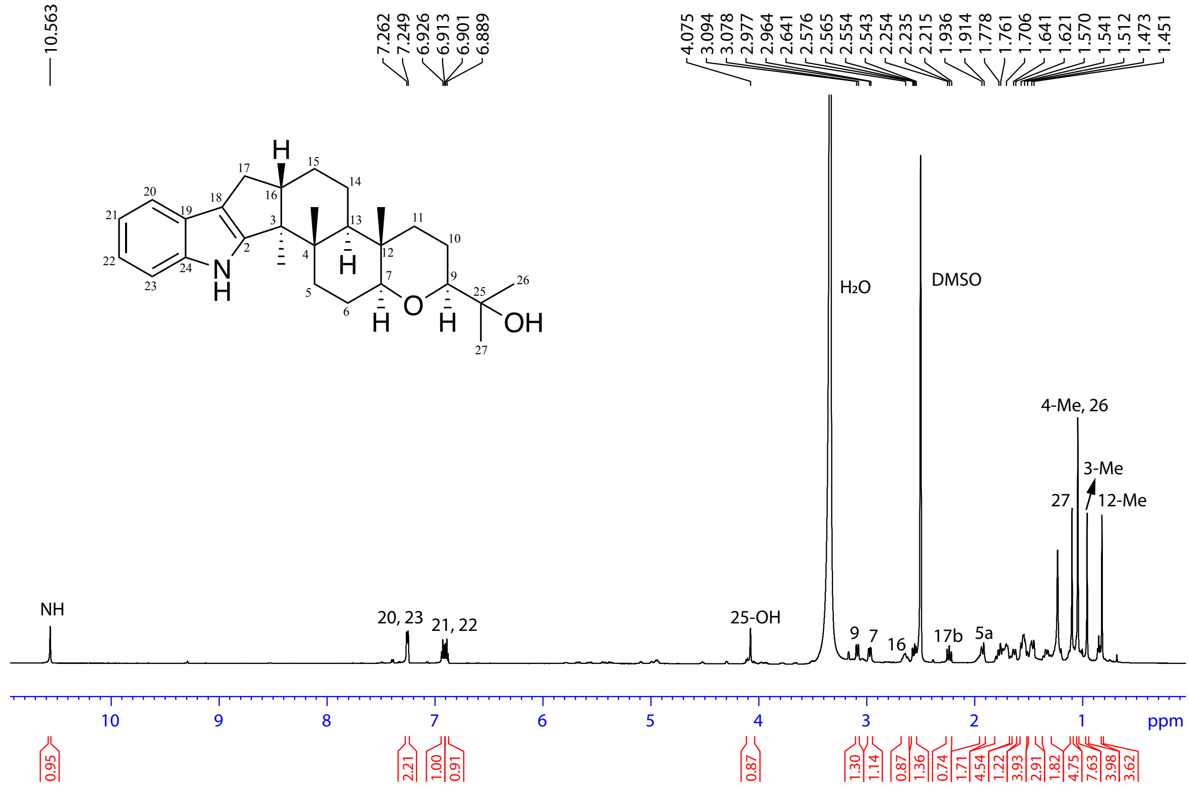

Figure S50. ¹H NMR (DMSO-d_6) spectrum of paspaline (**11**)

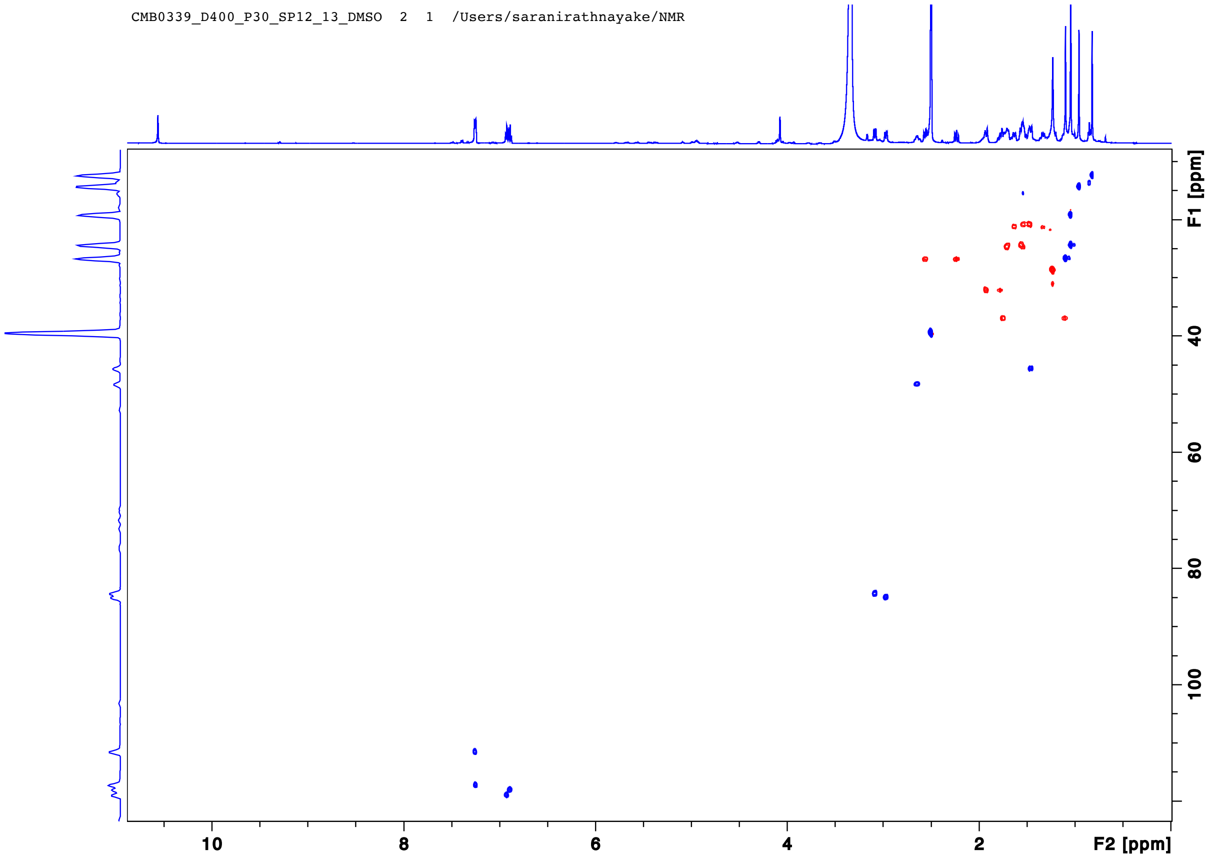

Figure S51. HSQC (DMSO-d_6) spectrum of paspaline (**11**)

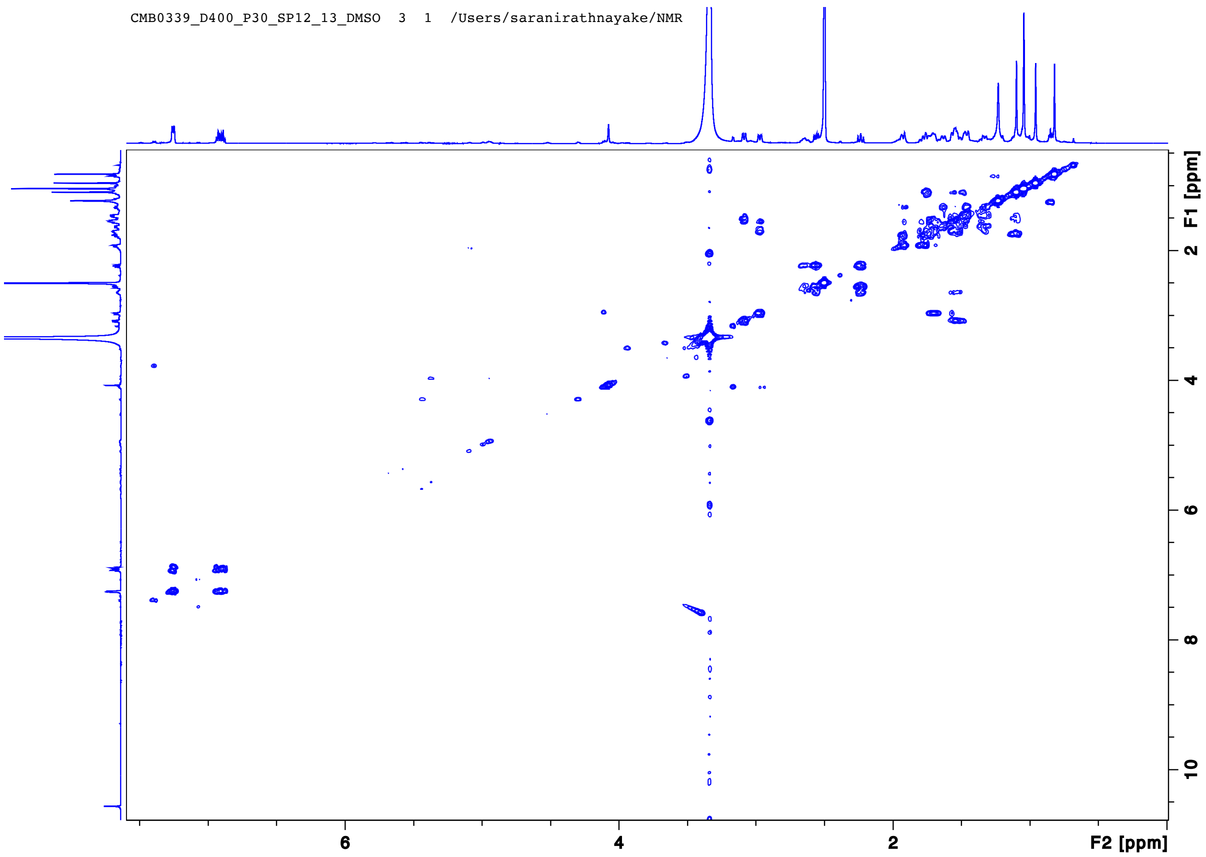

Figure S52. COSY (DMSO-d_6) spectrum of paspaline (**11**)

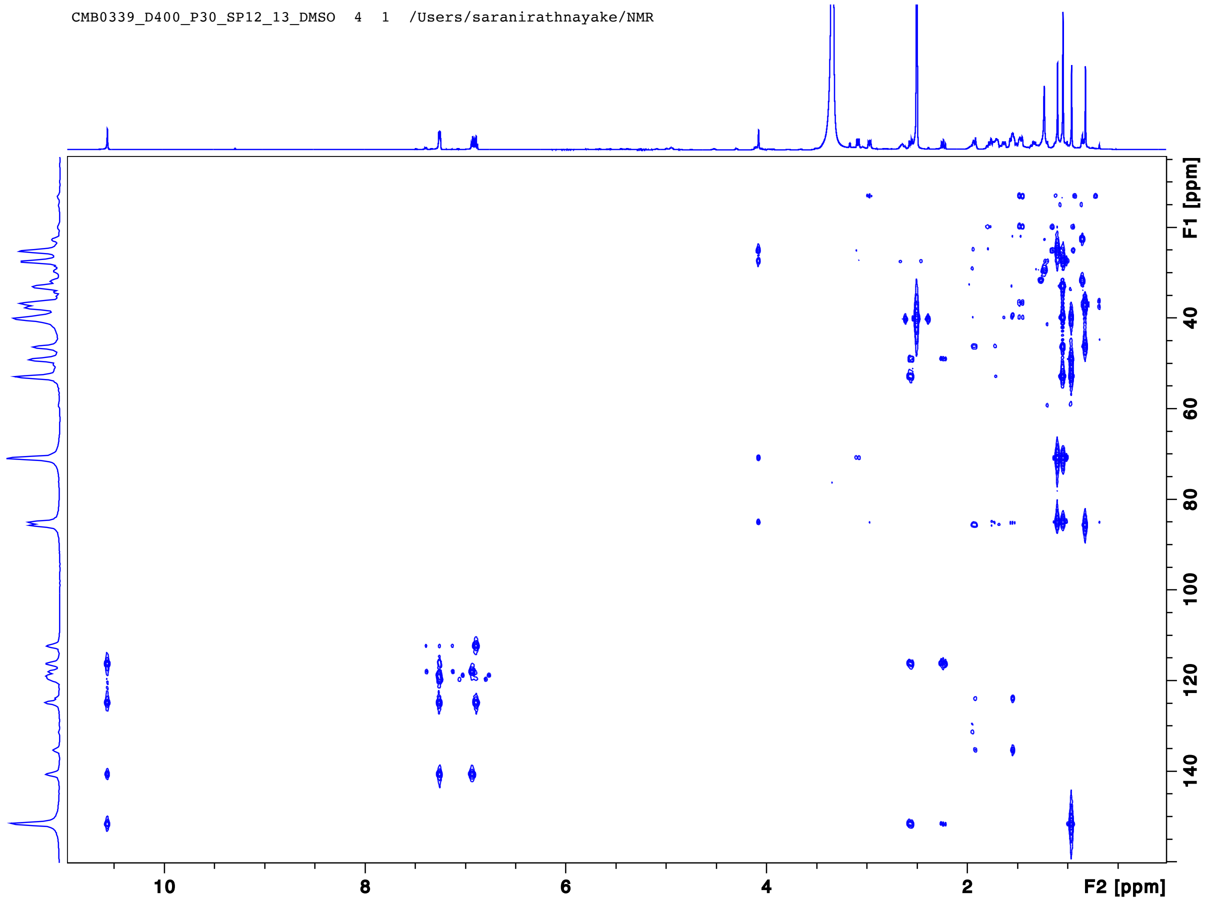

Figure S53. HMBC (DMSO-d_6) spectrum of paspaline (**11**)

Mass Spectrum Molecular Formula Report

Analysis Info

Analysis Name	D:\Data\s.kankanamge\CMB0339_D400_P30_SP12_13.d	Acquisition Date	9/8/2021 4:50:26 PM
Method	tune-medhigh_AP.m	Operator	a.salim
Sample Name	CMB0339_D400_P30_SP12_13	Instrument / Ser#	micrOTOF 213750.00232
Comment			

Acquisition Parameter

Source Type	ESI	Ion Polarity	Positive	Set Nebulizer	0.5 Bar
Focus	Not active			Set Dry Heater	180 °C
Scan Begin	100 m/z	Set Capillary	4500 V	Set Dry Gas	5.0 l/min
Scan End	1500 m/z	Set End Plate Offset	-500 V	Set Divert Valve	Source

Generate Molecular Formula Parameter

Formula, min.		
Formula, max.		
Measured m/z	Tolerance	Charge
Check Valence	Minimum	Maximum
Nirogen Rule	Electron Configuration	
Filter H/C Ratio	Minimum	Maximum
Estimate Carbon		

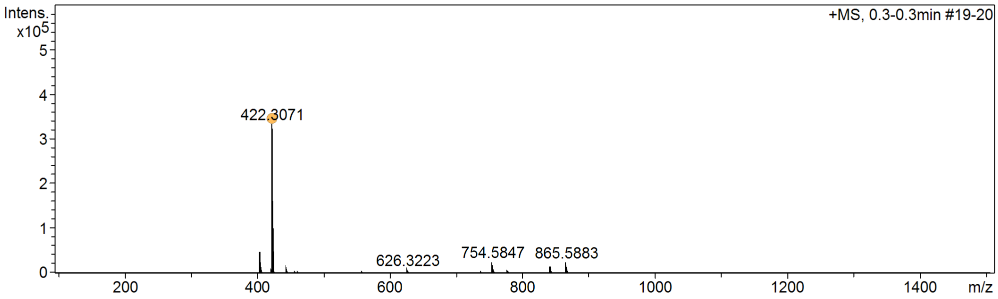

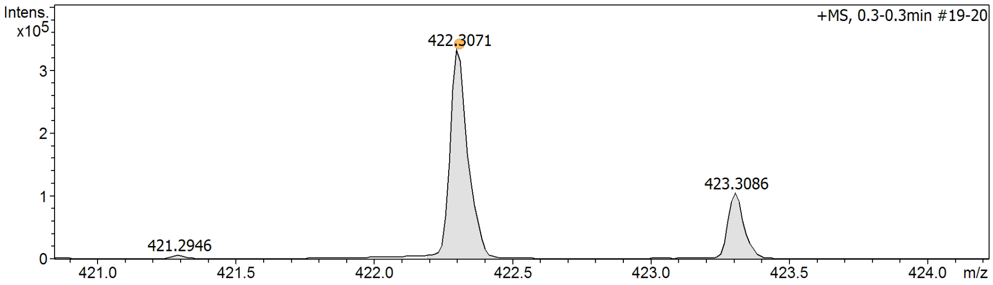

Meas. m/z	#	Ion Formula	m/z	err [ppm]	mSigma	# Sigma	Score	rdb	e⁻ Conf	N-Rule
422.3071	1	C28H40NO2	422.3054	4.1	3.0	1	100.00	9.5	even	ok

Bruker Compass DataAnalysis 4.1 printed: 10/4/2021 11:50:56 AM Page 1 of 1

Figure S54. HRMS spectrum of paspaline (**11**)

11 Paspaline B (12)

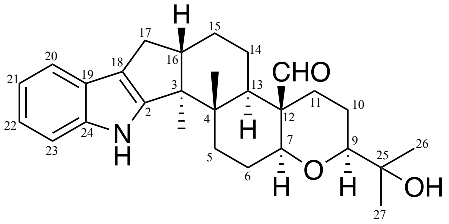

Table S9. 1D and 2D NMR ($CDCl_3$, 600 MHz) data of paspaline B (**12**)
(Reported data are shaded in grey)

Pos.	δ_H, mult. (J in Hz)	δ_C	COSY	^1H-^{13}C HMBC	δ_H, mult. (J in Hz) [2]	δ_C[2]
1-N<u>H</u>	7.67 (s, 1H)	-	-	18, 19, 24	7.69, s	-
2	-	149.9	-	-	-	150.0
3	-	51.9	-	-	-	52.0
4	-	39.9	-	-	-	39.9
5	*a* 2.02 (dd, 1H, J = *13.1, 3.9* Hz)	33.2	6*a*, 5*b*, 6*b*		2.07	33.2
	b 1.71 (dd, 1H, J = *10.2, 3.8* Hz)		5*a*	-	1.72	33.2
6	*a* 2.24 (dd, 1H, J = *12.3, 3.8* Hz)	24.8	7, 6*b*, 5*b*	-	2.04	24.9
	b 1.99 (m, 1H)		7, 6*a*	-	1.24	24.9
7	3.30 (dd, 1H, J = *12.3, 4.6* Hz)	83.6	6*a*, 6*b*	-	3.32	83.6
9	3.25 (dd, 1H, J = *11.7, 2.4* Hz)	84.8	10*b*	25	3.28	85.0
10	*a* 1.52*	23.1	-	-	1.55	23.4
	b 1.27 (m, 1H)		7	-	1.29	23.4
11	*a* 2.47 (m, 1H)	34.1	11*b*	-	2.49	33.9
	b 1.17 (m, 1H)		11*a*	9	1.22	33.9
12	-	50.6	-		-	50.7
13	1.68 (dd, 1H, J = *12.2, 3.5* Hz)	47.6	-	-	1.69	47.6
14	*a* 1.86 (m, 1H)	22.4	13	16	1.90	22.4
	b 1.52*		-	-	1.55	22.4
15	*a* 1.76 (m, 1H)	24.7	15*b*	-	1.80	24.9
	b 1.56*		-		1.55	24.9
16	2.68[a] (m, 1H)	48.7	-	-	2.70	48.7
17	*a* 2.66[a] (m, 1H)	27.3	-	2, 18, 16	2.68	27.4
	b 2.31 (m, 1H)		17*a*	18	2.32	27.4
18	-	118.4	-	-	-	118.0
19	-	125.7	-	-	-	125.7
20	7.40 (dd, 1H, J = *7.5, 1.6* Hz)	118.5	21[b]	22, 24	7.41	118.6
21	7.05[b] (m, 1H)	119.6	-	-	7.07	119.7
22	7.06[b] (m, 1H)	120.6	-	-	7.06	120.7
23	7.26 (dd, 1H, J = *7.8, 1.6* Hz)	111.4	22[b]	21, 19	7. 29	111.5
24	-	140.0	-		-	140.0
25	-	-	-		-	71.9
26	1.10 (s, 3H)	23.7	-	9, 25, 26	1.12	23.8
27	1.14 (s, 3H)	25.7		9, 25, 27	1.17	25.8
3-Me	0.98 (s, 3H)	14.8	-	2, 3, 4, 16	1.01	14.8
4-Me	0.91 (s, 3H)	19.6	-	3, 4, 5, 13	0.94	19.7
12-C<u>H</u>O	10.11 (s, 1H)	207.2	-	12	10.13	207.3

[a-b] Resonances with the same superscript within a column are overlapping and assignments may be interchanged
* Obscured by H_2O signal
**Literature data were recorded (^1H NMR in 300 MHz and ^{13}C NMR in 75 MHz) in $CDCl_3$

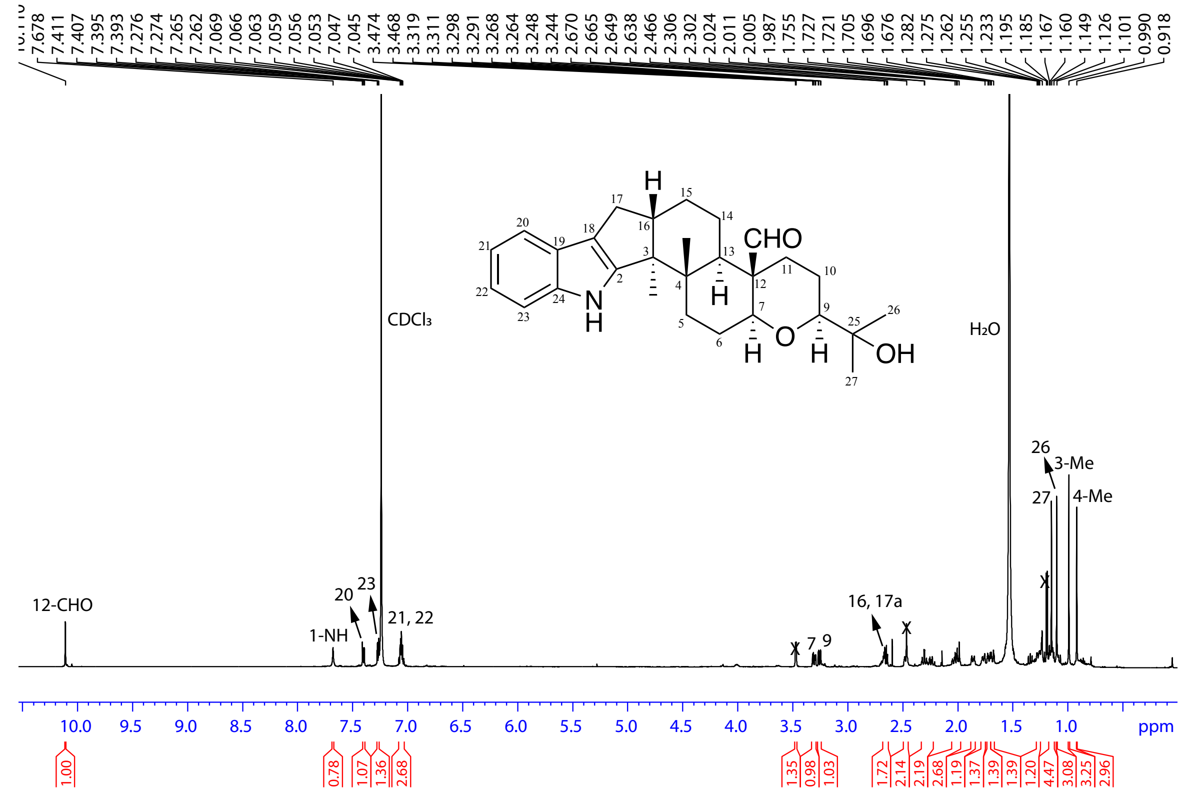

Figure S55. ^{1}H NMR ($CDCl_3$) spectrum of paspaline B (**12**)

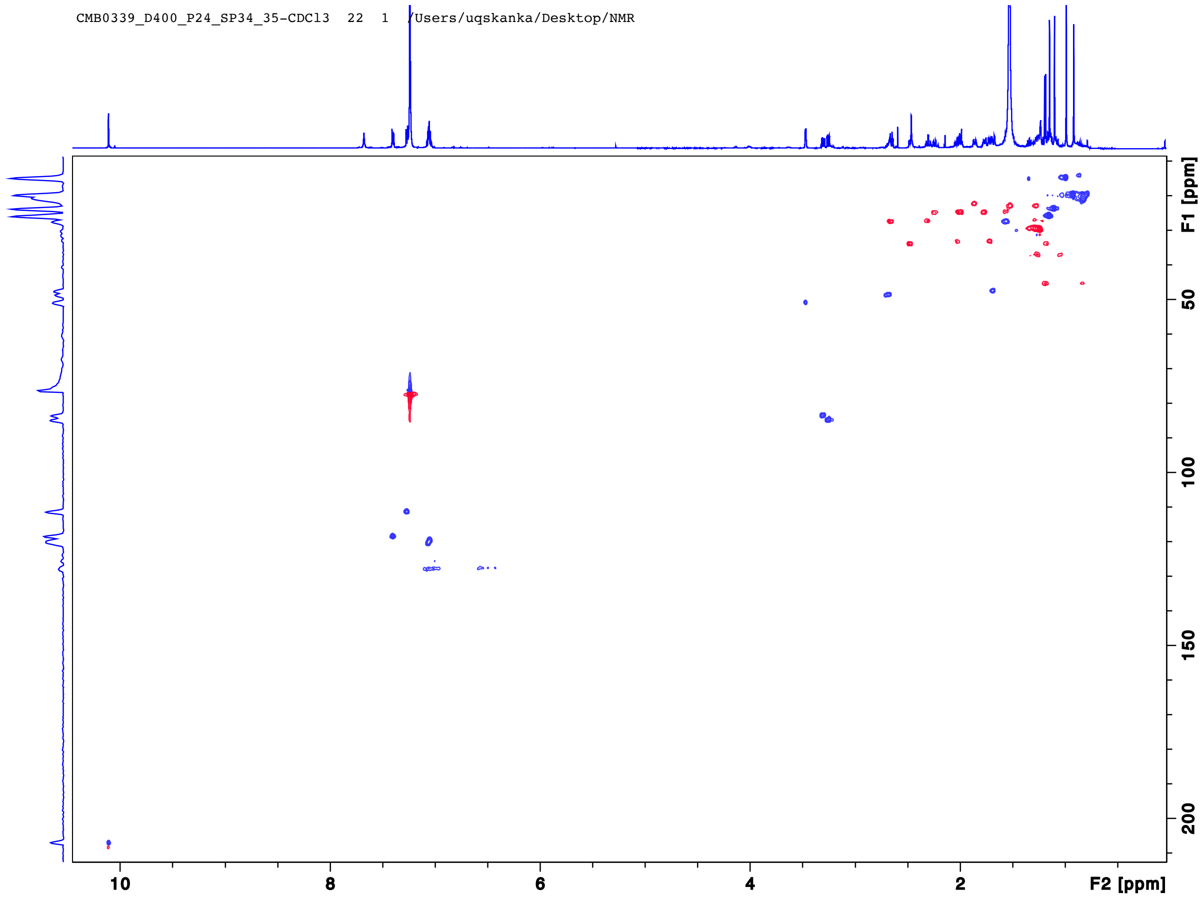

Figure S56. HSQC ($CDCl_3$) spectrum of paspaline B (**12**)

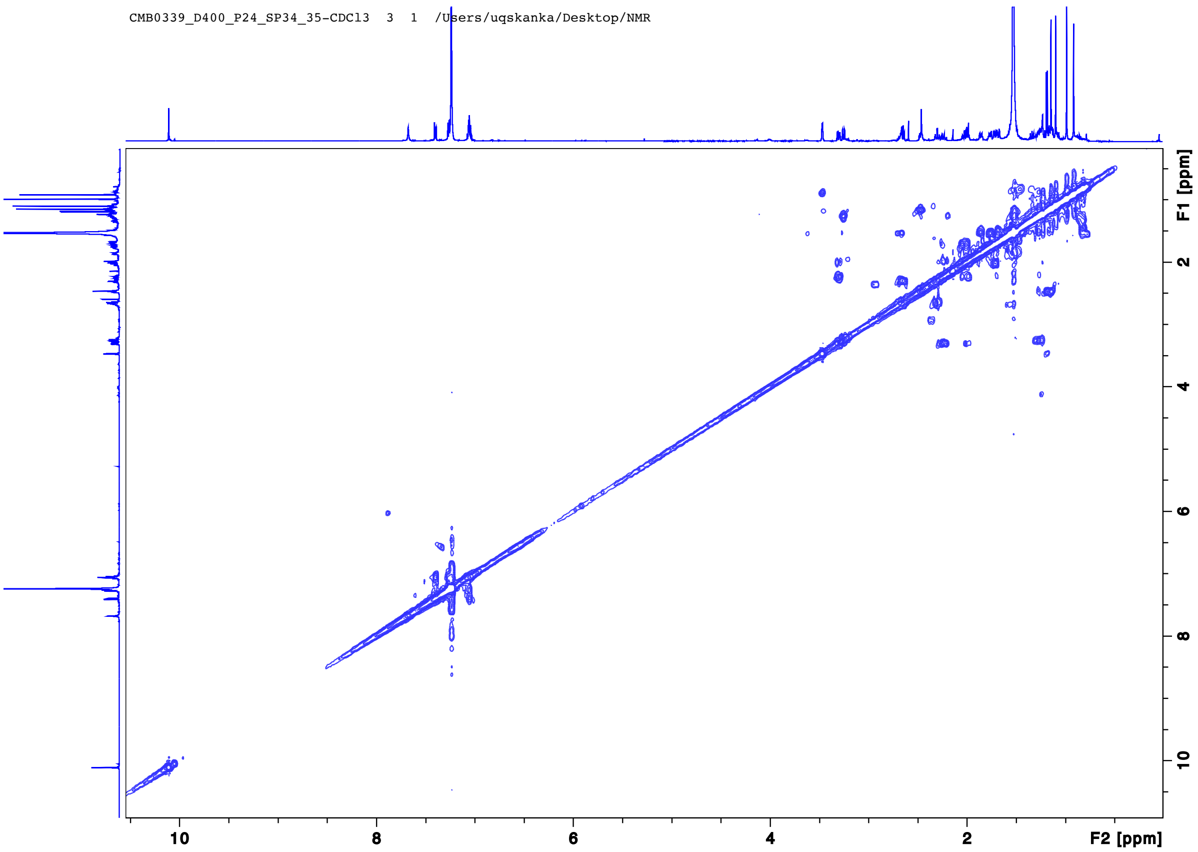

Figure S57. COSY ($CDCl_3$) spectrum of paspaline B (**12**)

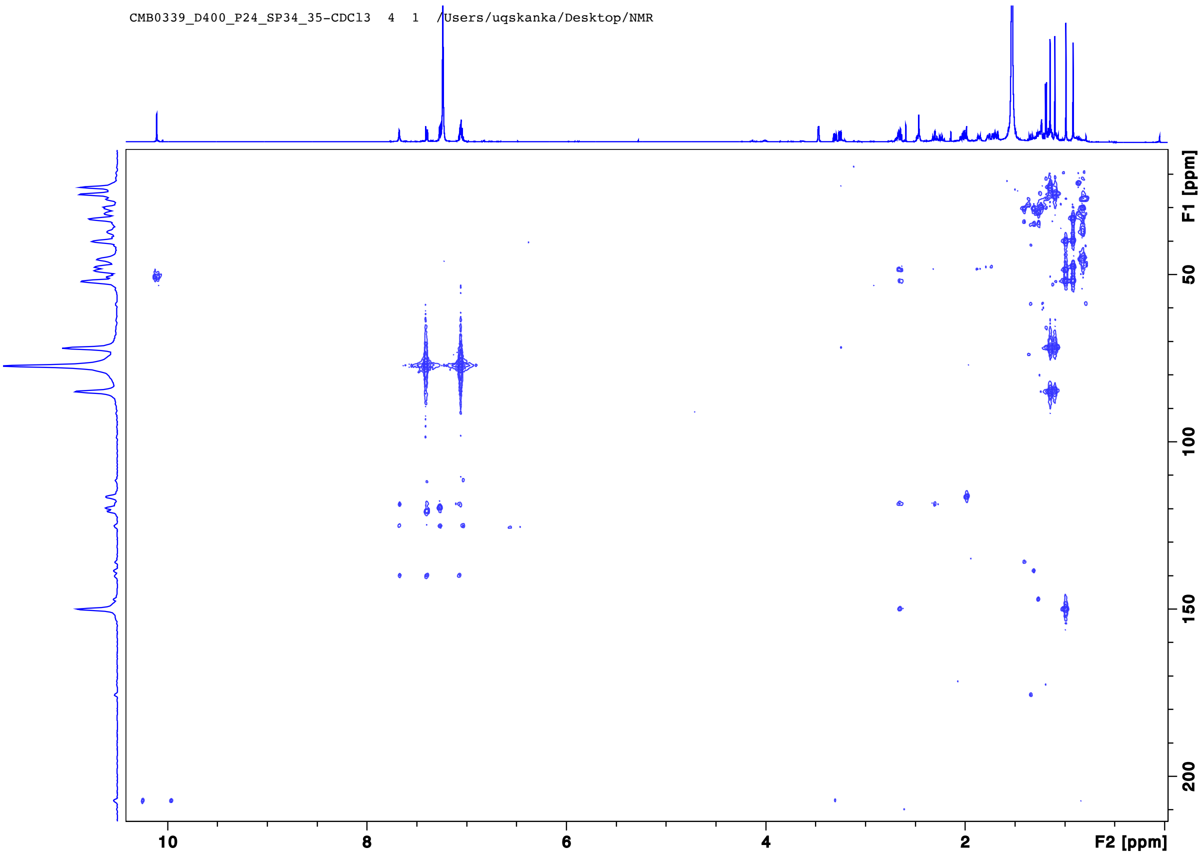

Figure S58. HMBC ($CDCl_3$) spectrum of paspaline B (**12**)

Mass Spectrum Molecular Formula Report

Analysis Info

Analysis Name	D:\Data\s.kankanamge\CMB0339_D400_P24_SP34_35.d	Acquisition Date	9/8/2021 11:13:58 AM
Method	tune-medhigh_AP.m	Operator	a.salim
Sample Name	CMB0339_D400_P24_SP34_35	Instrument / Ser#	micrOTOF 213750.00232
Comment			

Acquisition Parameter

Source Type	ESI	Ion Polarity	Positive	Set Nebulizer	0.5 Bar
Focus	Not active			Set Dry Heater	180 °C
Scan Begin	100 m/z	Set Capillary	4500 V	Set Dry Gas	5.0 l/min
Scan End	1500 m/z	Set End Plate Offset	-500 V	Set Divert Valve	Source

Generate Molecular Formula Parameter

Formula, min.		
Formula, max.		
Measured m/z	Tolerance	Charge
Check Valence	Minimum	Maximum
Nirogen Rule	Electron Configuration	
Filter H/C Ratio	Minimum	Maximum
Estimate Carbon		

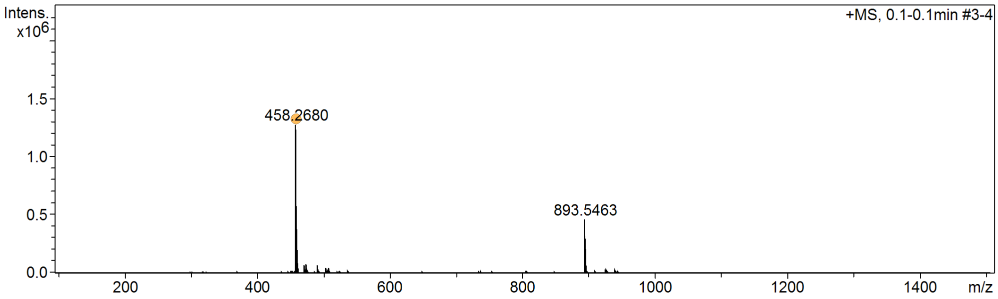

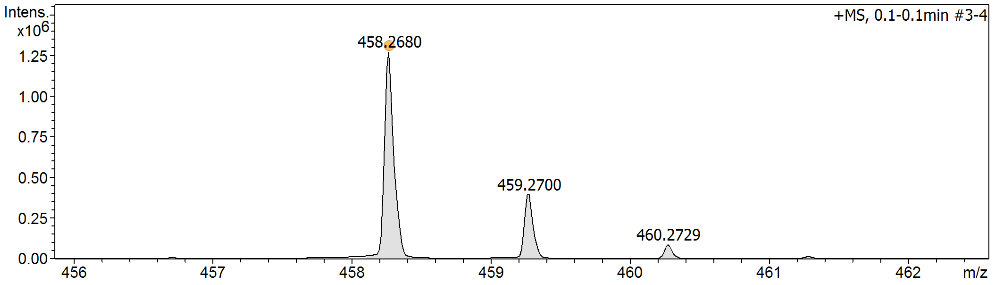

Meas. m/z	#	Ion Formula	m/z	err [ppm]	mSigma	# Sigma	Score	rdb	e⁻ Conf	N-Rule
458.2680	1	C28H37NNaO3	458.2666	-3.2	8.1	1	100.00	10.5	even	ok

Bruker Compass DataAnalysis 4.1 printed: 9/9/2021 8:06:18 AM Page 1 of 1

Figure S59. HRMS spectrum of paspaline B (**12**)

12 12-demethylpaspaline-12-carboxylic acid (13)

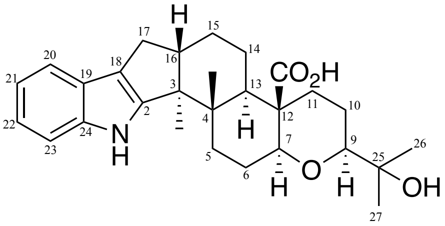

Table S10. 1D and 2D NMR (DMSO-d_6, 600 MHz) data of 12-demethylpaspaline-12-carboxylic acid (**13**) (Reported data are shaded in grey)

Pos.	$\delta_{H,}$ mult. (*J* in Hz)	δ_C	COSY	^{1}H-^{13}C HMBC	δ_H (mult., *J* (Hz) [1]	δ_C [1]
1-NH	10.58 (s, 1H)	-	-	2, 18, 19, 24	-	-
2	-	151.4	-	-	-	151.0
3	-	52.1	-	-	-	51.7
4	-	40.0	-	-	-	40.2
5	*a* 1.98 (br d, 1H, J = *12.7* Hz)	32.2	6*a*, 5b[a]	4, 6, 7, 13, 4-Me	*a* 1.97 (dt, 1H, J= *12.6, 3.1* Hz)	32.4
	b 1.82[a] (m, 1H)		-	-	*b* 1.83 (m, 1H)	
6	*a* 1.61[b] (m, 1H)	25.0	-	-	*a* 1.61 (m, 1H)	25.2
	b 2.55[c] (m, 1H)		-	-	*b* 2.54 (m, 1H)	
7	3.11[d] (br d, 1H, J = *11.3* Hz)	83.9[g]	10*b*	-	3.10 (dd, 1H, J = *11.0, 2.3* Hz)	83.7
9	3.11[d] (br d, 1H, J = *11.3 Hz*)	83.9[g]	10*b*	-	3.12 (dd, 1H, J = *11.1, 2.2* Hz)	84.3
10	*a* 1.62[b] (m, 1H)	22.5	-	-	*a* 1.65 (m, 1H)	22.8
	b 1.19 (m, 1H)		11*a*	9[d], 11	*b* 1.18 (m, 1H)	
11	*a* 2.36 (br d, 1H, J = *11.6* Hz)	35.0	11*b*, 10*a* [b]	10, 12, 7[d], 9[d], 12-CO_2H	*a* 2.35 (d, 1H, J = *11.4* Hz)	35.3
	b 1.24 (m, 1H)		11a	12, 10, 12-<u>C</u>O_2H	*b* 1.23 (m, 1H)	
12	-	47.8	-	-	-	47.3
13	1.67[e] (m, 1H)	45.8	-	-	1.67 (m, 1H)	46.1
14	*a* 1.68[e] (m, 1H)	23.1	-	-	*a* 1.61 (m, 1H)	21.6
	b 1.82[a] (m, 1H)		-	-	*b* 1.81 (m, 1H)	
15	*a* 1.68[e] (m, 1H)	24.5	-	-	*a* 1.69 (m, 1H)	24.8
	b 1.50 (m, 1H)		16	14, 16	*b* 1.50 (m, 1H)	
16	2.62 (m, 1H)	48.4	15*b*, 17*b*	3, 17, 3-Me	2.61 (m, 1H)	48.7
17	*a* 2.57[c] (m, 1H)	26.7	-	-	*a* 2.57 (dd, 1H, J= *12.9, 6.4* Hz)	27.0
	b 2.24 (dd, 1H, J = *12.6*, *11.0* Hz)		16, 17*a*[c]	2, 15, 16, 18	*b* 2.24 (dd, 1H, J= *12.9, 10.6* Hz)	
18	-	116.2	-	-	-	115.9
19	-	124.9	-	-	-	124.4
20	7.25[f] (dd, 1H, J = *7.4, 2.6* Hz)	117.3	-	-	7.24 (d, 1H, J = *7.9* Hz)	117.7
21	6.89 (t, 1H, J = *7.4* Hz)	118.1	20[f], 22	19, 23	6.89 (t, 1H, J = *7.9* Hz)	118.5
22	6.93 (t, 1H, J = *7.4* Hz)	119.0	21, 23[f]	20, 24	6.93 (t, 1H, J = *7.9* Hz)	119.4
23	7.25[f] (dd, 1H, J = *7.4, 2.6* Hz)	111.6	-	-	7. 26 (d, 1H, J = *7.9* Hz)	111.9
24	-	140.7	-	-	-	140.3
25	-	70.8	-	-	-	70.4
26	0.99 (s, 3H)	24.7	-	9[d], 25, 27	0.99 (s, 3H)	24.5
27	1.08 (s, 3H)	26.7	-	9[d], 25, 26	1.09 (s, 3H)	27.0
12-CO_2H	-	175.7	-	-	-	175.4
3-Me	0.93 (s, 3H)	14.1	-	2, 3, 4, 16	0.92 (s, 3H)	14.5
4-Me	0.97 (s, 3H)	15.7	-	3, 4, 5, 13	0.97 (s, 3H)	16.2
25-O<u>H</u>	4.04 (s, 1H)	-	-	9[d], 25, 26, 27	-	-

[a-g] Resonances with the same superscript within a column are overlapping and assignments may be interchanged.

*Literature data were recorded (^{1}H NMR in 600 MHz and ^{13}C NMR in 150 MHz) in DMSO-d_6

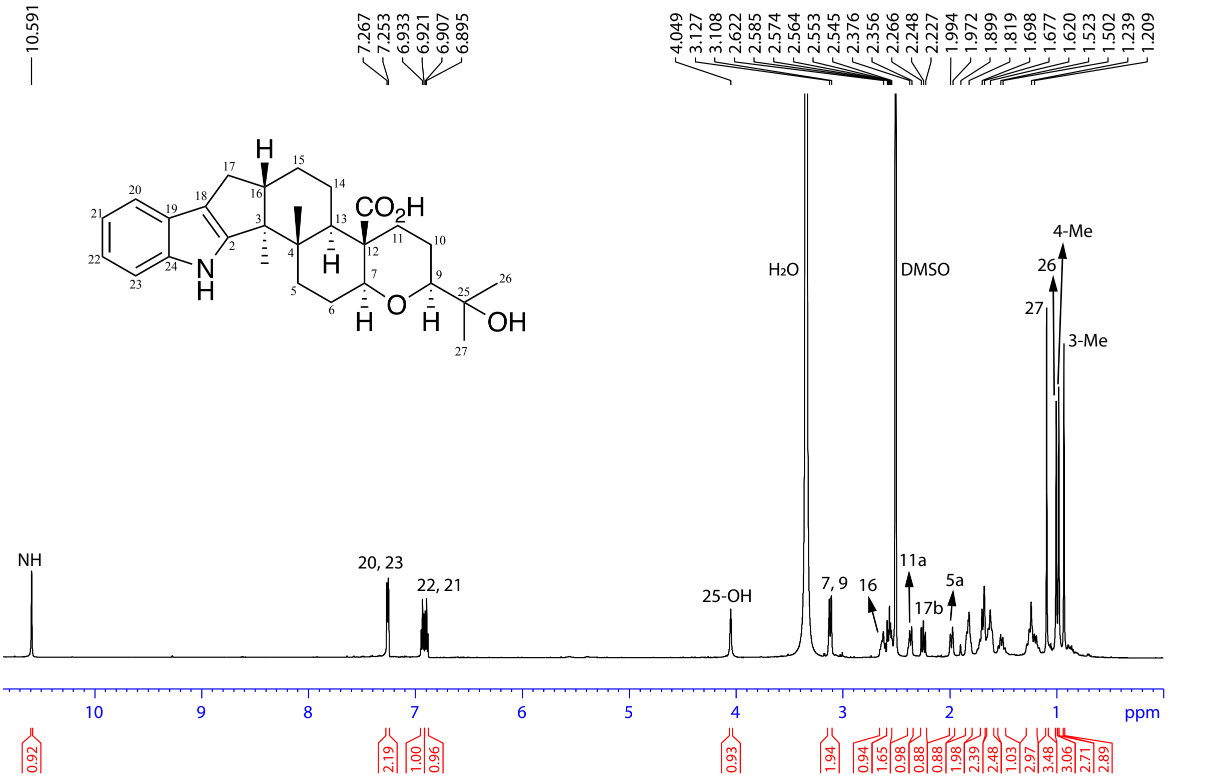

Figure S60. ^{1}H NMR (DMSO-d_6) spectrum of 12-demethylpaspaline-12-carboxylic acid (**13**)

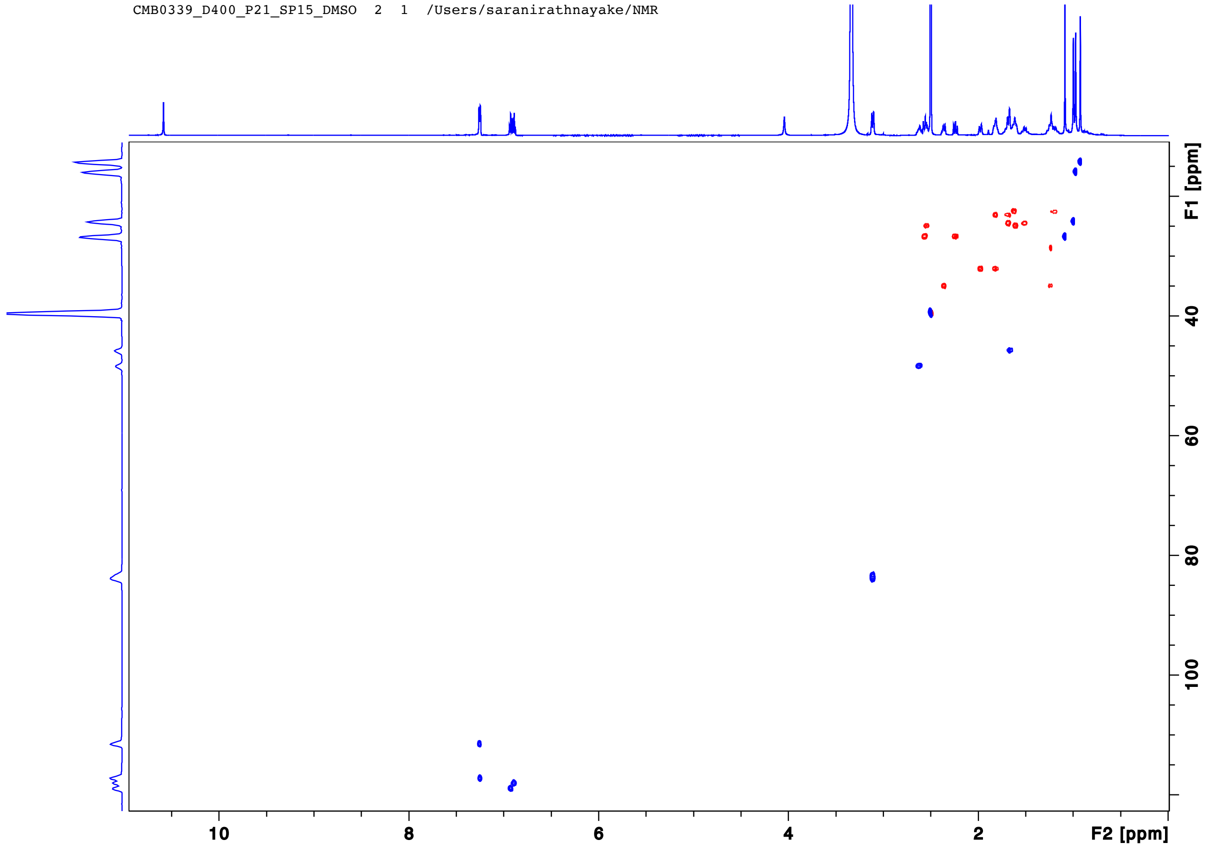

Figure S61. HSQC (DMSO-d_6) spectrum of 12-demethylpaspaline-12-carboxylic acid (**13**)

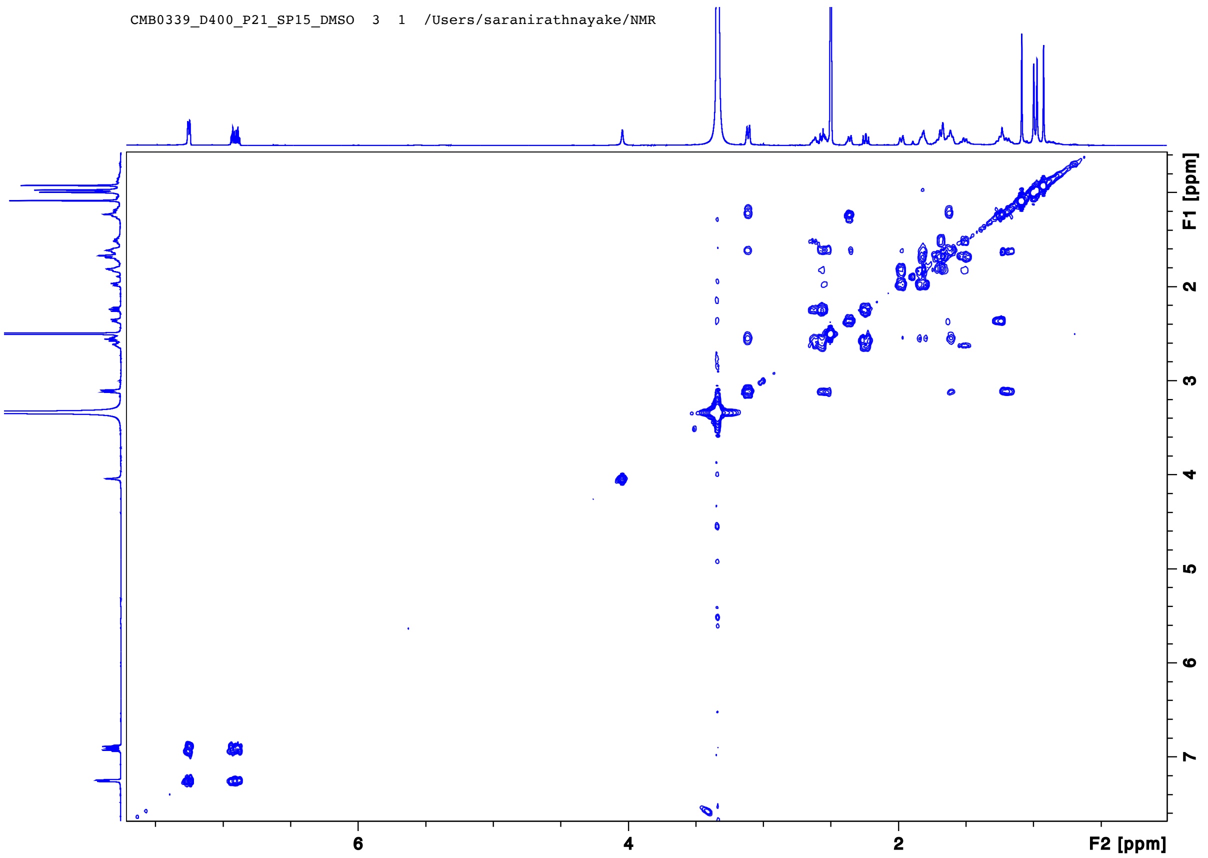

Figure S62. COSY (DMSO-d_6) spectrum of 12-demethylpaspaline-12-carboxylic acid (**13**)

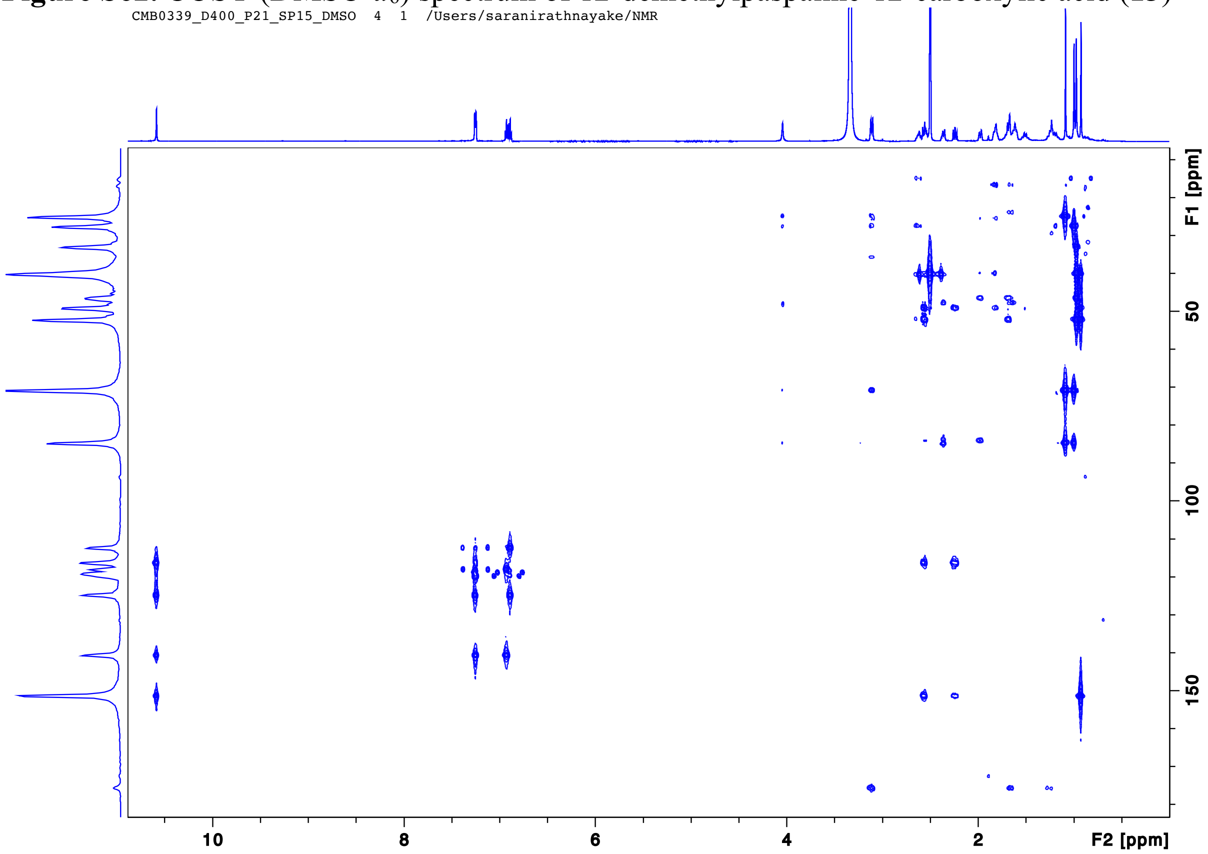

Figure S63. HMBC (DMSO-d_6) spectrum of 12-demethylpaspaline-12-carboxylic acid (**13**)

Mass Spectrum Molecular Formula Report

Analysis Info

Analysis Name	D:\Data\s.kankanamge\CMB0339_D400_P21_SP15.d	Acquisition Date	9/8/2021 4:57:44 PM
Method	tune-medhigh_AP.m	Operator	a.salim
Sample Name	CMB0339_D400_P21_SP15	Instrument / Ser#	micrOTOF 213750.00232
Comment			

Acquisition Parameter

Source Type	ESI	Ion Polarity	Positive	Set Nebulizer	0.5 Bar
Focus	Not active			Set Dry Heater	180 °C
Scan Begin	100 m/z	Set Capillary	4500 V	Set Dry Gas	5.0 l/min
Scan End	1500 m/z	Set End Plate Offset	-500 V	Set Divert Valve	Source

Generate Molecular Formula Parameter

Formula, min.		
Formula, max.		
Measured m/z	Tolerance	Charge
Check Valence	Minimum	Maximum
Nirogen Rule	Electron Configuration	
Filter H/C Ratio	Minimum	Maximum
Estimate Carbon		

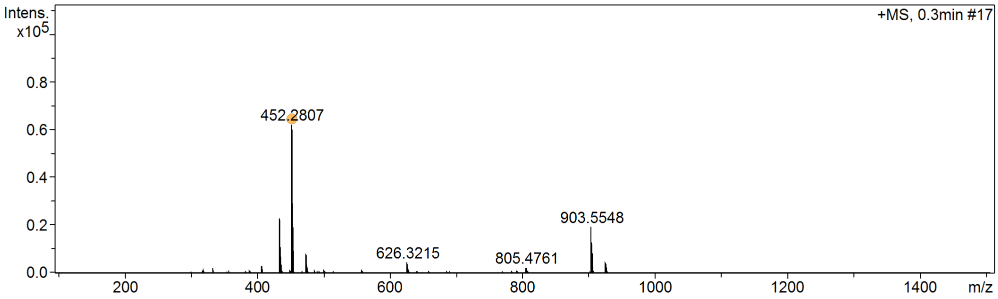

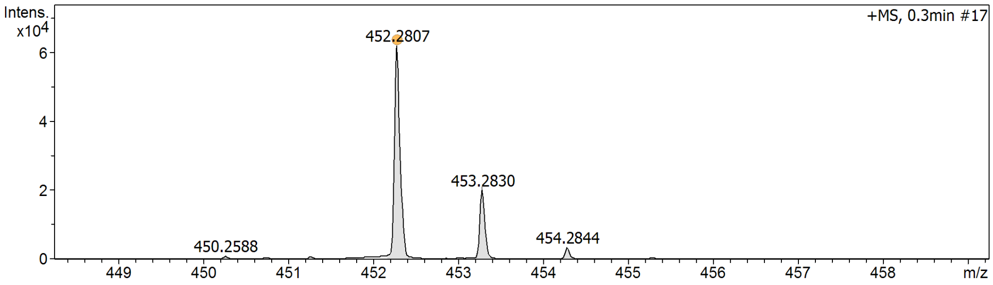

Meas. m/z	#	Ion Formula	m/z	err [ppm]	mSigma	# Sigma	Score	rdb	e⁻ Conf	N-Rule
452.2807	1	C28H38NO4	452.2795	2.7	4.7	1	61.14	10.5	even	ok
	2	C29H34N5	452.2809	-0.3	7.6	2	100.00	15.5	even	ok
	3	C26H36N4O3	452.2782	5.6	10.0	3	20.62	11.0	odd	ok
	4	C31H36N2O	452.2822	3.3	13.7	4	43.26	15.0	odd	ok
	5	C24H34N7O2	452.2768	8.6	16.1	5	4.69	11.5	even	ok
	6	C25H40O7	452.2769	8.6	21.7	6	4.19	6.0	odd	ok

Bruker Compass DataAnalysis 4.1 printed: 11/22/2021 12:06:01 PM Page 1 of 1

Figure S64. HRMS spectrum of 12-demethylpaspaline-12-carboxylic acid (**13**)

13 Emindole SB (14)

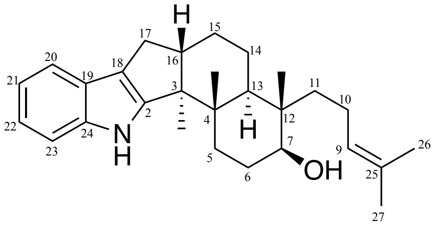

Table S11. 1D and 2D NMR (DMSO-d_6, 600 MHz) data of emindole SB (**14**) (Reported data are shaded in grey)

Pos.	δ_H, mult. (*J* in Hz)	δ_C	COSY	^{1}H-^{13}C HMBC	δ_H (mult., *J* (Hz) [1]	δ_C [1]
1-NH	10.55 (s, 1H)	-	-	2, 18, 19, 24	10.55 (s, 1H)	-
2	-	151.8		-	-	151.3
3	-	52.9		-	-	52.6
4	-	39.2		-	-	40.8
5	*a* 1.85 (m, 1H)	31.7	-	4, 7, 4-Me	*a* 1.84 (m, 1H)	32.0
	b 1.68[a] (m, 1H)		-	-	*b* 1.68 (m, 1H)	
6	*a* 1.68[a] (m, 1H)	27.0	-	-	*a* 1.67 (m, 1H)	27.4
	b 1.62 (m, 1H)		-	-	*b* 1.52 (m, 1H)	
7	3.38 (m, 1H)	71.0	7-OH	-	3.38 (m, 1H)	71.4
9	5.09 (t, 1H, J = *7.0* Hz)	124.9	10*a*, 10*b*	26, 27, 10	5.10 (t, 1H, J = *7.4* Hz)	125.2
10	*a* 1.92 (m, 1H)	20.7	10*b*, 9, 11*b*	9, 11, 25	*a* 1.92 (m, 1H)	21.1
	b 1.8 (m, 1H)		10*a*, 9	-	*b* 1.85 (m, 1H)	
11	*a* 1.56[b] (m, 1H)	36.9	-	-	*a* 1.54 (m, 1H)	37.2
	b 1.10 (ddd, 1H, J = *14.0, 12.6, 4.9* Hz)		10*a*, 10*b*	-	*b* 1.11 (m, 1H)	
12	-	41.3			-	38.7
13	1.66[a] (m, 1H)	39.1	-	-	1.67 (m, 1H)	39.6
14	*a* 1.52 (m, 1H)	22.1	14*b*, 15*a*	13, 16	*a* 1.51 (m, 1H)	22.4
	b 1.36 (ddd, 1H, J = *15.1, 13.5, 4.7* Hz)		14*a*	-	*b* 1.36 (m, 1H)	
15	*a* 1.70 (m, 1H)	24.7	14*a*, 16	-	*a* 1.69 (m, 1H)	25.0
	b 1.54[b] (m, 1H)		-	-	*b* 1.53 (m, 1H)	
16	2.64 (m, 1H)	48.2	17*a*, 17*b*	-	2.62 (m, 1H)	48.7
17	*a* 2.56 (dd, 1H, J = *13.0, 6.4* Hz)	26.7	17*b*, 16	2, 3, 16, 18	2.56 (dd, 1H, J = (*13.0, 6.4* Hz)	27.1
	b 2.23 (dd, 1H, J = *13.0, 10.8* Hz)		17*a*, 16	2, 16, 18	2.23 (m, 1H)	27.1
18	-	116.4	-	-	-	115.9
19	-	124.9		-	-	124.5
20	7.25 (d, 1H, J = *7.5* Hz)	117.2	21	22, 24	7.25 (d, 1H, J = *7.8* Hz)	117.6
21	6.88 (dt, 1H, J = *7.5, 1.3* Hz)	118.0	20, 22	19, 23	6.89 (td, 1H, J = *7.0, 1.2* Hz)	118.4
22	6.92 (dt, 1H, J = *7.5, 0.8* Hz)	118.9	21, 23	20, 24	6.93 (td, 1H, J = *7.0, 1.3* Hz)	119.3
23	7.26 (d, 1H, J = *7.5* Hz)	111.4	22	19, 21	7. 26 (d, 1H, J = *7.7* Hz)	111.9
24	-	140.7	-		-	140.3
25	-	130.4	-		-	129.9
26	1.58 (s, 3H)	17.2	-	9, 25, 26	1.59 (s, 3H)	17.5
27	1.64 (s, 3H)	25.2		9, 25, 27	1.65 (s, 3H)	25.6
12-Me	0.71 (s, 3H)	16.6	-	7, 11, 12, 13	0.71 (s, 3H)	16.9
3-Me	0.94 (s, 3H)	14.4	-	2, 3, 4, 16	0.93 (s. 3H)	14.6
4-Me	1.01 (s, 3H)	18.4	-	3, 4, 5, 13	1.01 (s, 3H)	18.7
7-OH	4.32 (d, 1H, J = *5.0* Hz)	-	7	-	4.36 (d, 1H, J = *5.2* Hz)	-

[a-b] Resonances with the same superscript are overlapping and assignments may be interchanged
*Literature data were recorded (^{1}H NMR in 600 MHz and ^{13}C NMR in 150 MHz) in DMSO-d_6

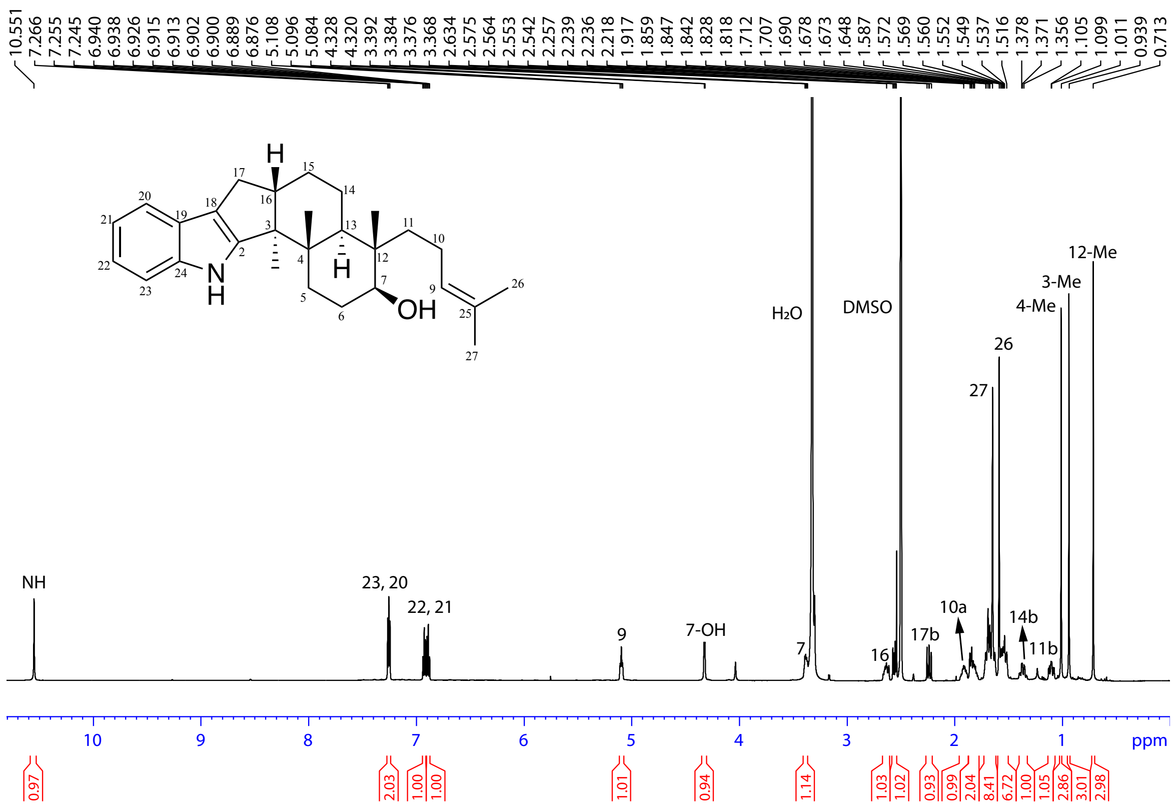

Figure S65. ^{1}H NMR (DMSO-d_6) spectrum of emindole SB (**14**)

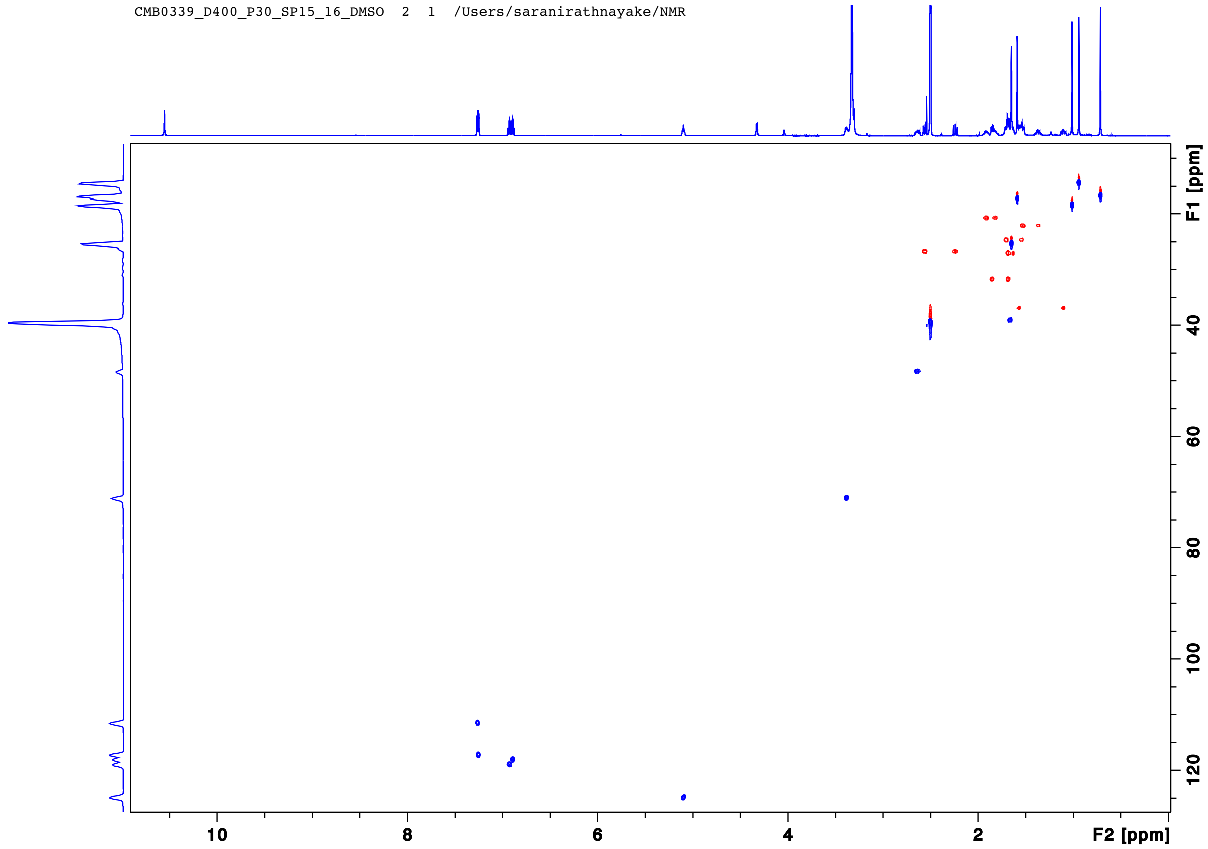

Figure S66. HSQC (DMSO-d_6) spectrum of emindole SB (**14**)

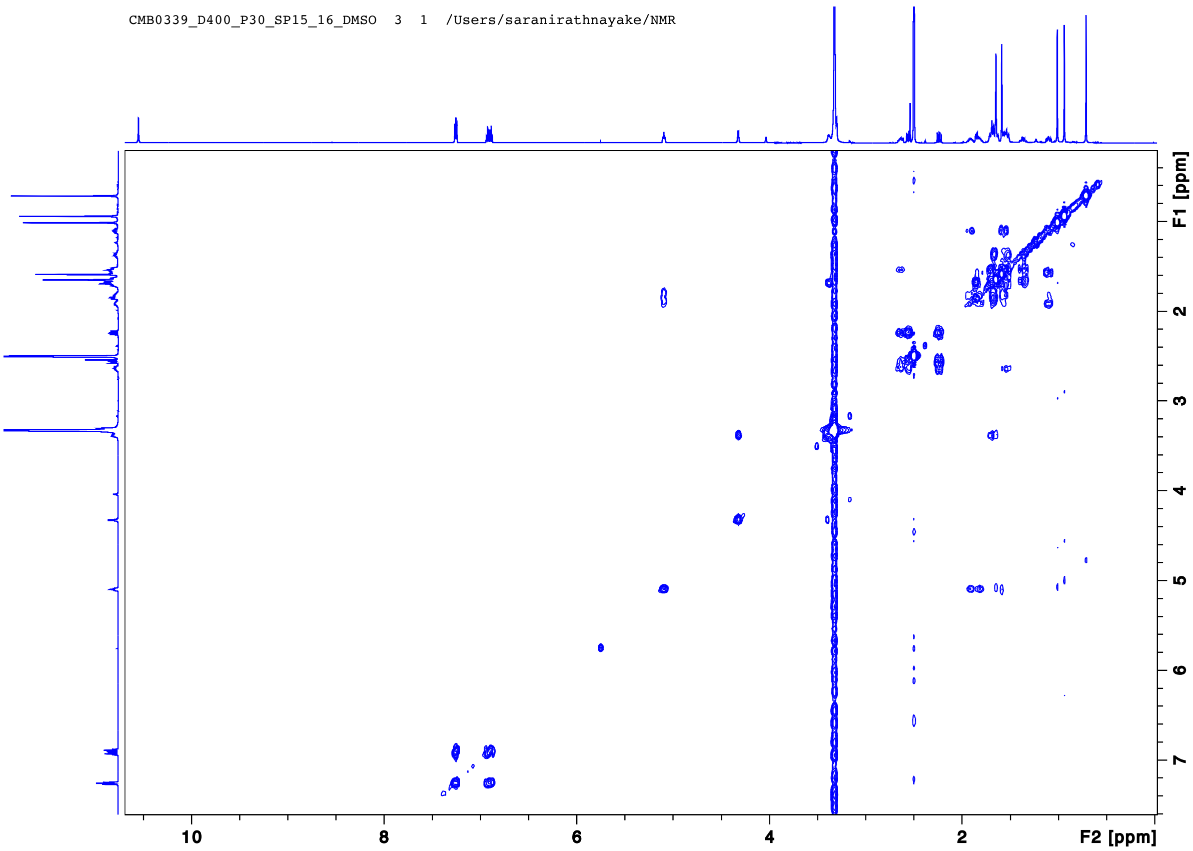

Figure S67. COSY (DMSO-d_6) spectrum of emindole SB (**14**)

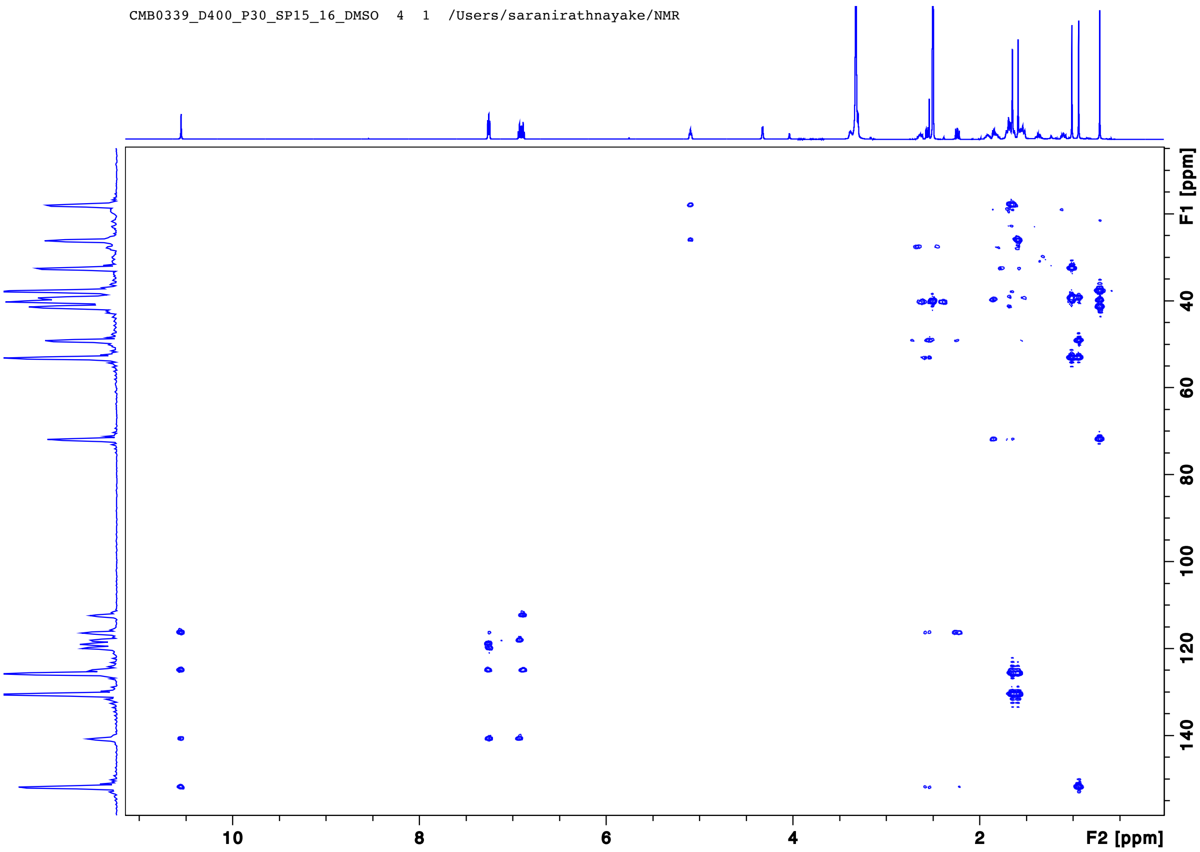

Figure S68. HMBC (DMSO-d_6) spectrum of emindole SB (**14**)

Mass Spectrum Molecular Formula Report

Analysis Info

Analysis Name	D:\Data\s.kankanamge\CMB0339_D400_P30_SP15_16.d	Acquisition Date	9/8/2021 4:54:59 PM
Method	tune-medhigh_AP.m	Operator	a.salim
Sample Name	CMB0339_D400_P30_SP15_16	Instrument / Ser#	micrOTOF 213750.00232
Comment			

Acquisition Parameter

Source Type	ESI	Ion Polarity	Positive	Set Nebulizer	0.5 Bar
Focus	Not active			Set Dry Heater	180 °C
Scan Begin	100 m/z	Set Capillary	4500 V	Set Dry Gas	5.0 l/min
Scan End	1500 m/z	Set End Plate Offset	-500 V	Set Divert Valve	Source

Generate Molecular Formula Parameter

Formula, min.		
Formula, max.		
Measured m/z	Tolerance	Charge
Check Valence	Minimum	Maximum
Nirogen Rule	Electron Configuration	
Filter H/C Ratio	Minimum	Maximum
Estimate Carbon		

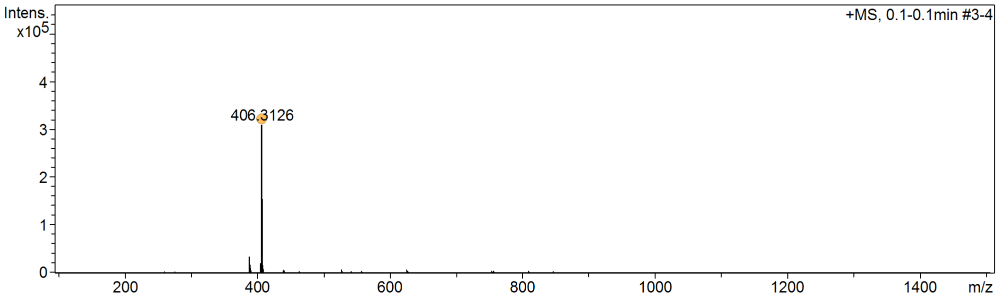

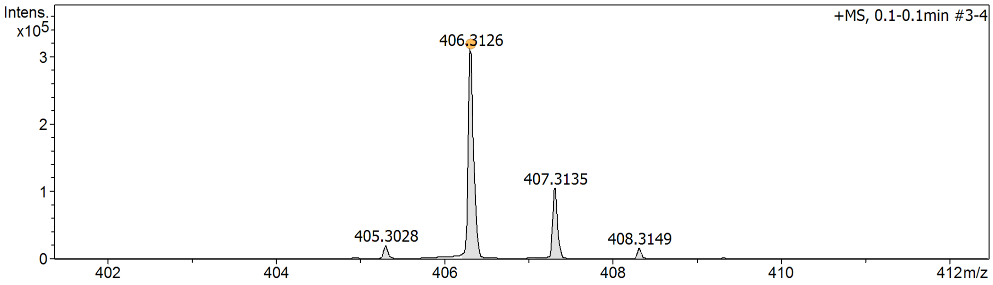

Meas. m/z	#	Ion Formula	m/z	err [ppm]	mSigma	# Sigma	Score	rdb	e¯ Conf	N-Rule
406.3126	1	C28H40NO	406.3104	5.3	12.5	1	100.00	9.5	even	ok

Bruker Compass DataAnalysis 4.1 printed: 10/7/2021 2:27:04 PM Page 1 of 1

Figure S69. HRMS spectrum of emindole SB (**14**)

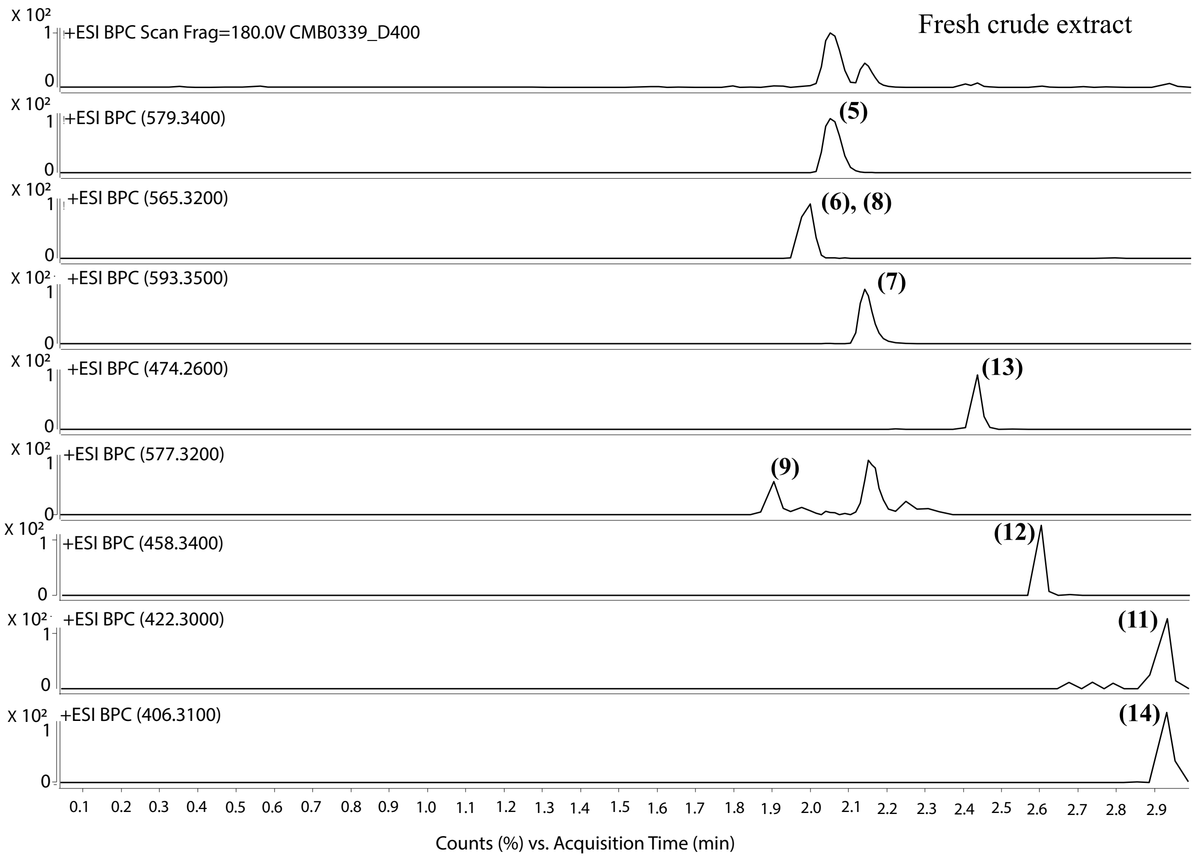

Figure S70. Single ion extraction of CMB-M0339 fresh crude extract to show the presence of isolated metabolites

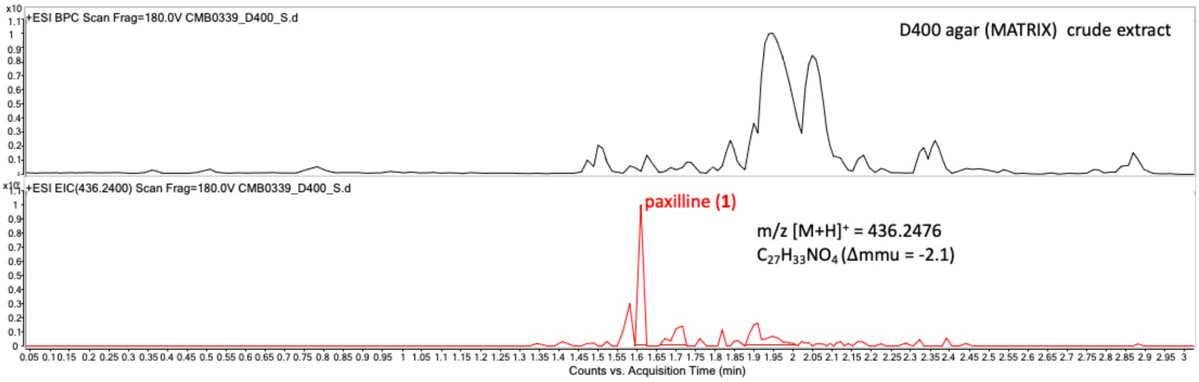

Figure S71. Single ion extraction of crude extract of CMB-M0339 grown on D400 agar (MATRIX) to show the presence of paxilline (**1**)

14 MS/MS fragmentation patterns of 5 - 9 and i-v

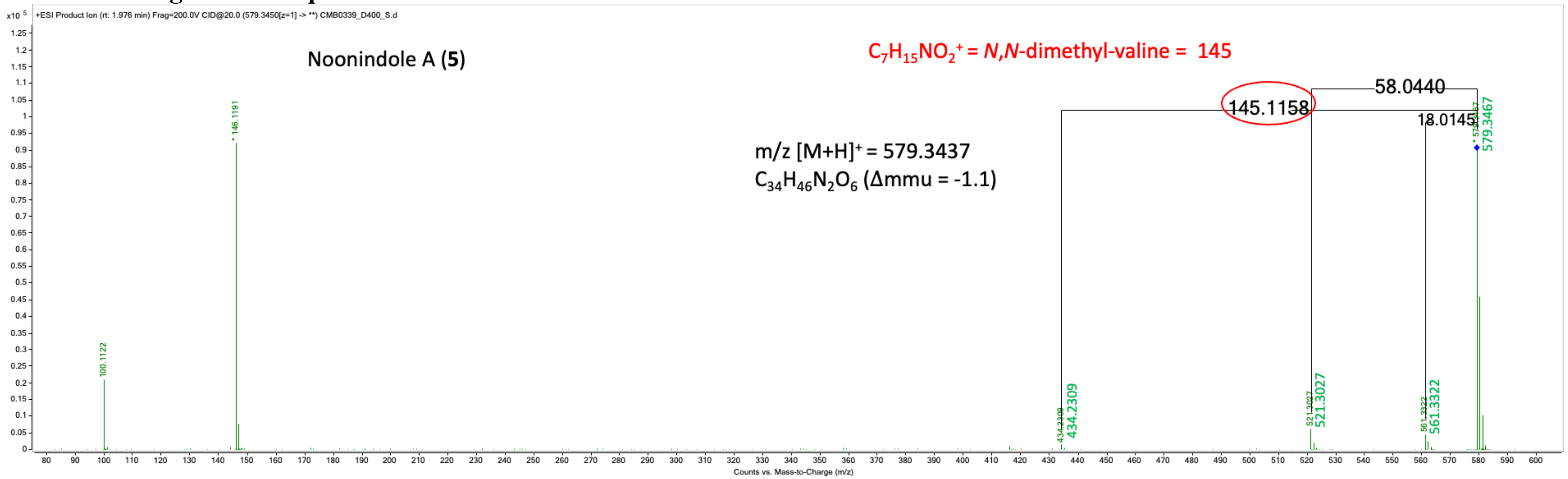

Figure S72. MS/MS fragmentation pattern of (**5**)

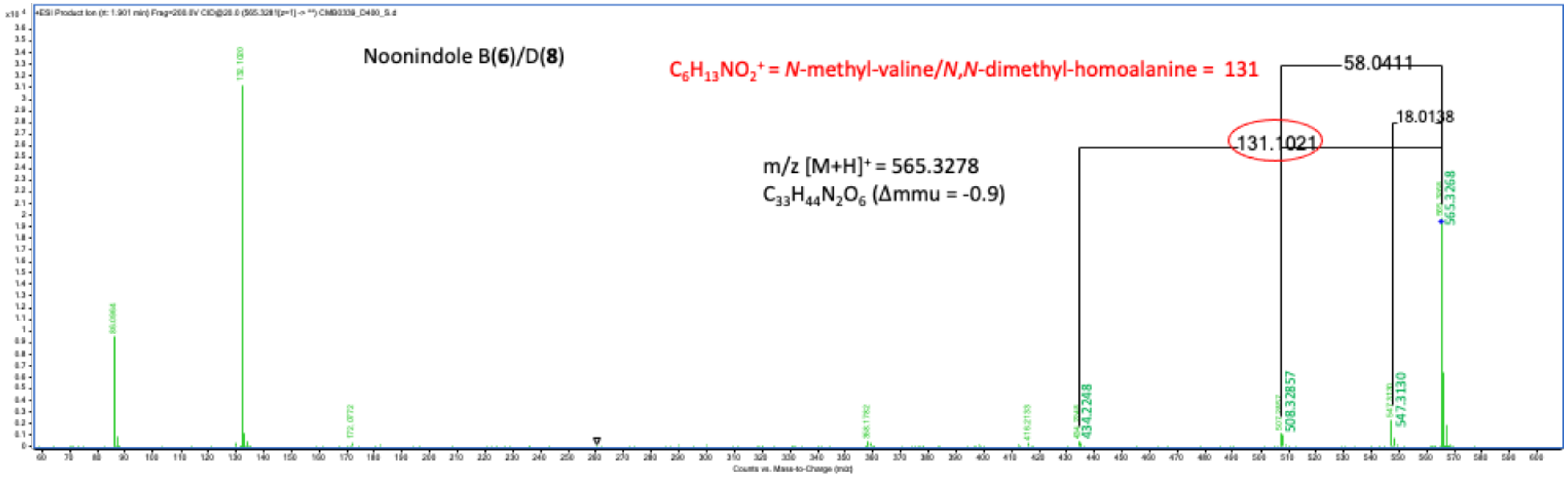

Figure S73. MS/MS fragmentation pattern of (**6**)/(**8**)

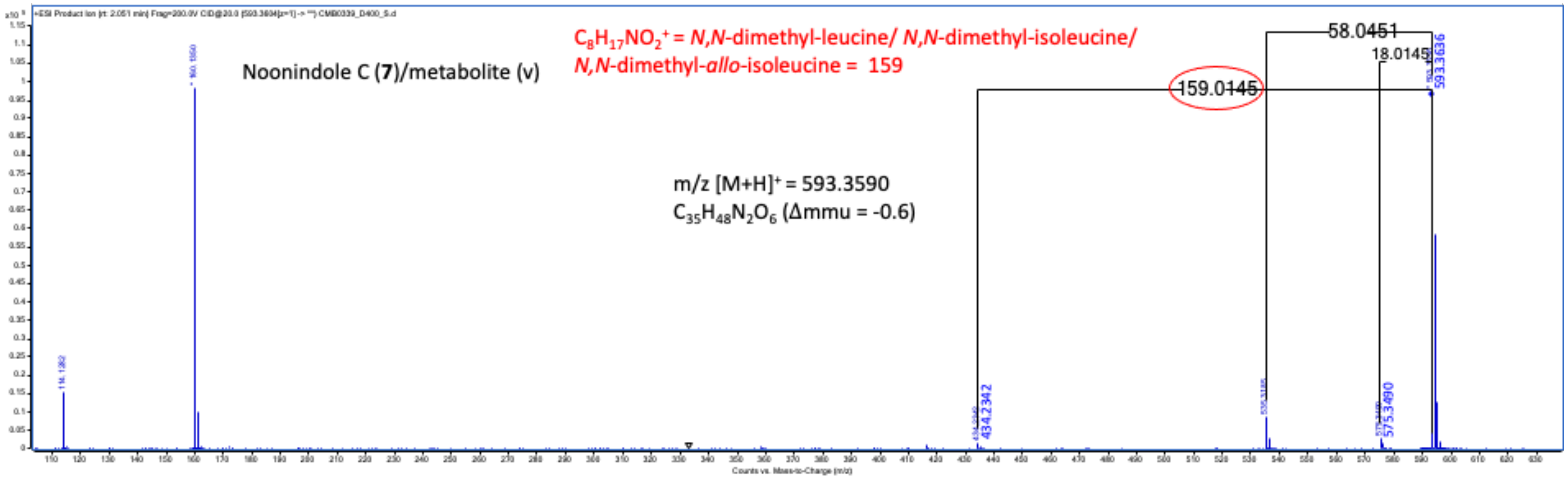

Figure S74. MS/MS fragmentation pattern of (**7**)/v

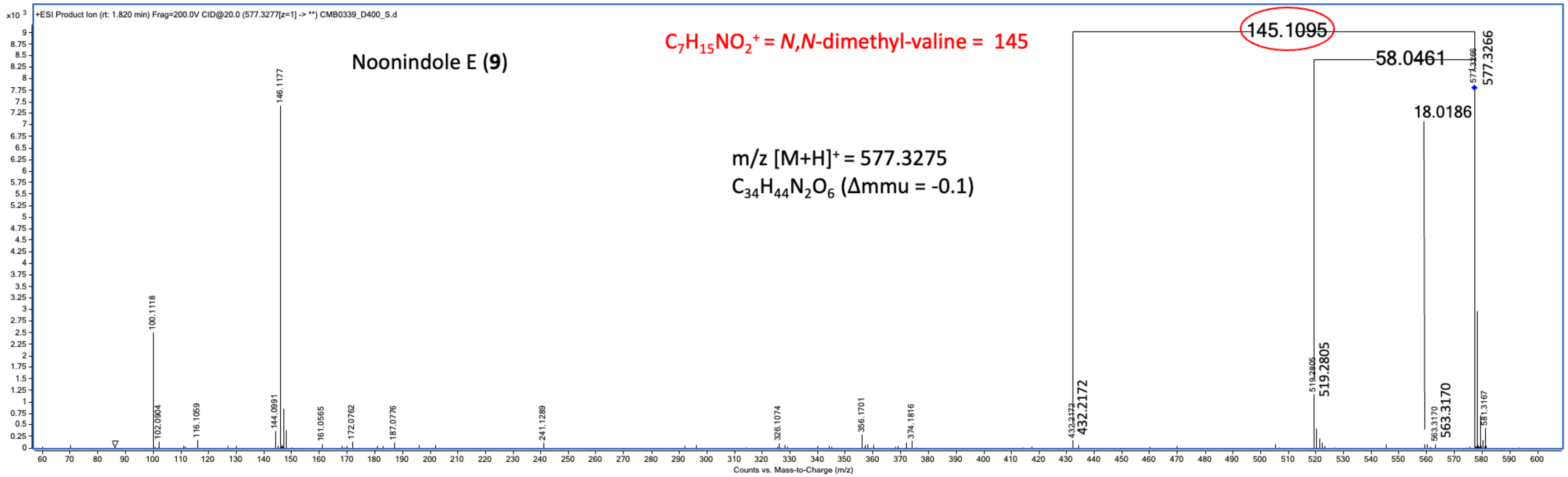

Figure S75. MS/MS fragmentation pattern of (**9**)

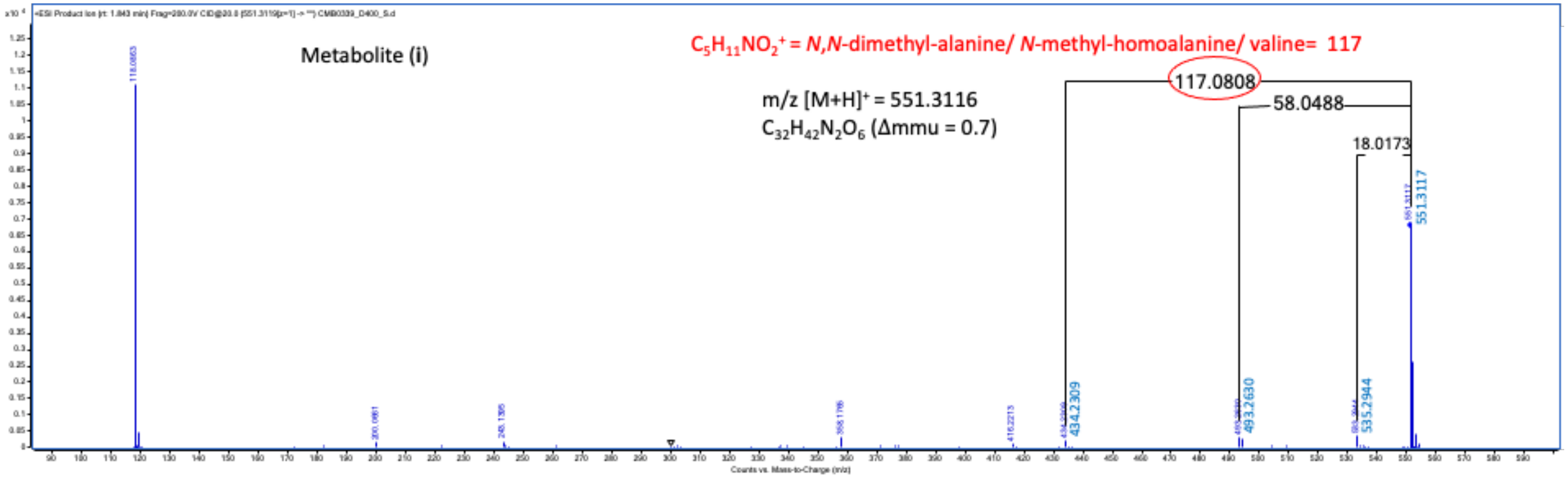

Figure S76. MS/MS fragmentation pattern of i

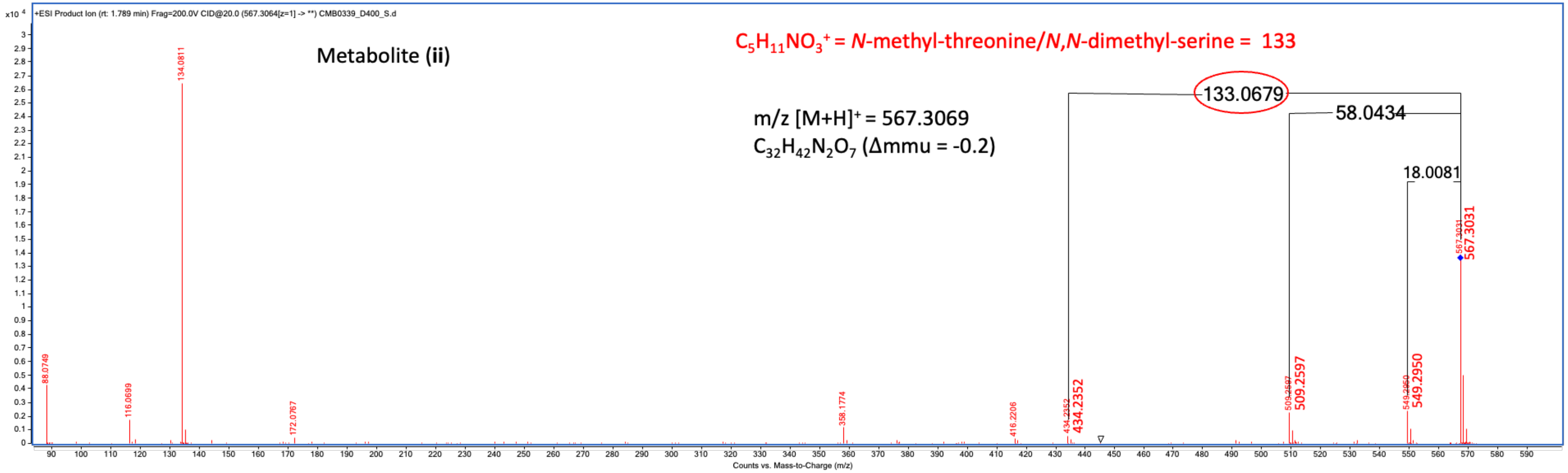

Figure S77. MS/MS fragmentation pattern of ii

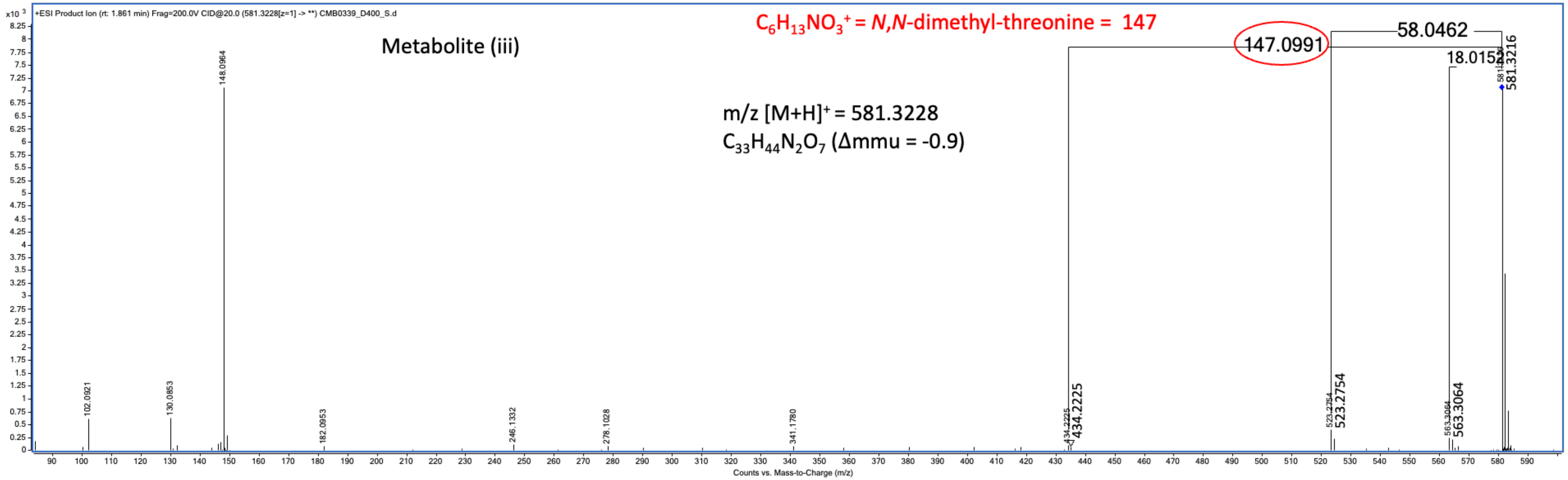

Figure S78. MS/MS fragmentation pattern of iii

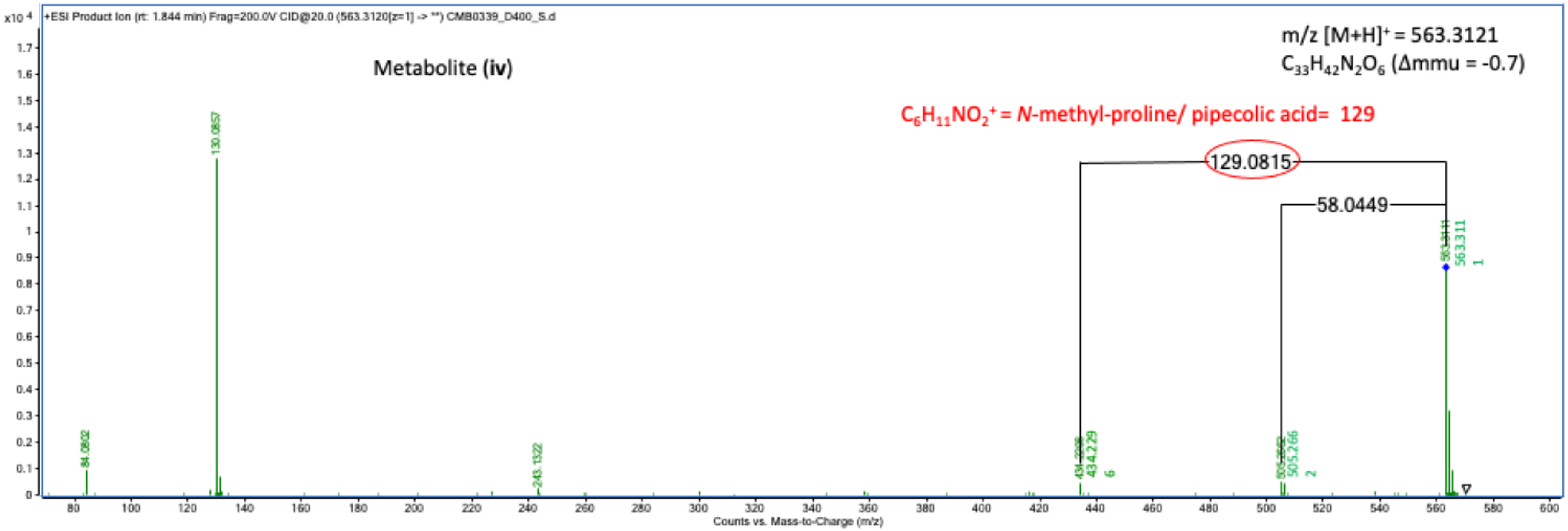

Figure S79. MS/MS fragmentation pattern of iv

Table S12. Molecular formulas generated for noonindoles A (**5**) -E (**9**) and predicted molecular formulas for minor metabolites (**i**) – (**viii**) observed in GNPS cluster

Metabolites		HRESI m/z $[M+H]^+$	HRESI MW	Predicted MF	ΔmDa
**Isolated metabolites**	**Minor metabolites detected in GNPS cluster**				
(**5**)		579.3437	578.3363	$C_{34}H_{46}N_2O_6$	-1.1
(**6**)/(**8**)		565.3278	564.3205	$C_{33}H_{44}N_2O_6$	-0.9
(**7**)		593.3590	592.3516	$C_{35}H_{48}N_2O_6$	-0.6
(**9**)		577.3275	576.3200	$C_{34}H_{44}N_2O_6$	-0.1
	(i)	551.3116	550.3051	$C_{32}H_{42}N_2O_6$	0.7
	(ii)	567.3069	566.2993	$C_{32}H_{42}N_2O_7$	-0.2
	(iii)	581.3228	580.3154	$C_{33}H_{44}N_2O_7$	-0.9
	(iv)	563.3121	562.3052	$C_{33}H_{42}N_2O_6$	-0.7
	(v)	593.3590	592.3516	$C_{35}H_{48}N_2O_6$	-0.6
	(vi)	609.3545	608.3472	$C_{35}H_{48}N_2O_7$	-1.7
	(vii)	591.3434	590.3362	$C_{35}H_{46}N_2O_6$	-0.9
	(viii)	595.3383	594.3309	$C_{34}H_{46}N_2O_7$	-0.6

15 Crystal data and structure refinement for noonindole A (5).

Identification code	2100_0339_p12	
Empirical formula	$C_{36} H_{46} F_3 N_2 O_8 . 3H_2O$	
Formula weight	746.80	
Temperature	190(2) K	
Wavelength	1.54184 Å	
Crystal system	Orthorhombic	
Space group	$P\ 2_1\ 2_1\ 2_1$	
Unit cell dimensions	a = 11.0176(2) Å	α= 90°.
	b = 19.8910(3) Å	β= 90°.
	c = 20.5726(2) Å	γ = 90°.
Volume	4508.51(12) $Å^3$	
Z	4	
Density (calculated)	1.100 Mg/m^3	
Absorption coefficient	0.750 mm^{-1}	
F(000)	1592	
Crystal size	0.200 x 0.200 x 0.200 mm^3	
Theta range for data collection	4.298 to 61.601°.	
Index ranges	-12<=h<=12, -22<=k<=22, -23<=l<=16	
Reflections collected	6976	
Independent reflections	6976 [R(int) = 0.0399]	
Completeness to theta = 61.602°	99.7 %	
Refinement method	Full-matrix least-squares on F^2	
Data / restraints / parameters	6976 / 6 / 475	

Goodness-of-fit on F^2	1.056
Final R indices [I>2sigma(I)]	R1 = 0.0332, wR2 = 0.0845
R indices (all data)	R1 = 0.0365, wR2 = 0.0863
Absolute structure parameter	-0.06(8)
Extinction coefficient	n/a
Largest diff. peak and hole	0.112 and -0.145 e.Å^{-3}

Table S13. Bond lengths [Å] and angles [°] for X-ray crystal structure of noonindole A (**5**)

C(1)-C(2)	1.379(3)	C(19)-C(20)	1.340(3)
C(1)-C(6)	1.392(3)	C(20)-C(21)	1.458(3)
C(2)-C(3)	1.396(4)	C(21)-O(2)	1.223(3)
C(3)-C(4)	1.378(3)	C(21)-C(22)	1.508(4)
C(4)-C(5)	1.399(3)	C(22)-O(1)	1.423(3)
C(5)-C(6)	1.427(3)	C(22)-C(23)	1.552(4)
C(5)-C(7)	1.427(3)	C(23)-O(5)	1.430(4)
C(6)-N(1)	1.382(3)	C(23)-C(24)	1.503(7)
C(7)-C(11)	1.363(3)	C(23)-C(25)	1.517(6)
C(7)-C(8)	1.508(3)	C(26)-O(4)	1.199(3)
C(8)-C(9)	1.548(3)	C(26)-O(3)	1.341(3)
C(9)-C(12)	1.511(3)	C(26)-C(27)	1.512(3)
C(9)-C(10)	1.565(3)	C(27)-N(2)	1.502(3)
C(10)-C(11)	1.512(3)	C(27)-C(28)	1.550(3)
C(10)-C(33)	1.542(3)	C(28)-C(30)	1.517(4)
C(10)-C(15)	1.562(3)	C(28)-C(29)	1.519(4)
C(11)-N(1)	1.376(3)	C(32)-N(2)	1.492(3)

C(12)-C(13)	1.524(3)	C(35)-O(8)	1.215(3)
C(13)-O(3)	1.466(2)	C(35)-O(7)	1.238(3)
C(13)-C(14)	1.530(3)	C(35)-C(36)	1.507(6)
C(14)-O(6)	1.428(3)	C(36)-F(3)	1.318(4)
C(14)-C(19)	1.526(3)	C(36)-F(1)	1.319(4)
C(14)-C(15)	1.589(3)	C(36)-F(2')	1.320(4)
C(15)-C(34)	1.537(3)	C(36)-F(1')	1.320(4)
C(15)-C(16)	1.539(3)	C(36)-F(2)	1.325(4)
C(16)-C(17)	1.543(3)	C(36)-F(3')	1.326(4)
C(17)-C(18)	1.535(3)	N(2)-C(31)	1.502(3)
C(18)-O(1)	1.435(3)	C(16)-C(15)-C(10)	112.06(16)
C(18)-C(19)	1.509(3)	C(34)-C(15)-C(14)	110.28(16)
C(2)-C(1)-C(6)	117.5(2)	C(16)-C(15)-C(14)	106.00(17)
C(1)-C(2)-C(3)	121.7(2)	C(10)-C(15)-C(14)	110.05(16)
C(4)-C(3)-C(2)	121.2(2)	C(15)-C(16)-C(17)	112.10(18)
C(3)-C(4)-C(5)	119.0(2)	C(18)-C(17)-C(16)	114.0(2)
C(4)-C(5)-C(6)	118.78(19)	O(1)-C(18)-C(19)	111.4(2)
C(4)-C(5)-C(7)	136.0(2)	O(1)-C(18)-C(17)	103.41(19)
C(6)-C(5)-C(7)	105.17(18)	C(19)-C(18)-C(17)	112.64(19)
N(1)-C(6)-C(1)	129.4(2)	C(20)-C(19)-C(18)	119.72(19)
N(1)-C(6)-C(5)	108.79(17)	C(20)-C(19)-C(14)	125.7(2)
C(1)-C(6)-C(5)	121.81(19)	C(18)-C(19)-C(14)	114.48(18)
C(11)-C(7)-C(5)	108.14(19)	C(19)-C(20)-C(21)	121.4(2)
C(11)-C(7)-C(8)	111.31(18)	O(2)-C(21)-C(20)	121.0(3)
C(5)-C(7)-C(8)	140.3(2)	O(2)-C(21)-C(22)	121.6(2)
C(7)-C(8)-C(9)	98.48(17)	C(20)-C(21)-C(22)	117.3(2)
C(12)-C(9)-C(8)	121.56(18)	O(1)-C(22)-C(21)	110.4(2)
C(12)-C(9)-C(10)	111.31(18)	O(1)-C(22)-C(23)	107.0(2)
C(8)-C(9)-C(10)	106.60(16)	C(21)-C(22)-C(23)	113.4(3)
C(11)-C(10)-C(33)	106.18(15)	O(5)-C(23)-C(24)	109.8(3)
C(11)-C(10)-C(15)	119.45(17)	O(5)-C(23)-C(25)	107.4(3)
C(33)-C(10)-C(15)	113.48(18)	C(24)-C(23)-C(25)	111.4(4)
C(11)-C(10)-C(9)	96.36(16)	O(5)-C(23)-C(22)	107.8(3)
C(33)-C(10)-C(9)	111.32(17)	C(24)-C(23)-C(22)	110.9(3)
C(15)-C(10)-C(9)	108.78(15)	C(25)-C(23)-C(22)	109.4(3)
C(7)-C(11)-N(1)	110.21(17)	O(4)-C(26)-O(3)	124.3(2)
C(7)-C(11)-C(10)	112.63(18)	O(4)-C(26)-C(27)	123.4(2)
N(1)-C(11)-C(10)	135.9(2)	O(3)-C(26)-C(27)	112.2(2)

C(9)-C(12)-C(13)	105.98(17)	N(2)-C(27)-C(26)	110.95(19)
O(3)-C(13)-C(12)	107.95(16)	N(2)-C(27)-C(28)	113.1(2)
O(3)-C(13)-C(14)	106.76(16)	C(26)-C(27)-C(28)	110.63(17)
C(12)-C(13)-C(14)	114.24(17)	C(30)-C(28)-C(29)	111.1(2)
O(6)-C(14)-C(19)	106.77(17)	C(30)-C(28)-C(27)	114.7(2)
O(6)-C(14)-C(13)	109.50(16)	C(29)-C(28)-C(27)	110.1(2)
C(19)-C(14)-C(13)	113.66(16)	O(8)-C(35)-O(7)	127.7(3)
O(6)-C(14)-C(15)	107.63(15)	O(8)-C(35)-C(36)	115.2(3)
C(19)-C(14)-C(15)	106.78(16)	O(7)-C(35)-C(36)	116.9(2)
C(13)-C(14)-C(15)	112.20(16)	F(3)-C(36)-F(1)	109.6(9)
C(34)-C(15)-C(16)	108.71(18)		
C(34)-C(15)-C(10)	109.67(17)		
F(2')-C(36)-F(1')	99.9(9)		
F(3)-C(36)-F(2)	111.6(13)		
F(1)-C(36)-F(2)	101.3(10)		
F(2')-C(36)-F(3')	110.5(13)		
F(1')-C(36)-F(3')	116.4(13)		
F(3)-C(36)-C(35)	114.0(6)		
F(1)-C(36)-C(35)	116.8(6)		
F(2')-C(36)-C(35)	111.4(5)		
F(1')-C(36)-C(35)	108.8(7)		
F(2)-C(36)-C(35)	102.5(8)		
F(3')-C(36)-C(35)	109.5(8)		
C(11)-N(1)-C(6)	107.67(17)		
C(32)-N(2)-C(31)	108.6(2)		
C(32)-N(2)-C(27)	111.9(2)		
C(31)-N(2)-C(27)	111.8(2)		
C(22)-O(1)-C(18)	115.32(18)		
C(26)-O(3)-C(13)	116.59(17)		

16 Biological activity of 5-14

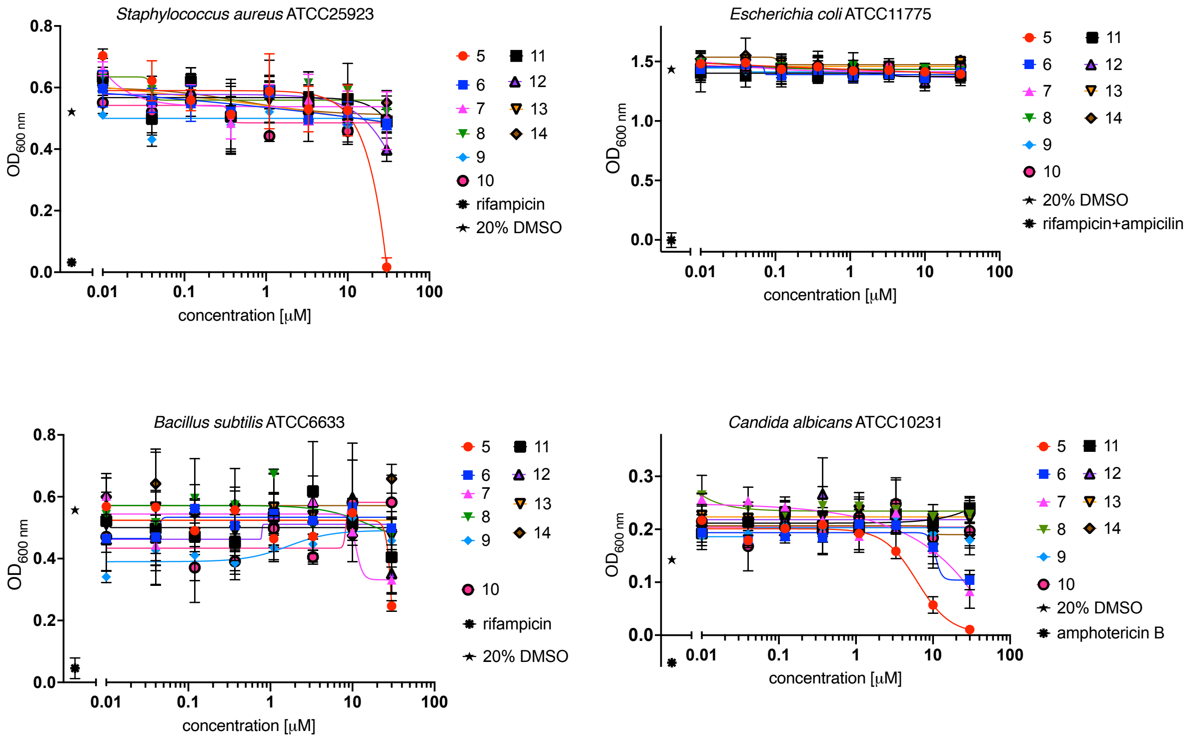

Figure S80. Antimicrobial activity of metabolites **5–14** against *S. aureus*, *E. coli*, *B. subtilis* and *C. albicans*

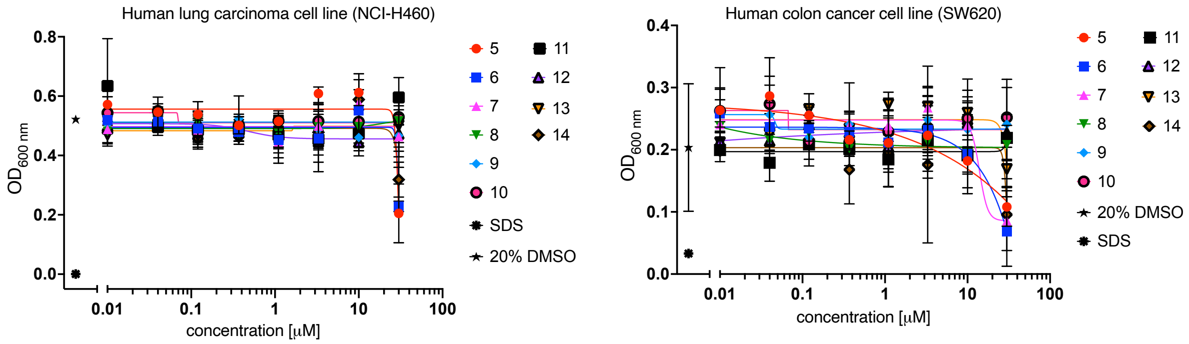

Figure S81. Cytotoxicity of metabolites **5–14** against human colon cancer (SW620) and human lung carcinoma (NCI-H460) cell lines

17 References

1. Fan, Y.; Wang, Y.; Liu, P.; Fu, P.; Zhu, T.; Wang, W.; Zhu, W. Indole-diterpenoids with Anti-H1N1 Activity from the Aceduric Fungus *Penicillium camemberti* OUCMDZ-1492. *J. Nat. Prod.* **2013**, *76*, 1328-1336.
2. Munday-Finch, S. C.; Wilkins, A. L.; Miles, C. O. Isolation of paspaline B, an indole-diterpenoid from *Penicillium paxilli*. *Phytochemistry* **1996**, *41*, 327-332.
